# Characterizing the effects of genetic liability to autoimmune conditions on pregnancy outcomes using Mendelian randomization

**DOI:** 10.1186/s12916-026-04797-w

**Published:** 2026-03-19

**Authors:** Elisabeth Aiton, Nancy S. McBride, Gemma L. Clayton, Ana Gonçalves Soares, Tom A. Bond, Qian Yang, Charikleia Chatzigeorgiou, Jane West, Benjamin G. Faber, Katherine Birchenall, Christy Burden, Maria C. Magnus, Deborah A. Lawlor, Maria Carolina Borges

**Affiliations:** 1 Population Health Sciences, Bristol Medical School, University of Bristol, Augustine’s Courtyard, Orchard Lane, Bristol, BS1 5DS UK; 2 MRC Integrative Epidemiology Unit at the University of Bristol, Bristol, UK; 3 Department of Endocrine and Metabolic Diseases, Shanghai Institute of Endocrine and Metabolic Diseases, Ruijin Hospital, Shanghai Jiao Tong University School of Medicine, Shanghai, China; 4 Shanghai National Clinical Research Center for Metabolic Diseases, Key Laboratory for Endocrine and Metabolic Diseases of the National Health Commission of the PR China, Shanghai Key Laboratory for Endocrine Tumor, Lifecycle Health Management Center, Ruijin Hospital, Shanghai Jiao Tong University School of Medicine, Shanghai, China; 5 School of Medicine and Population Health, University of Sheffield, Sheffield, UK; 6 Musculoskeletal Research Unit, University of Bristol, Bristol, UK; 7 Department of Obstetrics and Gynaecology, St Michael’s Hospital, Bristol, UK; 8 Translational Health Sciences, Bristol Medical School, University of Bristol, Bristol, UK; 9 School of Medicine and Public Health, Hunter Medical Research Institute, University of Newcastle, Callaghan, Australia; 10 Centre for Fertility and Health, Norwegian Institute of Public Health, Oslo, Norway

**Keywords:** Autoimmune conditions, Causality, Genetic, Mendelian randomization, Pregnancy

## Abstract

**Background:**

Autoimmune conditions are common in women of reproductive age. They are associated with an increased risk of adverse pregnancy outcomes; whether this is causal is unclear. Our aim was to explore the causal effects of genetic liability to autoimmune conditions on pregnancy outcomes.

**Methods:**

We conducted two-sample Mendelian randomization (MR) to estimate effects of genetic liability to ten autoimmune conditions on nine primary and seven secondary pregnancy outcomes. We used data from the MR-PREG collaboration including up to 714,889 pregnancies. Main analyses used the inverse variance weighted method to pool effects across genetic variants. Sensitivity analyses explored bias due to pleiotropic variants and fetal genetics.

**Results:**

We found evidence for 7 effects of autoimmune condition genetic liability on primary pregnancy outcomes that were robust across all sensitivity analyses. Higher genetic liability to Hashimoto’s thyroiditis was protective against large-for-gestational-age and increased the risk of hypertensive disorders of pregnancy (HDP) and preterm birth. For example, risk of preterm birth increased by 6% (OR = 1.06 (95%CI: 1.02, 1.11)) per doubling in log odds of Hashimoto’s thyroiditis.

Genetic liability to type 1 diabetes and systemic lupus erythematosus each increased the risk of preterm birth only. Higher genetic liability to rheumatoid arthritis increased the risk of HDP, while higher ankylosing spondylitis genetic liability reduced the risk of HDP. For multiple sclerosis, systemic sclerosis, coeliac disease, inflammatory bowel disease, and psoriasis, we did not detect any robust effects of increased genetic liability.

**Conclusions:**

We observed higher genetic liability to Hashimoto’s thyroiditis, type 1 diabetes, and rheumatic conditions causes increased risk of adverse pregnancy outcomes. Further research is warranted to explore the immune mechanisms underlying these relationships, and identify targets for prevention.

**Supplementary Information:**

The online version contains supplementary material available at 10.1186/s12916-026-04797-w.

## Background

Autoimmune conditions are increasing in incidence globally and are more common in women. In Europe, one in eight women is affected [1], and many are diagnosed before or during their reproductive years [1]. Pregnancy itself results in marked changes to the immune system which are essential to maintain tolerance to the fetus while remaining resilient to infections.

There is a complex interplay between autoimmune conditions and pregnancy. Pregnancy can profoundly influence the activity of autoimmune conditions. For example, patients with multiple sclerosis typically experience fewer relapses during pregnancy [2]. Pregnant women with autoimmune conditions are often at greater risk of developing adverse pregnancy outcomes in observational studies [3]. Rheumatic conditions, encompassing both systemic lupus erythematosus and rheumatoid arthritis, have been associated with increased risk of stillbirth, preeclampsia, and preterm birth [3]. Clinical hypothyroidism, most often caused by Hashimoto’s thyroiditis, is associated with higher risk of miscarriage, hypertensive disorders of pregnancy (HDP), and preterm birth [3, 4]. Type 1 diabetes is associated with a substantially increased risk of stillbirth and preterm birth [3], and inflammatory bowel disease is also associated with preterm birth [3] and admission to the neonatal intensive care unit (NICU) [5].

These observational associations may not reflect causal relationships and could instead be explained by residual confounding due to inadequate adjustment for maternal characteristics, for instance socioeconomic position, body mass index and comorbid autoimmune conditions. Even in systematic reviews with meta-analyses of observational studies, the associations between autoimmune conditions and pregnancy outcomes are often underpowered for rare outcomes like stillbirth, low Apgar score and admission to the NICU [3]. Research in this area is limited, and little evidence is available for some autoimmune conditions, such as systemic sclerosis, and some outcomes, such as gestational diabetes mellitus (GDM) [3]. Understanding the direct impacts of autoimmune conditions on pregnancy outcomes is critical to guide maternity care for affected women.

Mendelian randomization (MR) uses genetic variants to test the causal effect of potential risk factors on health outcomes [6]. Since genetic variants are randomly assorted at meiosis, MR mitigates bias due to reverse causation and common confounders, such as socioeconomic position [7]. Consistent with observational evidence, MR studies have suggested that genetic liability to rheumatoid arthritis and systemic lupus erythematosus may increase the risk of preterm birth and preeclampsia [8–10]. However, these studies have not comprehensively considered the impacts of the most common autoimmune conditions on a range of pregnancy outcomes, accounted for potential biases due to inherited fetal genetic variants, or explored the role of the pleiotropic human leukocyte antigen (HLA) genetic region which is associated with multiple autoimmune conditions.

Our aim was to determine potential causal effects of genetic liability to autoimmune conditions on pregnancy outcomes using MR. Because the genetic instruments capture liability to these conditions rather than their clinical manifestation, our findings should be interpreted qualitatively, consistent with previous MR studies of disease liability [11–13]. A precise, statistically robust effect suggests a likely causal relationship between genetic liability to the autoimmune condition and the pregnancy outcome, although the magnitude of the estimated effect is not directly clinically interpretable.

## Methods

We followed the Strengthening the Reporting of Observational Studies in Epidemiology using Mendelian Randomization (STROBE-MR) reporting guidelines [14] (Additional file 1).

### Study design

**Fig. 1:**
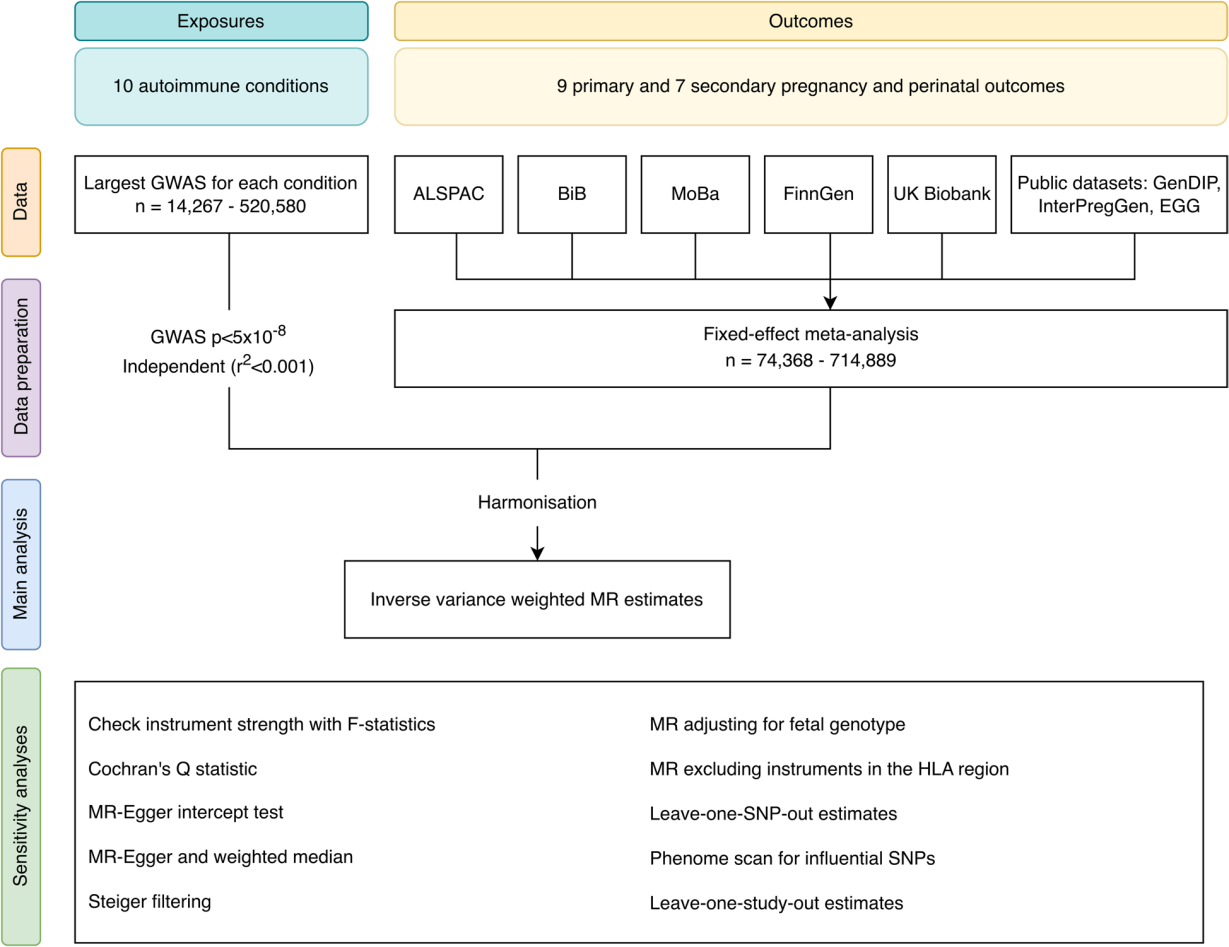
Outline of study design illustrating data sources, data preparation and analyses. GWAS = genome-wide association study, ALSPAC = Avon Longitudinal Study of Parents and Children, BiB = Born in Bradford, MoBa = the Norwegian Mother, Father and Child Cohort Study, GenDIP = GENetics of Diabetes In Pregnancy, InterPregGen = International Pregnancy Genetics study, EGG = Early Growth Genetics consortium, MR = Mendelian randomization, HLA = human leukocyte antigen, SNP = single nucleotide polymorphism

We used two-sample MR [6] to explore the causal effects of genetic liability to ten autoimmune conditions on nine prespecified primary and seven secondary pregnancy outcomes. Genetic association data for autoimmune conditions were obtained from previous genome-wide association studies (GWAS; *n* = 14,267–520,580). Genetic association data for pregnancy outcomes were obtained from the MR-PREG collaboration (*n* = 74,368–714,889) [15]. Figure 1 provides an overview of the study design.

### Autoimmune conditions

We selected autoimmune conditions on the basis that they may be diagnosed in women of reproductive age [1] and that there was at least one published GWAS of the condition with at least 5000 cases to ensure our MR analyses were well powered (Additional file 2: Fig. S1) [15–27]. We identified the largest GWAS for each condition on GWAS Catalogue [28] and OpenGWAS [29]. Further details of these GWAS [21, 22, 30–47] can be found in Table S1 (Additional file 3). This identified 10 conditions meeting our criteria (Additional file 2: Fig. S1): ankylosing spondylitis, celiac disease, Hashimoto’s thyroiditis, inflammatory bowel disease, multiple sclerosis, psoriasis, rheumatoid arthritis, systemic lupus erythematosus, systemic sclerosis, and type 1 diabetes.

### Instrument selection

For each GWAS, we selected genetic variants (single nucleotide polymorphisms; SNPs) strongly associated with the respective autoimmune condition (*p*-value < 5 × 10^−8^). These were clumped to select independent SNPs (linkage disequilibrium threshold R^2^ < 0.001, clumping window = 10,000 kb) using the 1000 genomes European super population reference panel. For systemic sclerosis, where full GWAS data were not available, we used the independent SNPs identified in the original study using stepwise joint conditional association analyses in 1.5 Mb windows around the lead SNP.

### Pregnancy outcomes

We explored potential effects on nine primary pregnancy outcomes, selected based on clinical importance and previously reported associations [3–5, 48]: miscarriage, stillbirth, HDP, GDM, preterm birth, small-for-gestational-age, large-for-gestational-age, low Apgar score at 5 min, and NICU admission. We also assessed seven secondary outcomes, including subtypes of primary outcomes (e.g. preeclampsia and gestational hypertension) and underlying continuous traits (e.g. birthweight) to improve statistical power and help interpretation of findings. Outcome genetic association data were obtained from the MR-PREG collaboration, which integrates data from five cohort studies and four publicly available GWAS [15]. Outcome definitions and sample sizes can be found in Tables S2A-B (Additional file 3), and cohort descriptions in Supplementary methods (Additional file 2).

### Statistical analysis

Two-sample MR and data harmonization were conducted using the TwoSampleMR R package [29]. All analyses were conducted in R (4.5.0). All code and a pre-specified analysis plan (dated 12/02/2024) are available at https://github.com/eaiton/autoimmune-pregnancy.

Our main analyses used the random-effects inverse variance weighted (IVW) estimator. Effect estimates reflect odds ratios (binary outcomes) or differences in mean standard deviation units (continuous outcomes). To aid interpretability effect estimates and standard errors were scaled to reflect a doubling in genetic liability to the autoimmune condition, by multiplying these by log_e_2, before estimating 95% confidence intervals [49]. Given that our binary outcomes are relatively rare, we assume odds and risks of outcomes are interchangeable.

We identified potential causal effects for follow-up with sensitivity analyses if they met either of the following criteria: (i) statistical support at *p* < 0.05, or (ii) relative effect estimates 5% or greater (i.e. odds ratio > 1.05 or < 0.95) with statistical support at *p* < 0.10. We used these criteria since selecting results based on statistical significance alone is inappropriate in a hypothesis-driven context [50], where we have a strong biological prior on the basis of observational studies, the outcomes of interest are clinically important yet relatively rare, and modest causal effects may still be meaningful. We additionally report whether effects passed multiple testing correction using the Benjamini-Yekutieli false discovery rate (alpha = 0.05) [51] to account for arbitrary dependence between tests due to shared genetic risk loci between autoimmune conditions [52] and correlations between pregnancy outcomes. We explored the extent of shared genetic risk by examining instrument correlations between conditions (Additional file 2: Supplementary methods).

We undertook a series of additional and sensitivity analyses to explore MR assumptions and potential bias in our main analyses. Instrument strength was estimated using the mean pseudo F-statistic across SNPs for each autoimmune condition (Additional file 2: Supplementary methods). We explored between SNP heterogeneity using Cochran’s Q-statistics and by inspecting scatter plots of SNP-exposure and SNP-outcome effects. We tested for unbalanced horizontal pleiotropy using the MR-Egger intercept test, and compared main MR estimates to two pleiotropy-robust MR methods: MR-Egger and weighted median. We explored the direction of effects by performing Steiger filtering to remove SNPs which explained more variance in an outcome than in the condition (*p* < 0.05) [53], and generating estimates with the remaining SNPs using either the inverse-variance weighted estimator or the Wald ratio.

Inherited fetal genetic variants could bias our results if they affect pregnancy outcomes independently of the maternal genetic variant. For example, if a maternal variant we instrumented was inherited by the fetus and then had direct effects on fetal growth and fetal risk of being born large-for-gestational-age, the effect of that variant on this outcome would be attributable to fetal genetic inheritance rather than via maternal autoimmune condition liability [54]. We explored this potential source of bias by comparing the main unadjusted results to results with adjustment for fetal genotype. First, we generated a GWAS estimating the associations between fetal genotype at each SNP and all available outcomes, using offspring genotypes available in three cohorts: the Avon Longitudinal Study of Parents and Children (ALSPAC), Born in Bradford (BiB) and the Norwegian Mother, Father and Child Cohort Study (MoBa). We meta-analyzed these with publicly available GWAS [55–57]. We then generated genetic association data adjusted for fetal genotype by combining this fetal genotype GWAS with the main maternal genotype GWAS in a weighted linear model, as described in the Supplementary methods (Additional file 2). We also investigated whether specific studies in our outcome data were driving results through leave-one-study-out analyses, and estimated potential sample overlap between exposure and outcome GWAS as detailed in Supplementary methods (Additional file 2).

We identified influential SNPs using leave-one-SNP-out analyses. We identified potential pleiotropic effects of any influential SNPs through a phenome-wide association scan, using OpenGWAS [29]. Finally, we investigated the role of the HLA genetic region on our results, given variants in this region are associated with some of the autoimmune conditions we are exploring, and this region is highly pleiotropic and has an extensive linkage disequilibrium structure [52]. To do this, we repeated analyses removing SNPs located within the HLA region, defined as chr6:28,477,797–33,448,354 (genome reference build GRCh37).

## Results

Among the ten autoimmune conditions, the mean pseudo-F-statistic across genetic instruments ranged from 54 for systemic sclerosis to 286 for celiac disease (Additional file 3: Table S3). The correlation of instrument effects between conditions is summarised in Figs. S2-S6 (Additional file 2). Correlations were highly varied; for instance, type-1 diabetes instruments were poorly correlated with inflammatory bowel disease genetic liability (correlation coefficient = -0.15) but strongly positively correlated with rheumatoid arthritis genetic liability (correlation coefficient = 0.76). Correlations across conditions tended to be more positive after excluding SNPs in the HLA region (Additional file 2: Fig. S3). Harmonized condition-outcome data are provided in Table S4 (Additional file 3).

**Fig. 2:**
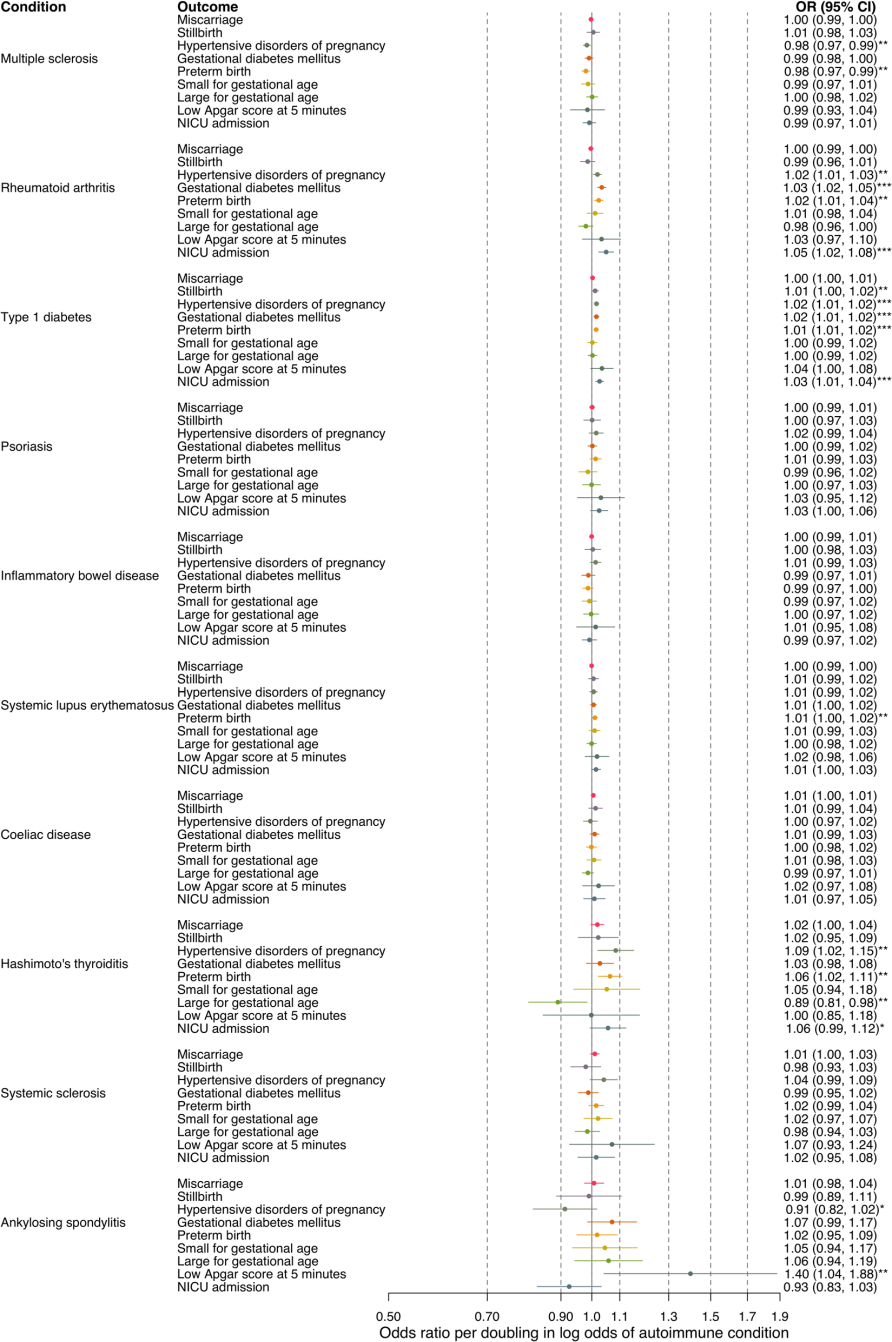
Mendelian randomization estimates for the effect of increased genetic liability to autoimmune conditions on primary pregnancy outcomes. Estimates reflect the odds ratio of a pregnancy outcome per doubling in the log odds of an autoimmune condition. NICU = neonatal intensive care unit. * indicates *p* < 0.1 and change in odds of 5% or more, ** indicates *p* < 0.05, *** indicates *p* < 0.05 after correcting for multiple testing using the false discovery rate

Main findings for primary outcomes are shown in Fig. 2 and for secondary outcomes in Figs. S7-S8 (Additional file 2). Results for all condition-outcome relationships and all sensitivity analyses are provided in Tables S5-S10 (Additional file 3), for effects both per doubling in log odds of autoimmune conditions (as used in main manuscript) and per 1-unit log-odds increase.

We identified 18 putative causal relationships for follow-up (Table 1): 16 with statistical support at *p* < 0.05, and two with a ≥ 5% change in odds and statistical support at *p* < 0.1. The conclusion from the main and sensitivity analyses for the 18 results selected for follow-up is summarized in Table 1 and described in detail below under the " Sensitivity analyses" section. Among these selected results, we found supporting evidence across all sensitivity analyses for 7, and uncertain evidence in one or more sensitivity analyses for 11. Below, we describe results for these 7 estimated causal effects with consistently supportive evidence.

We observed a lower risk of one adverse pregnancy outcome related to higher genetic liability to both Hashimoto’s thyroiditis and ankylosing spondylitis. Higher genetic liability to Hashimoto’s thyroiditis reduced the risk of having a large-for-gestational-age baby, with an odds ratio of (0.89 (95%CI:0.81, 0.98)). Higher liability to ankylosing spondylitis was related to a lower risk of HDP (0.91 (0.82, 1.02)).

Conversely, we found a higher risk of at least one adverse pregnancy outcome with higher genetic liability to rheumatoid arthritis, type 1 diabetes, systemic lupus erythematosus, and Hashimoto’s thyroiditis. Higher genetic liability to rheumatoid arthritis robustly increased the risk of HDP (1.02 (95% CI:1.01, 1.03)). Type 1 diabetes liability increased the risk of preterm birth (1.01 (95% CI:1.01, 1.02)), as did systemic lupus erythematosus (1.01 (95% CI:1.00, 1.02)). Higher Hashimoto’s thyroiditis genetic liability increased the risk of both HDP (1.09 (95% CI:1.02, 1.15)) and preterm birth (1.06 (1.02, 1.11)).

### Secondary outcomes

All conditions that affected the risk of combined HDP in our primary analysis (rheumatoid arthritis, Hashimoto’s thyroiditis, and ankylosing spondylitis) also consistently affected preeclampsia and gestational hypertension as separate outcomes in the same direction (Additional file 2: Fig. S7). For example, higher Hashimoto’s thyroiditis genetic liability increased the risks of both preeclampsia (1.10 (95% CI:1.02, 1.19)) and gestational hypertension (1.08 (95% CI:1.03, 1.14)).

**Table 1:**
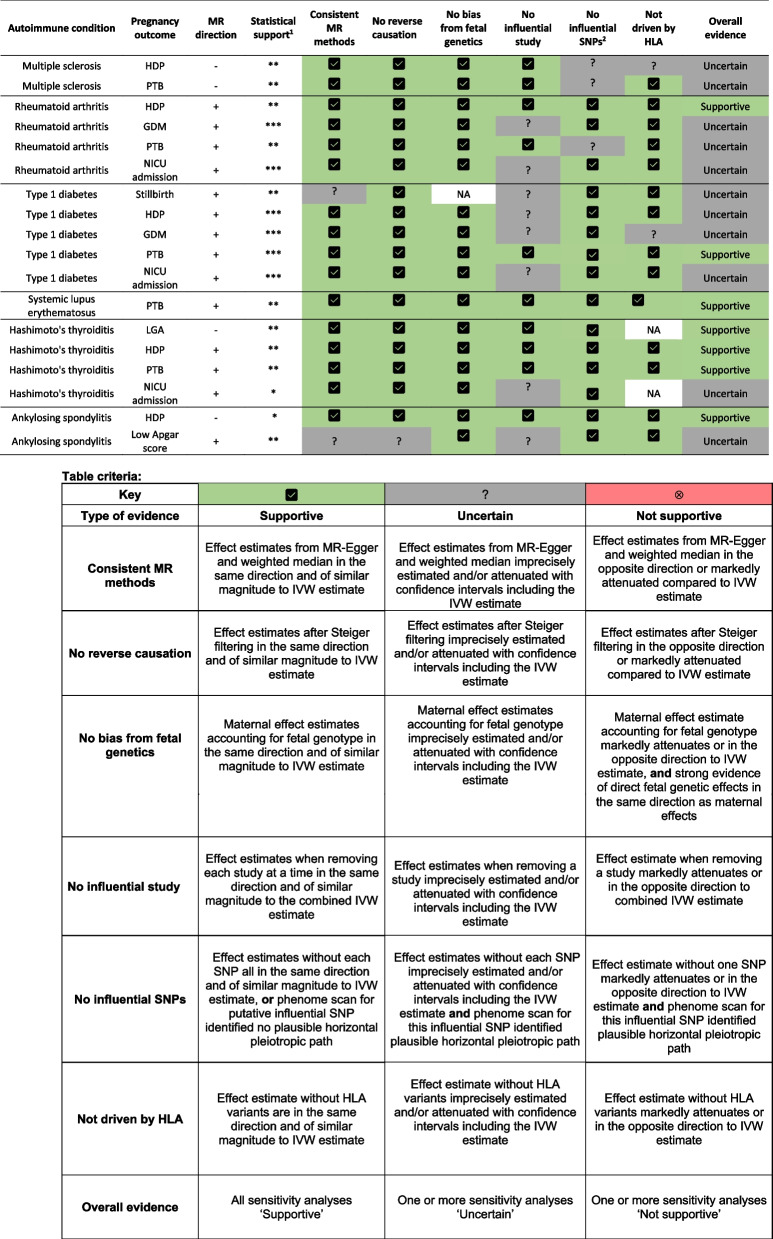
Main analysis results for follow-up and summary of sensitivity analyses

^1^Statistical support at * indicates *p* < 0.1 and an odds ratio change of 5% or greater, ** indicates *p* < 0.05, *** indicates *p* < 0.05 after correcting for multiple testing using false discovery rate

^2^Influential SNPs were identified in the leave-one-SNP-out analyses and a phenome scan was conducted to identify whether putative influential SNPs may be invalid due to plausible horizontal pleiotropic pathways. Cells stating ‘NA’ indicate where analyses could not be run since no SNPs were available in the outcome dataset, or the outcome was not available

*IVW* inverse variance weighted estimate

We similarly found that higher genetic liability to type 1 diabetes, systemic lupus erythematosus, and Hashimoto’s thyroiditis shortened mean gestational age at birth and lowered birthweight (Additional file 2: Fig. S8). These findings support our primary results, which show that genetic liability to these conditions increased the risk of preterm birth. Additionally, our finding that Hashimoto’s thyroiditis genetic liability may lower birthweight aligns with our primary finding that it reduces the risk of having a large-for-gestational-age baby. Effects of Hashimoto’s thyroiditis genetic liability on high birthweight and low birthweight were also directionally concordant with a lowered birthweight, though 95% confidence intervals were imprecise for these outcomes (Additional file 2: Fig. S7).

For conditions that increased risk of preterm birth (type 1 diabetes, systemic lupus erythematosus, Hashimoto’s thyroiditis), we observed similar increases in the risk of spontaneous preterm birth as a secondary outcome (Additional file 2: Fig. S7).

### Sensitivity analyses

We conducted several sensitivity analyses to assess the robustness of the 18 condition-primary outcome relationships that we identified for follow-up. These analyses are summarized in Table 1.

Evidence of heterogeneity between genetic variants (SNPs) was observed for 12 of the 18 condition-outcome relationships (*p* < 0.05; Additional file 3: Table S11). However, scatter plots did not indicate any clear outlying SNPs (Additional file 2: Figs. S9-S25) and effect estimates using pleiotropy-robust MR methods, MR-Egger and weighted median, were largely compatible with main IVW results (Additional file 2: Fig. S26). MR-Egger intercepts suggested potential unbalanced pleiotropy affected two relationships (*p* < 0.05): type 1 diabetes genetic liability on stillbirth (*p* = 0.032, intercept = 7.00 × 10^–3^) and on preterm birth (*p* = 0.034, intercept = −4.37 × 10^–3^). MR-Egger effect estimates remained compatible with main IVW estimates for the effect of type 1 diabetes genetic liability on preterm birth, but the effect on stillbirth was imprecisely attenuated. Steiger tests found that instruments explained more variation in conditions than in outcomes (Additional file 3: Table S12), except for five ankylosing spondylitis instruments with some outcomes. Steiger filtering did not change any effect estimates except for ankylosing spondylitis where the effect of increased genetic liability on low Apgar score at 5 min was less precise, as expected given the lower number of instruments available (Additional file 2: Fig. S27).

After adjustment for fetal genotypes, our effect estimates were directionally consistent with the main unadjusted results. However, because of the smaller sample size our estimates were less precise (Additional file 2: Fig. S28). Leave-one-study-out analyses were largely compatible with main meta-analysis results (Additional file 2: Figs. S29-S35). All effects on GDM and NICU admission were uncertain without the largest contributing cohort, as were effects of genetic liability to type 1 diabetes on stillbirth, type 1 diabetes on HDP, and ankylosing spondylitis on low Apgar score at 5 min. There was no sample overlap between most condition and outcome GWAS, except for Hashimoto’s thyroiditis (0–50.6%) and type 1 diabetes (0–38.5%; see Additional file 3: Tables S13A-B for overlap by outcome).

Of the 18 condition-outcome relationships selected for follow-up, three may have been biased by a single influential SNP, where removing it imprecisely attenuated the effect estimate with confidence intervals overlapping the main IVW estimate (Additional file 2: Figs. S36-S53). One SNP putatively influenced effects of multiple sclerosis genetic liability on both HDP and preterm birth, while a second SNP potentially influenced effects of rheumatoid arthritis genetic liability on preterm birth. Both located in the HLA region, phenome-wide association scans identified that these SNPs were associated with a range of immune and non-immune traits (Additional file 3: Table S14; Additional file 2: Figs. S54-S55), including height in a direction consistent with plausible horizontal pleiotropy for these outcomes (Table 2) [58–62]. For four additional relationships with putative influential SNPs, we found no evidence supporting pleiotropic pathways (Table 2; Additional file 2: Figs. S56-S58).

**Table 2:** Putative influential SNPs driving the effects of autoimmune condition genetic liability on pregnancy outcomes, and selected relevant associated traits from phenome-wide association scans

Autoimmune condition	Pregnancy outcome(s)	Influential SNP	SNP position	Effect allele, other allele	Immune-related phenome scan traits	Non-immune phenome scan traits	Direction of bias from horizontal pleiotropy
Multiple sclerosis	**HDP, preterm birth**	rs2857700	6:31,572,481 (HLA locus)	T, C	Reduced liability to type 1 diabetes, psoriasis, RA, hypothyroidism, celiac disease, ankylosing spondylitis, asthma Reduced anti-Merkel cell polyomavirus IgG seropositivity Increased Epstein-Barr virus antibody levels Increased monocyte percentage of white cells, and leukocyte count. Decreased neutrophil, eosinophil, lymphocyte, white blood cell count Reduced complement C2, complement factor B, C-reactive protein, glycoprotein acetyl levels	**Increased height** Increased lung function Increased SHBG levels Reduced LDL cholesterol levels, triglyceride, apolipoprotein B Reduced bilirubin and alanine aminotransferase levels	**Increased height reduces risk of HDP so may have biased this effect ** [58] **Increased height increases gestational duration so may have biased protective preterm birth effect ** [59]
Rheumatoid arthritis	**Preterm birth,** GDM	rs9275183	6:32,654,502 (HLA locus)	G, A	Increased liability to type 1 diabetes, primary biliary cholangitis, Grave’s disease. Reduced liability to primary sclerosing cholangitis, ulcerative colitis, inflammatory bowel disease, Still’s disease, asthma, systemic lupus erythematosus, celiac disease Increased membranous nephropathy. Increased use of immunosuppressant medication Increased Epstein-Barr virus antibodies. Increased liability to pulmonary tuberculosis Increased white blood cell, neutrophil counts. Increased eosinophil counts and percentage Increased CRP. Reduced complement factor B	**Reduced height** **Reduced BMI** Reduced lung function Increased apolipoprotein A, apolipoprotein B, HDL cholesterol. Reduced LDL cholesterol Increased aspartate aminotransferase levels	**Reduced height decreases gestational duration so may bias the adverse effect on preterm birth ** [59]**.** BMI is likely to have non-linear effects on risk of preterm birth so direction of bias uncertain [60] Reduced BMI decreases GDM risk [60], so here this allele would be expected to bias this relationship towards a negative effect. No plausible pleiotropic paths identified for GDM
Type 1 diabetes	Preterm birth	rs1794269	6:32,673,894 (HLA locus)	T, C	Reduced liability to psoriasis, inflammatory bowel disease. Increased liability to rheumatoid arthritis, celiac disease, primary biliary cholangitis, systemic lupus erythematosus, hypothyroidism, Grave’s disease, Sjogren’s syndrome, autoimmune diseases, myasthenia gravis History of infection with a predominantly sexually transmitted infection. Increased pulmonary tuberculosis Reduced Epstein-Barr virus antibodies. Increased varicella zoster virus antibodies Reduced complement C4 and factor B, increased complement C2	Reduced BMI Increased aspartate aminotransferase. Increased glomerular disorders. Reduced bilirubin Increased SHBG levels	No evidence for causal effects of BMI on preterm birth [60]. BMI may have non-linear effects on risk of PTB so direction of bias uncertain [60]
Hashimoto’s thyroiditis	Large-for-gestational-age	rs3184504	12:111,884,608	T, C	Increased liability to type 1 diabetes, asthma, inflammatory bowel disease, rheumatoid arthritis, and autoimmune diseases Increased spleen volume Increased eosinophil counts and percentage, increased white blood cell count, increased basophil count, reduced neutrophil levels. Increased platelet count and plateletcrit, haemoglobin concentration, and red blood cell count	Increased risk of hypertension, increased diastolic and systolic blood pressure Decreased glomerular filtration rate. Increased bilirubin and urate. Increased alanine aminotransferase levels Reduced HDL cholesterol levels, reduced total cholesterol. Reduced apolipoprotein A Reduced risk of breast cancer	Increased maternal blood pressure reduces the risk of LGA [61] so would be expected to bias this relationship towards a negative effect Maternal glomerular filtration rate may be positively associated with birthweight [62], so would be expected to bias this relationship towards a negative effect No plausible pleiotropic paths
Hashimoto’s thyroiditis	NICU admission	rs6679677	9:100,535,267	A, C	Increased liability to type 1 diabetes, rheumatoid arthritis, Still disease, Graves’ disease, asthma, systemic lupus erythematosus, and autoimmune traits. Reduced liability to Crohn’s disease Increased use of immunosuppressant medications Reduced lymphocyte, neutrophil, monocyte and white blood cell counts. Reduced lymphocyte percentage	Reduced serum alkaline phosphatase Reduced glomerular filtration rate. Increased cystatin C levels Reduced risk of skin cancer	No plausible pleiotropic paths

Bold text highlights where non-immune traits may lie on horizontal pleiotropic pathways since they are known determinants of the pregnancy outcome, in the expected direction to have biased main results for one or more outcomes

Effects on other traits are all given in terms of the effect allele increasing liability to the autoimmune condition exposure of interest. Traits were selected based on relevance, removing duplicates, the exposure and the outcome(s) of interest and medications relevant to these. SNPs which were deemed ‘uncertain’ but possibly influential were included here

*SHBG* sex hormone-binding globulin, *LDL* low density lipoprotein, *HDL* high density lipoprotein, *CRP* C-reactive protein, *NICU* neonatal intensive care unit

Across autoimmune conditions, the number of instrumented SNPs in the HLA region ranged from none (systemic sclerosis) to 11 (type 1 diabetes, *n* = 11/89 (12%). Excluding these HLA SNPs reduced precision but largely produced effect estimates of similar magnitude and direction to the main IVW analysis (Additional file : Fig. S26). Estimated effects of higher genetic liability to multiple sclerosis on HDP and of type 1 diabetes on GDM were imprecisely attenuated after excluding HLA SNPs.

Of the 18 condition-primary outcome relationships followed up, the 7 causal effects which were robust across sensitivity analyses are cross-tabulated by condition and affected outcomes in Table 3.

**Table 3:** Summary of robust effects across sensitivity analyses

		Outcome			
Classification	Condition	HDP	Preterm birth	Large-for-gestational-age	
Metabolic	Hashimoto’s thyroiditis	+	+	-	3
Endocrine	Type 1 diabetes		+		1
Rheumatic	Rheumatoid arthritis	+			1
	Ankylosing spondylitis	-			1
	Systemic lupus erythematosus		+		1
		3	3	1	7

- indicates that higher liability has a protective effect on risk of the outcome

+ indicates that higher liability has an adverse effect on risk of the outcome

Numbers are counts of robust effects for each condition (rows) and each outcome (columns)

*HDP* hypertensive disorders of pregnancy

## Discussion

Previous conventional multivariable regression analyses have mostly focused on relationships between a limited number of autoimmune conditions and adverse pregnancy outcomes [2–5]. By contrast we have explored potential causal effects of genetic liability to a range of autoimmune conditions on numerous adverse pregnancy outcomes.

Our results showed a diverse pattern of effects across conditions and outcomes. For several conditions, increased liability only resulted in higher risk of adverse outcomes (type 1 diabetes, rheumatoid arthritis, systemic lupus erythematosus). For Hashimoto’s thyroiditis, higher genetic liability resulted in both increased and decreased risk of adverse pregnancy outcomes, while higher genetic liability to ankylosing spondylitis reduced risk of HDP only. For other conditions, we found uncertain or unsupportive evidence of causal effects on the outcomes assessed (multiple sclerosis, systemic sclerosis, celiac disease, inflammatory bowel disease and psoriasis).

These findings are consistent with previous MR studies which have estimated effects of genetic liability to certain autoimmune conditions on pregnancy outcomes in FinnGen, one of our contributing cohorts [8–10, 63]. Reassuringly, previous studies also identified adverse causal effects of rheumatoid arthritis genetic liability on hypertensive disorders of pregnancy [8–10], and systemic lupus erythematosus genetic liability on preterm birth [8], though these studies had a lower multiple testing burden and used a different scale for effect estimates to the present study.

Direct comparison between our study and previous observational studies is difficult due to differences in the number of autoimmune conditions and pregnancy outcomes analyzed, statistical power, and the nature of the effects estimated. Our causal estimates represent effects of a doubling in lifetime genetic liability to an autoimmune condition in the general population, so cannot be directly compared with observational studies considering the effects of a clinical diagnosis. We therefore recommend interpretation of effects based on their direction rather than their magnitude, which has no direct clinical interpretation.

In line with our findings, existing observational studies have reported adverse associations for Hashimoto’s thyroiditis with HDP and preterm birth; type 1 diabetes with preterm birth; rheumatoid arthritis with HDP, and systemic lupus erythematosus with preterm birth [3, 64]. In contrast, our findings that genetic liability to Hashimoto’s thyroiditis decreased risk of large-for-gestational-age [65] and that ankylosing spondylitis decreased risk of HDP [66] were not found observationally. These differences between our MR findings and previous observational studies may be attributable to differences in the exposures under investigation, differences in power due to small cohorts of women diagnosed with rarer autoimmune conditions, and/or to factors such as treatment effects or residual confounding influencing the observational associations.

Increased genetic liability to rheumatic autoimmune conditions may affect pregnancy outcomes through two key mechanisms: elevated inflammation and autoantibodies [67]. An imbalance of increased pro-inflammatory CD4 + T cells and decreased T-regulatory cells is associated with the shallow trophoblast invasion seen in preeclampsia [68]. Elevated maternal inflammation is also an established cause of preterm birth, in particular via pro-inflammatory cytokines IL-1 and TNF-α [69]. Antiphospholipid autoantibodies, often present in women with systemic lupus erythematosus and rheumatoid arthritis [70], are involved in inflammatory placental damage and are associated with elevated risks of both HDP and preterm birth [67, 71]. These autoantibodies are also more prevalent in women who have experienced recurrent pregnancy losses [72], suggesting potential roles in the etiology of further adverse pregnancy outcomes.

Genetic liability to Hashimoto’s thyroiditis may likewise affect adverse pregnancy outcomes through the development of autoantibodies to thyroid peroxidase, thyroglobulin, or thyroid-stimulating hormone receptors [73]. Present in 3–18% of pregnant women, these autoantibodies are associated with increased risk of preterm birth even in euthyroid pregnancies [73].

Adverse effects of type 1 diabetes could be mediated by insulin insufficiency and immune dysregulation. Hyperglycaemia is an established risk factor for adverse pregnancy outcomes, even below diagnostic thresholds [59]. Immune dysregulation characteristic of type 1 diabetes, such as an increased Th1/Th2 ratio [74], may be antagonistic to the immune tolerance seen in a healthy pregnancy; this may in turn affect risks of preterm birth [75].

Through the systematic exploration of a range of autoimmune conditions and relevant pregnancy outcomes, we provide a new line of comprehensive and novel causal evidence on these. Our MR analysis should be less susceptible to conventional observational confounding [7] and reverse causation [64]. The strengths of our approach include our use of large-scale genetic cohort data to maximize statistical power, and the consideration of bias due to fetal genetic effects, the HLA region, reverse causation, and horizontal pleiotropy.

Since our exposure is genetic liability to a condition, rather than clinical diagnosis, our results could reflect the effects of underlying risk factors for an autoimmune condition rather than direct effects of the condition itself [76]. These risk factors could include obesity and health-related behaviours such as smoking [77]. This is particularly pertinent for conditions with lower prevalence [49, 76], for instance systemic sclerosis and ankylosing spondylitis. We addressed this by assessing evidence for horizontal pleiotropy by exploring between-SNP heterogeneity, using pleiotropy-robust MR methods, performing Steiger filtering, excluding HLA variants, and conducting a phenome scan for influential SNPs.

Autoimmune conditions often have shared genetic architecture [52, 78], which warrants caution when interpreting effect estimates for their genetic liabilities. For instance, we observed that higher genetic liability to ankylosing spondylitis was associated with a reduced risk of HDP, whereas higher liability to Hashimoto’s thyroiditis was associated with an increased risk. However, the genetic instruments for these conditions were negatively correlated: as an example, among the 25 SNPs selected as instruments for ankylosing spondylitis, alleles that increased ankylosing spondylitis risk tended to decrease the risk of Hashimoto’s thyroiditis (correlation coefficient = − 0.42). Such cross-condition correlations may induce spurious positive or negative effect estimates or obscure true causal effects, particularly when liabilities to correlated autoimmune conditions differentially influence the outcome.

Our apparent small effect sizes are likely the result of the fact that the conditions we investigate are rare [1]. Importantly, as noted previously, analyses of genetic liability do not reflect the magnitude of effect of a clinical diagnosis, meaning the magnitude cannot be interpreted clinically. For some rarer outcomes, such as low Apgar score at 5 min, we lack power. Even in large cohort studies of diagnosed patients, the increased risk of adverse pregnancy outcomes associated with autoimmune conditions is often small to moderate (with the exception of type 1 diabetes [64]), so detecting causal effects using genetic liability may require extremely large sample sizes, particularly for systemic sclerosis, inflammatory bowel disease, celiac disease and psoriasis [64].

Eight autoimmune conditions of interest could not be included here because no sufficiently large GWAS was publicly available. Moreover, no autoimmune condition GWAS were stratified by sex, so we could not explore whether potential sex differences in instruments might bias our results. Except ankylosing spondylitis and type 1 diabetes, all conditions analyzed here are more common in women, which will mitigate this potential source of bias to some extent [1]. Selection bias may also have affected our findings because some autoimmune conditions are associated with reduced fertility [64]. Since our study was restricted to women who have had at least one pregnancy, this could introduce collider bias [79]. We restricted to cohorts of individuals with majority white European ancestry, so further research is needed to assess whether these results are generalisable to non-white European ancestry groups. Sample overlap between GWAS of Hashimoto’s thyroiditis and type 1 diabetes and pregnancy outcome GWAS could bias estimates towards the (confounded) observational estimate [80]. We explored this through leave-one-study-out analyses.

## Conclusions

In conclusion, our study supports that increased genetic liability to Hashimoto’s thyroiditis, type 1 diabetes, rheumatoid arthritis, and systemic lupus erythematosus increases the risk of adverse pregnancy outcomes. Causal effects were particularly centred on HDP and preterm birth. Understanding whether reported associations between autoimmune conditions and adverse pregnancy outcomes are causal is important to understand the etiology of these outcomes. Further studies could investigate the biological mechanisms underlying these causal relationships, such as via immune cell traits and cytokine levels, which may identify immune molecular targets to prevent adverse pregnancy outcomes.

## Supplementary Information

Additional file 1. STROBE-MR checklist.

Additional file 2: Supplementary material including Supplementary Figures S1-S56 and Supplementary Methods. Supplementary Figures: FigS1 - Autoimmune condition selection flow diagram. FigS2 - Heatmap of instrument correlations between conditions, including the HLA region. FigS3 - Heatmap of instrument correlations between conditions, excluding the HLA region. FigS4 to FigS6 - Scatter plot of instrument correlations between conditions. FigS7 - Mendelian randomization effect estimates for binary secondary outcomes. FigS8 - Mendelian randomization effect estimates for continuous secondary outcomes. FigS9 to FigS25 - SNP-condition and SNP-outcome scatter plots for Mendelian randomization estimates for results selected for follow up. FigS26 - Pleiotropy-robust methods and HLA excluded Mendelian randomization estimates for results selected for follow up. FigS27 - Steiger filtered Mendelian randomization estimates for results selected for follow up. FigS28 - Weighted linear model Mendelian randomization estimates exploring fetal genetic effects for results selected for follow up. FigS29 to FigS35 - Leave-one-study-out Mendelian randomization estimates for results selected for follow up, by outcome. FigS36 to FigS53 - Leave-one-SNP-out Mendelian randomization estimates for results selected for follow up. FigS54 to FigS58 - Phenome-wide association scans for potentially influential SNPs identified in results selected for follow up. Supplementary methods: Instrument strength. Instrument correlations between conditions. Weighted linear models to explore fetal genetic effects. Sample overlap. Cohort descriptions. Cohort acknowledgements. Cohort funding. Cohort ethical approval.

Additional file 3. Supplementary tables. Table S1 - Characteristics of studies contributing with genetic association data on autoimmune conditions. Table S2A - MR-PREG outcome definitions and sample sizes in GWAS meta-analysis, for all binary outcomes. Table S2B - MR-PREG outcome definitions and sample sizes in GWAS meta-analysis, for all continuous outcomes. Table S3 - Instrument strength for each autoimmune condition. Table S4 - Harmonised SNP-exposure and SNP-outcome data used to conduct Mendelian randomization analysis. Table S5A - Main Mendelian randomization estimates for the effects of increased log odds of autoimmune conditions on binary pregnancy outcomes. Table S5B - Main Mendelian randomization estimates for the effects of increased log odds of autoimmune conditions on continuous pregnancy outcomes. Table S6A - Pleiotropy-robust methods and HLA excluded Mendelian randomization estimates for the effects of increased log odds of autoimmune conditions on binary pregnancy outcomes. Pleiotropy-robust methods and HLA excluded Mendelian randomization estimates for the effects of increased log odds of autoimmune conditions on continuous pregnancy outcomes. Table S7A - Steiger filtered Mendelian randomization estimates for the effects of increased log odds of autoimmune conditions on binary pregnancy outcomes. Table S7B - Steiger filtered Mendelian randomization estimates for the effects of increased log odds of autoimmune conditions on continuous pregnancy outcomes. Table S8A - Weighted linear model Mendelian randomization effect estimates exploring fetal genetic effects on the effects of increased log odds of autoimmune conditions on binary pregnancy outcomes. Table S8B - Weighted linear model Mendelian randomization effect estimates exploring fetal genetic effects on the effects of increased log odds of autoimmune conditions on continuous pregnancy outcomes. Table S9A - Leave-one-study-out and main meta-analysis Mendelian randomization effect estimates for effects of increased log odds of autoimmune conditions on binary pregnancy outcomes. Table S9B - Leave-one-study-out and main meta-analysis Mendelian randomization effect estimates for effects of increased log odds of autoimmune conditions on continuous pregnancy outcomes. Table 10 A - Leave-one-SNP-out Mendelian randomization estimates for the effects of increased log odds of autoimmune conditions on binary pregnancy outcomes. Table S10B - Leave-one-SNP-out Mendelian randomization estimates for the effects of increased log odds of autoimmune conditions on continuous pregnancy outcomes. Table S11 - Metrics for between-SNP heterogeneityand directional pleiotropy. Table S12 - Steiger filtering test for all SNPs used in Mendelian randomization analyses. Table S13A - Sample overlap between autoimmune condition GWAS and MR-PREG outcome GWAS for binary pregnancy outcomes. Table S13B - Sample overlap between autoimmune condition GWAS and MR-PREG outcome GWAS for continuous pregnancy outcomes. Table S14 - Phenome scan results for the potential influential SNPs identified.

## Data Availability

All code and a pre-specified analysis plan (dated 12/02/2024) are available on GitHub at (https://github.com/eaiton/autoimmune-pregnancy). Harmonized SNP association data used for analyses are included in **Additional file 3: Table S4**. To protect participant confidentiality, identifiable supporting data cannot be made openly available. Researchers can apply for access to individual study executive committees. The ALSPAC access policy describes the proposal process in detail including any costs associated with conducting research at ALSPAC, and may be updated from time to time (https://www.bristol.ac.uk/media-library/sites/alspac/documents/researchers/data-access/ALSPAC/_Access/_Policy.pdf). The ALSPAC study website contains details of all the data that is available through a fully searchable data dictionary and variable search tool (http://www.bristol.ac.uk/alspac/researchers/our-data/). Data is available upon request from Born in Bradford (https://borninbradford.nhs.uk/research/how-to-access-data/). Data from MoBa are available from the Norwegian Institute of Public Health after application to the MoBa Scientific Management Group (see its website (https://www.fhi.no/en/ch/studies/moba/for-forskere-artikler/research-and-data-access/) for details). Researchers can apply for access to the UK Biobank data via the Access Management System (AMS) (https://www.ukbiobank.ac.uk/enable-your-research/apply-for-access). FinnGenn genetic summary statistics are freely available online (https://www.finngen.fi/en/access/_results). GWAS Catalogue (https://www.ebi.ac.uk/gwas/home] and IEU Open GWAS (https://gwas.mrcieu.ac.uk/) are publicly available resources.

## Abbreviations

ALSPAC: Avon Longitudinal Study of Parents and Children
BiB: Born in Bradford
GDM: Gestational diabetes mellitus
GWAS: Genome-wide association study
HDP: Hypertensive disorders of pregnancy
HLA: Human leukocyte antigen region
IVW: Inverse-variance weighted
NICU: Neonatal intensive care unit
MoBa: The Norwegian Mother, Father and Child Cohort Study
MR: Mendelian randomization
SNP: Single nucleotide polymorphism
