# Supplementary material for: Characterizing the effects of genetic liability to autoimmune conditions on pregnancy outcomes using Mendelian randomization
Source: BMC Med. 2026 Mar 19;24:272. doi: 10.1186/s12916-026-04797-w (PMC13122933; doi:10.1186/s12916-026-04797-w)
Supplement: Supplementary file 1 — Additional file 1. STROBE-MR checklist. [file 12916_2026_4797_MOESM1_ESM.docx]

**STROBE-MR checklist of recommended items to address in reports of Mendelian randomization studies**^1^ ^2^

| **Item No.** | **Section** | **Checklist item** | **Page No.** | **Relevant text from manuscript** |
| --- | --- | --- | --- | --- |
| 1 | **TITLE and ABSTRACT** | Indicate Mendelian randomization (MR) as the study’s design in the title and/or the abstract if that is a main purpose of the study | 1 | Characterising the effects of genetic liability to autoimmune conditions on pregnancy outcomes using Mendelian Randomization |
|  | **INTRODUCTION** |  |  |  |
| 2 | **Background** | Explain the scientific background and rationale for the reported study. What is the exposure? Is a potential causal relationship between exposure and outcome plausible? Justify why MR is a helpful method to address the study question | 5-7 | Background |
| 3 | **Objectives** | State specific objectives clearly, including pre-specified causal hypotheses (if any). State that MR is a method that, under specific assumptions, intends to estimate causal effects | 6-7 | Final two paragraphs in background |
|  | **METHODS** |  |  |  |
| 4 | **Study design and data sources** | Present key elements of the study design early in the article. Consider including a table listing sources of data for all phases of the study. For each data source contributing to the analysis, describe the following: | 7-8 | Methods  “**Study design**  We used two-sample MR[6] to explore the causal effects of genetic liability to ten autoimmune conditions on nine prespecified primary and seven secondary pregnancy outcomes. Genetic association data for autoimmune conditions were obtained from previous genome-wide association studies (GWAS; n=14,267–520,580). Genetic association data for pregnancy outcomes were obtained from the MR-PREG collaboration (n=74,368–714,889)[15]. **Figure 1** provides an overview of the study design.  **Autoimmune conditions**  We selected autoimmune conditions on the basis that they may be diagnosed in women of reproductive age[1] and that there was at least one published GWAS of the condition with at least 5,000 cases to ensure our MR analyses were well-powered (**Figure S1**). We identified the largest GWAS for each condition on GWAS Catalogue[16] and OpenGWAS[17]. Further details of these GWAS [18–27] can be found in **Table S1**. This identified 10 conditions meeting our criteria (**Figure S1**): ankylosing spondylitis, coeliac disease, Hashimoto’s thyroiditis, inflammatory bowel disease, multiple sclerosis, psoriasis, rheumatoid arthritis, systemic lupus erythematosus, systemic sclerosis, and type 1 diabetes.” |
|  | a) | Setting: Describe the study design and the underlying population, if possible. Describe the setting, locations, and relevant dates, including periods of recruitment, exposure, follow-up, and data collection, when available. | 7-8 | Exposure GWAS: Table S1  Outcome GWAS: “Outcome definitions and sample sizes can be found in Tables S2A-B, and cohort descriptions in Supplementary material.” |
|  | b) | Participants: Give the eligibility criteria, and the sources and methods of selection of participants. Report the sample size, and whether any power or sample size calculations were carried out prior to the main analysis |  | Supplementary Tables 1, 2A and 2B  Supplementary material ‘cohort descriptions’ |
|  | c) | Describe measurement, quality control and selection of genetic variants | 8-9 | Exposure GWAS;  **Instrument selection**  “For each GWAS, we selected genetic variants (single nucleotide polymorphisms; SNPs) strongly associated with the respective autoimmune condition (p-value<5x10^−8^). These were clumped to select independent SNPs (linkage disequilibrium threshold R^2^<0.001, clumping window=10,000 kb) using the 1000 genomes European super population reference panel. For systemic sclerosis, where full GWAS data were not available, we used the independent SNPs identified in the original study using stepwise joint conditional association analyses in 1.5Mb windows around the lead SNP.”  Outcome GWAS:  “Outcome genetic association data were obtained from the MR-PREG collaboration, which integrates data from five cohort studies and four publicly available GWAS[12]. Outcome definitions and sample sizes can be found in Tables S2A-B, and cohort descriptions in Supplementary methods.” |
|  | d) | For each exposure, outcome, and other relevant variables, describe methods of assessment and diagnostic criteria for diseases |  | Supplementary Tables 1, 2A and 2B |
|  | e) | Provide details of ethics committee approval and participant informed consent, if relevant |  | Supplementary methods ‘cohort ethical approval’ |
| 5 | **Assumptions** | Explicitly state the three core IV assumptions for the main analysis (relevance, independence and exclusion restriction) as well assumptions for any additional or sensitivity analysis | 10-11 | Methods: Statistical analysis  “We undertook a series of additional and sensitivity analyses to explore MR assumptions and potential bias in our main analyses…” |
| 6 | **Statistical methods: main analysis** | Describe statistical methods and statistics used | 9-11 | Methods: Statistical analysis |
|  | a) | Describe how quantitative variables were handled in the analyses (i.e., scale, units, model) | 9 | “Our main analyses used the random-effects inverse variance weighted (IVW) estimator. Effect estimates reflect odds ratio (binary outcomes) or differences in mean standard deviation units (continuous outcomes). To aid interpretability effect estimates and standard errors were scaled to reflect a doubling in genetic liability to the autoimmune condition, by multiplying these by log_e_2, before estimating 95% confidence intervals[29]. Given that our binary outcomes are relatively rare, we assume odds and risk of outcomes are interchangeable.” |
|  | b) | Describe how genetic variants were handled in the analyses and, if applicable, how their weights were selected | 9 | “Our main analyses used the random-effects inverse variance weighted (IVW) estimator” |
|  | c) | Describe the MR estimator (e.g. two-stage least squares, Wald ratio) and related statistics. Detail the included covariates and, in case of two-sample MR, whether the same covariate set was used for adjustment in the two samples | 9 | “Our main analyses used the random-effects inverse variance weighted (IVW) estimator” |
|  | d) | Explain how missing data were addressed | 8 | “For systemic sclerosis, where full GWAS data were not available, we used the independent SNPs identified in the original study using stepwise joint conditional association analyses in 1.5Mb windows around the lead SNP.” |
|  | e) | If applicable, indicate how multiple testing was addressed | 9-10 | We identified potential causal effects for follow up with sensitivity analyses if they met either of the following criteria: (i) statistical support at p<0.05, or (ii) relative effect estimates 5% or greater (i.e. odds ratio >1.05 or <0.95) with statistical support at p<0.10. We used these criteria since selecting results based on statistical significance alone is inappropriate in a hypothesis-driven context [30], where we have a strong biological prior on the basis of observational studies, the outcomes of interest are clinically important yet relatively rare, and modest causal effects may still be meaningful. We additionally report whether effects passed multiple testing correction using the Benjamini-Yekutieli false discovery rate (alpha=0.05)[31] to account for arbitrary dependence between tests due to shared genetic risk loci between autoimmune conditions[32] and correlations between pregnancy outcomes. We explored the extent of shared genetic risk by examining instrument correlations between conditions (**Supplementary methods**).” |
| 7 | **Assessment of assumptions** | Describe any methods or prior knowledge used to assess the assumptions or justify their validity | 10-11 | Methods: Statistical analysis  “We undertook a series of additional and sensitivity analyses to explore MR assumptions and potential bias in our main analyses….” |
| 8 | **Sensitivity analyses and additional analyses** | Describe any sensitivity analyses or additional analyses performed (e.g. comparison of effect estimates from different approaches, independent replication, bias analytic techniques, validation of instruments, simulations) | 10-11 | Methods: Statistical analysis  “We undertook a series of additional and sensitivity analyses to explore MR assumptions and potential bias in our main analyses….” |
| 9 | **Software and pre-registration** |  |  |  |
|  | a) | Name statistical software and package(s), including version and settings used | 9 | “Two-sample MR and data harmonisation was conducted using the TwoSampleMR R package[14]. All analyses were conducted in R (4.5.0).” |
|  | b) | State whether the study protocol and details were pre-registered (as well as when and where) | 9 | “All code and a pre-specified analysis plan (dated 12/02/2024) are available at https://github.com/eaiton/autoimmune-pregnancy.” |
|  | **RESULTS** |  |  |  |
| 10 | **Descriptive data** |  |  |  |
|  | a) | Report the numbers of individuals at each stage of included studies and reasons for exclusion. Consider use of a flow diagram |  | Exposure GWAS:  Supplementary Table 1, further details GWAS study papers [18-27]  Outcome GWAS:  Supplementary Tables 2A and 2B, further details in MR-PREG collaboration profile [11] |
|  | b) | Report summary statistics for phenotypic exposure(s), outcome(s), and other relevant variables (e.g. means, SDs, proportions) |  | Exposure GWAS:  Supplementary Table 1, further details GWAS study papers [18-27]  Outcome GWAS:  Supplementary Tables 2A and 2B, further details in MR-PREG collaboration profile [11] |
|  | c) | If the data sources include meta-analyses of previous studies, provide the assessments of heterogeneity across these studies |  | Exposure GWAS:  Supplementary Table 1, further details GWAS study papers [18-27]  Outcome GWAS:  Supplementary Tables 2A and 2B, further details in MR-PREG collaboration profile [11] |
|  | d) | For two-sample MR:  i.  Provide justification of the similarity of the genetic variant-exposure associations between the exposure and outcome samples  ii.  Provide information on the number of individuals who overlap between the exposure and outcome studies | 21  15 | i. Discussion  “Moreover, no autoimmune condition GWAS were stratified by sex, so we could not explore whether potential sex differences in instruments might bias our results. Except ankylosing spondylitis and type 1 diabetes, all conditions analysed here are more common in women which will mitigate this potential source of bias to some extent[1]. Selection bias may also have affected our findings because some autoimmune conditions are associated with reduced fertility[39]. Since our study was restricted to women who have had at least one pregnancy, this could introduce collider bias[51]. We restricted to cohorts of individuals with majority white European ancestry, so further research is needed to assess whether these results are generalisable to non-white European ancestry groups. Sample overlap between GWAS of Hashimoto’s thyroiditis and type 1 diabetes and pregnancy outcome GWAS could bias estimates towards the (confounded) observational estimate [56]. We explored this through leave-one-study-out analyses.”  ii. Results  “There was no sample overlap between most condition and outcome GWAS, except for Hashimoto’s thyroiditis (0-50.6%) and type 1 diabetes (0-38.5%; see **Tables** **S13A-B** for overlap by outcome).” |
| 11 | **Main results** |  |  |  |
|  | a) | Report the associations between genetic variant and exposure, and between genetic variant and outcome, preferably on an interpretable scale | 12 | “Results for all condition-outcome relationships and all sensitivity analyses are provided in **Tables S5-S10,** for effects both per doubling in log odds of autoimmune conditions (as used in main manuscript) and per 1-unit log odds unit increase.” |
|  | b) | Report MR estimates of the relationship between exposure and outcome, and the measures of uncertainty from the MR analysis, on an interpretable scale, such as odds ratio or relative risk per SD difference | 12 | “Results for all condition-outcome relationships and all sensitivity analyses are provided in **Tables S5-S10,** for effects both per doubling in log odds of autoimmune conditions (as used in main manuscript) and per 1-unit log odds unit increase.” |
|  | c) | If relevant, consider translating estimates of relative risk into absolute risk for a meaningful time period |  | NA |
|  | d) | Consider plots to visualize results (e.g. forest plot, scatterplot of associations between genetic variants and outcome versus between genetic variants and exposure) |  | Figure 2 for main analyses, various Supplementary Figures including scatter plots Figure S7-S23 |
| 12 | **Assessment of assumptions** |  |  |  |
|  | a) | Report the assessment of the validity of the assumptions | 13 | Results: Sensitivity analyses |
|  | b) | Report any additional statistics (e.g., assessments of heterogeneity across genetic variants, such as *I^2^*, Q statistic or E-value) |  | Table S11 for heterogeneity, Table S12 for Steiger directionality tests |
| 13 | **Sensitivity analyses and additional analyses** |  |  |  |
|  | a) | Report any sensitivity analyses to assess the robustness of the main results to violations of the assumptions |  | Table 1  Supplementary Tables 5-10 |
|  | b) | Report results from other sensitivity analyses or additional analyses |  | Supplementary Tables 5-10 |
|  | c) | Report any assessment of direction of causal relationship (e.g., bidirectional MR) | 15 | “Steiger tests found that instruments explained more variation in conditions than in outcomes (**Table S12**), except for five ankylosing spondylitis instruments with some outcomes. Steiger filtering did not change any effect estimates except for ankylosing spondylitis where the effect of increased genetic liability on low Apgar score at 5 minutes was less precise as expected given the lower number of instruments available (**Figure S25**).” |
|  | d) | When relevant, report and compare with estimates from non-MR analyses |  | Observational studies cited throughout introduction and discussion |
|  | e) | Consider additional plots to visualize results (e.g., leave-one-out analyses) |  | Supplementary Figures 1-56 |
|  | **DISCUSSION** |  |  |  |
| 14 | **Key results** | Summarize key results with reference to study objectives | 17 | “Our results showed a diverse pattern of effects across conditions and outcomes. For several conditions, increased liability only resulted in higher risk of adverse outcomes (type 1 diabetes, rheumatoid arthritis, systemic lupus erythematosus). For Hashimoto’s thyroiditis, higher genetic liability resulted in both increased and decreased risk of adverse pregnancy outcomes, while higher genetic liability to ankylosing spondylitis reduced risk of HDP only. For other conditions, we found uncertain or unsupportive evidence of causal effects on the outcomes assessed (multiple sclerosis, systemic sclerosis, coeliac disease, inflammatory bowel disease and psoriasis).” |
| 15 | **Limitations** | Discuss limitations of the study, taking into account the validity of the IV assumptions, other sources of potential bias, and imprecision. Discuss both direction and magnitude of any potential bias and any efforts to address them | 20-21 | Discussion paragraphs 10-13 |
| 16 | **Interpretation** |  |  |  |
|  | a) | Meaning: Give a cautious overall interpretation of results in the context of their limitations and in comparison with other studies | 21-22 | Conclusion |
|  | b) | Mechanism: Discuss underlying biological mechanisms that could drive a potential causal relationship between the investigated exposure and the outcome, and whether the gene-environment equivalence assumption is reasonable. Use causal language carefully, clarifying that IV estimates may provide causal effects only under certain assumptions | 18-19 | Discussion paragraphs 6-8 |
|  | c) | Clinical relevance: Discuss whether the results have clinical or public policy relevance, and to what extent they inform effect sizes of possible interventions | 21-22 | Conclusions |
| 17 | **Generalizability** | Discuss the generalizability of the study results (a) to other populations, (b) across other exposure periods/timings, and (c) across other levels of exposure | 19-20 | Discussion paragraph 13 |
|  | **OTHER INFORMATION** |  |  |  |
| 18 | **Funding** | Describe sources of funding and the role of funders in the present study and, if applicable, sources of funding for the databases and original study or studies on which the present study is based | 26-27 | Funding |
| 19 | **Data and data sharing** | Provide the data used to perform all analyses or report where and how the data can be accessed, and reference these sources in the article. Provide the statistical code needed to reproduce the results in the article, or report whether the code is publicly accessible and if so, where | 25-26 | Availability of data and materials |
| 20 | **Conflicts of Interest** | All authors should declare all potential conflicts of interest | 26 | “All authors declare no competing interests.” |

This checklist is copyrighted by the Equator Network under the Creative Commons Attribution 3.0 Unported (CC BY 3.0) license.

1. Skrivankova VW, Richmond RC, Woolf BAR, Yarmolinsky J, Davies NM, Swanson SA, et al. Strengthening the Reporting of Observational Studies in Epidemiology using Mendelian Randomization (STROBE-MR) Statement. JAMA. 2021;under review.

2. Skrivankova VW, Richmond RC, Woolf BAR, Davies NM, Swanson SA, VanderWeele TJ, et al. Strengthening the Reporting of Observational Studies in Epidemiology using Mendelian Randomisation (STROBE-MR): Explanation and Elaboration. BMJ. 2021;375:n2233.
