# Supplementary material for: Characterizing the effects of genetic liability to autoimmune conditions on pregnancy outcomes using Mendelian randomization
Source: BMC Med. 2026 Mar 19;24:272. doi: 10.1186/s12916-026-04797-w (PMC13122933; doi:10.1186/s12916-026-04797-w)
Supplement: Supplementary file 2 — Additional file 2: Supplementary material including Supplementary Figures S1-S56 and Supplementary Methods. Supplementary Figures: FigS1 - Autoimmune condition selection flow diagram. FigS2 - Heatmap of instrument correlations between conditions, including the HLA region. FigS3 - Heatmap of instrument correlations between conditions, excluding the HLA region. FigS4 to FigS6 - Scatter plot of instrument correlations between conditions. FigS7 - Mendelian randomization effect estimates for binary secondary outcomes. FigS8 - Mendelian randomization effect estimates for continuous secondary outcomes. FigS9 to FigS25 - SNP-condition and SNP-outcome scatter plots for Mendelian randomization estimates for results selected for follow up. FigS26 - Pleiotropy-robust methods and HLA excluded Mendelian randomization estimates for results selected for follow up. FigS27 - Steiger filtered Mendelian randomization estimates for results selected for follow up. FigS28 - Weighted linear model Mendelian randomization estimates exploring fetal genetic effects for results selected for follow up. FigS29 to FigS35 - Leave-one-study-out Mendelian randomization estimates for results selected for follow up, by outcome. FigS36 to FigS53 - Leave-one-SNP-out Mendelian randomization estimates for results selected for follow up. FigS54 to FigS58 - Phenome-wide association scans for potentially influential SNPs identified in results selected for follow up. Supplementary methods: Instrument strength. Instrument correlations between conditions. Weighted linear models to explore fetal genetic effects. Sample overlap. Cohort descriptions. Cohort acknowledgements. Cohort funding. Cohort ethical approval. [file 12916_2026_4797_MOESM2_ESM.docx]

**Supplementary Material**

Table of Contents

[Supplementary Figures 2](#_Toc223959401)

[Autoimmune condition selection 2](#_Toc223959402)

[Instrument correlations between conditions 3](#_Toc223959403)

[Secondary outcomes 8](#_Toc223959404)

[Scatter plots 10](#_Toc223959405)

[Pleiotropy-robust MR methods and excluding HLA variants 27](#_Toc223959406)

[Steiger filtering 29](#_Toc223959407)

[Fetal genetic effects 30](#_Toc223959408)

[Leave one study out 31](#_Toc223959409)

[Leave one SNP out estimates 34](#_Toc223959410)

[Phenome wide scan for influential SNPs 52](#_Toc223959411)

[Supplementary Methods 55](#_Toc223959412)

[Instrument strength 55](#_Toc223959413)

[Instrument correlations between conditions 56](#_Toc223959414)

[Weighted linear models to explore fetal genetic effects 57](#_Toc223959415)

[Sample overlap 58](#_Toc223959416)

[Cohort descriptions 59](#_Toc223959417)

[Avon Longitudinal Study of Parents and Children (ALSPAC) 59](#_Toc223959418)

[Born in Bradford (BiB) 59](#_Toc223959419)

[Norwegian Mother, Father and Child Cohort Study (MoBa) 59](#_Toc223959420)

[FinnGen 59](#_Toc223959421)

[UK Biobank (UKB) 59](#_Toc223959422)

[Cohort acknowledgments 60](#_Toc223959423)

[Avon Longitudinal Study of Parents and Children (ALSPAC) 60](#_Toc223959424)

[Born in Bradford (BiB) 60](#_Toc223959425)

[Norwegian Mother, Father and Child Cohort Study (MoBa) 60](#_Toc223959426)

[FinnGen 60](#_Toc223959427)

[UK Biobank (UKB) 60](#_Toc223959428)

[Cohort funding 61](#_Toc223959429)

[Avon Longitudinal Study of Parents and Children (ALSPAC) 61](#_Toc223959430)

[Born in Bradford (BiB) 61](#_Toc223959431)

[Norwegian Mother, Father and Child Cohort Study (MoBa) 61](#_Toc223959432)

[UK Biobank (UKB) 61](#_Toc223959433)

[Cohort ethical approval 62](#_Toc223959434)

[Avon Longitudinal Study of Parents and Children (ALSPAC) 62](#_Toc223959435)

[Born in Bradford (BiB) 62](#_Toc223959436)

[Norwegian Mother, Father and Child Cohort Study (MoBa) 62](#_Toc223959437)

[UK Biobank (UKB) 62](#_Toc223959438)

# Supplementary Figures

## Autoimmune condition selection


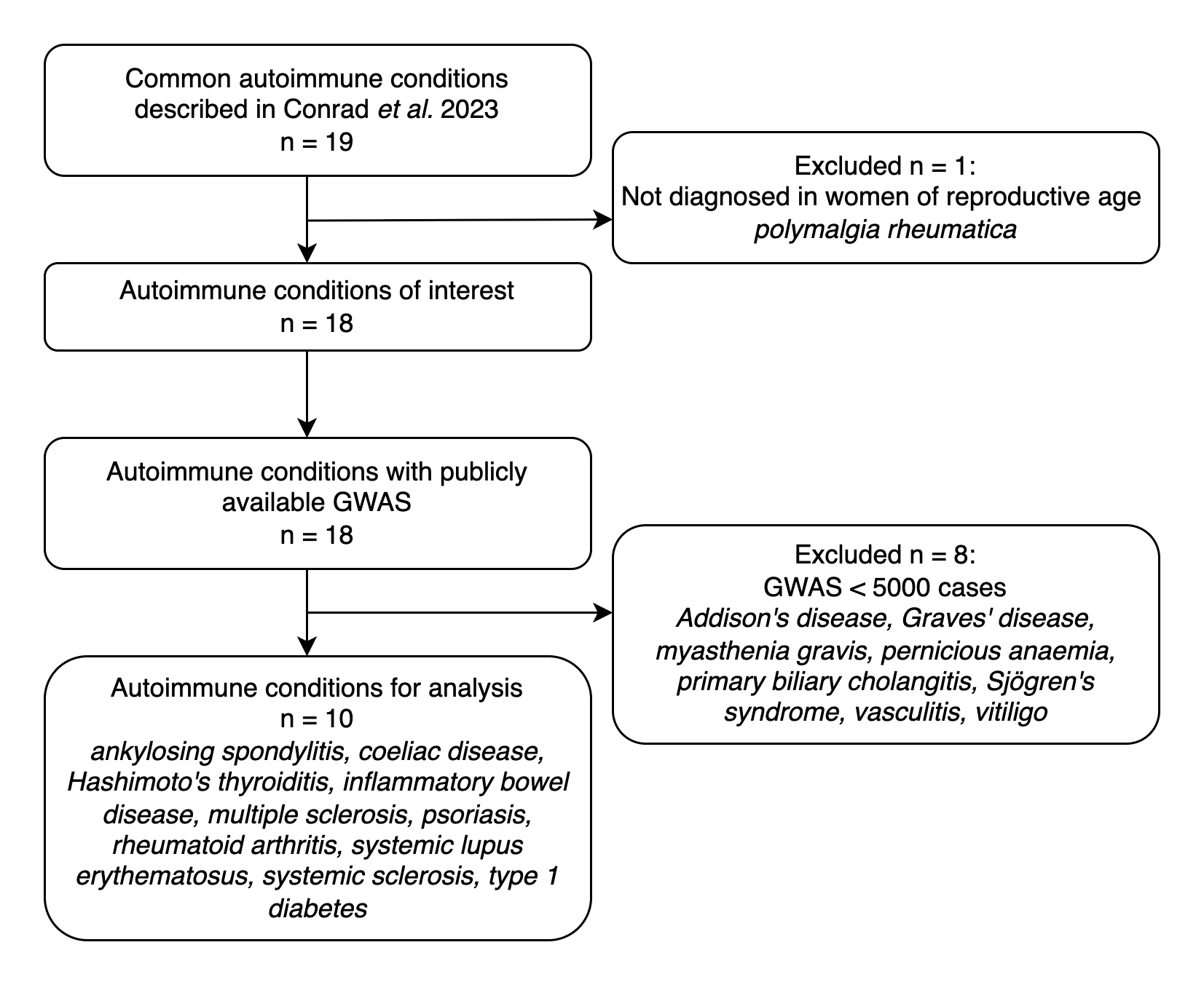


Figure 1. Flow diagram explaining selection of autoimmune conditions for analysis. GWAS = genome-wide association study.

## Instrument correlations between conditions


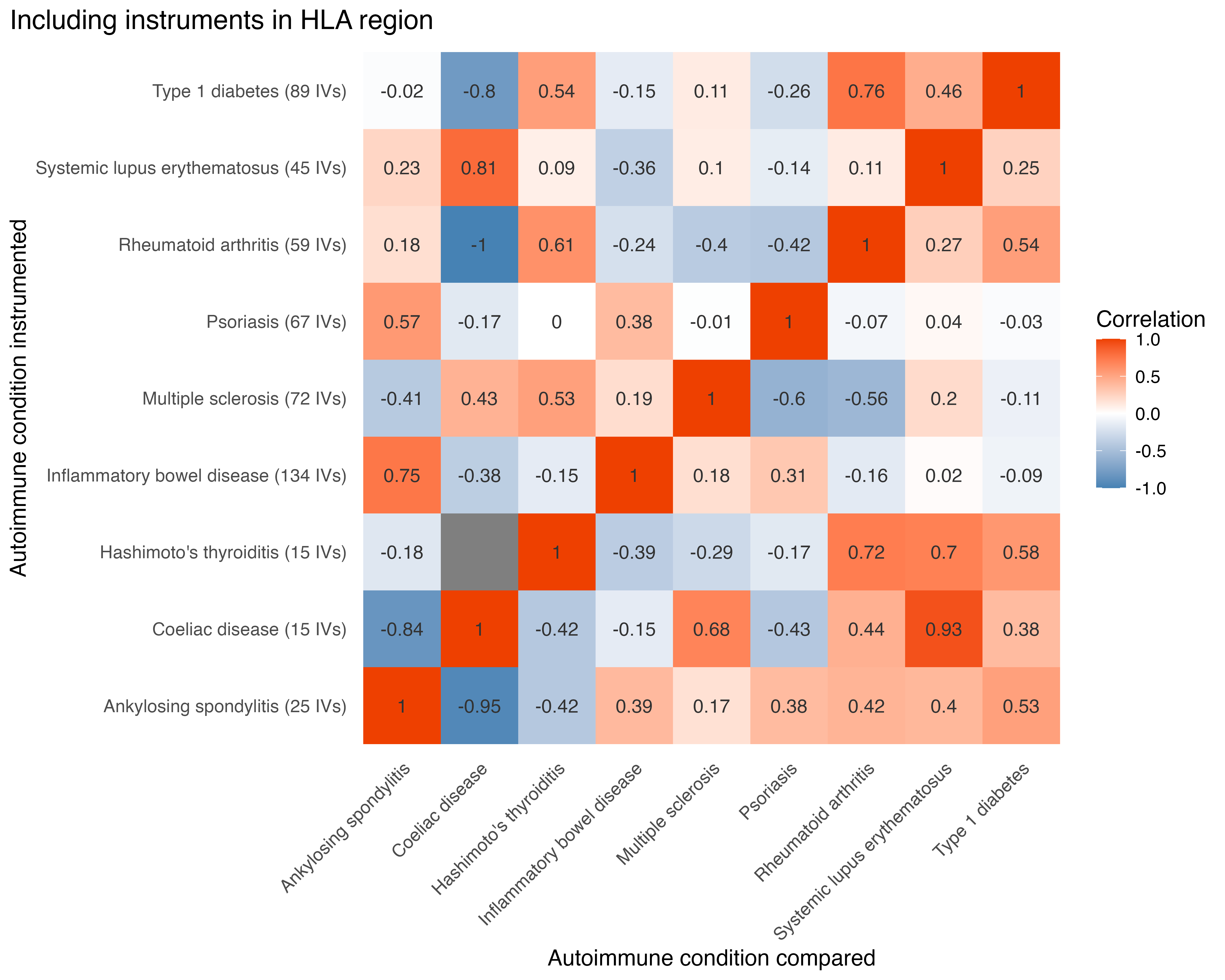


Figure 2. Heatmap of Pearson correlations between SNP-condition betas (log odds units) for the genetic instruments for each condition instrumented and other autoimmune conditions considered, including genetic instruments in the HLA region. Number of SNPs available varies between condition GWAS, and grey squares indicate instances where 2 or fewer SNPs were available for comparison so correlation was not estimated. The GWAS for coeliac disease was conducting using a custom array without genome-wide coverage so there is a high degree of missingness in this condition, for instance 4/59 rheumatoid arthritis instruments were available in this GWAS here. Full summary statistics were not made available for systemic sclerosis so this condition was not included. Underlying scatter plots for all SNPs in each comparison are presented below in Figure 4. HLA = human leukocyte antigen.


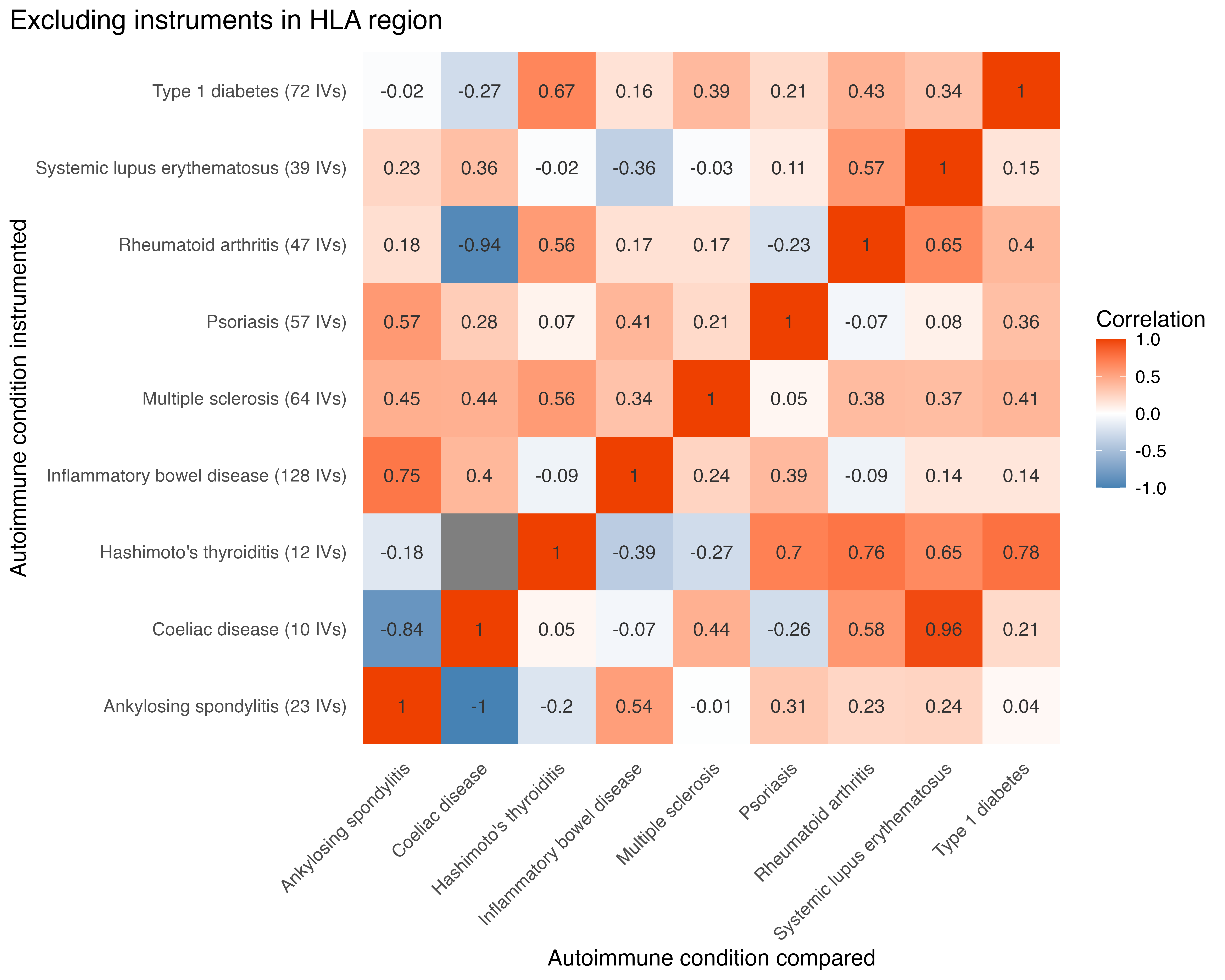


Figure 3. Heatmap of Pearson correlations between SNP-condition betas (log odds units) for the genetic instruments for each condition instrumented and other autoimmune conditions considered, excluding instruments in the HLA region. Number of SNPs available varies between condition GWAS, and grey squares indicate instances where 2 or fewer SNPs were available for comparison so correlation was not estimated. The GWAS for coeliac disease was conducting using a custom array without genome-wide coverage so there is a high degree of missingness in this condition, for instance 3/47 rheumatoid arthritis instruments were available here. Full summary statistics were not made available for systemic sclerosis so this condition was not included. HLA = human leukocyte antigen.


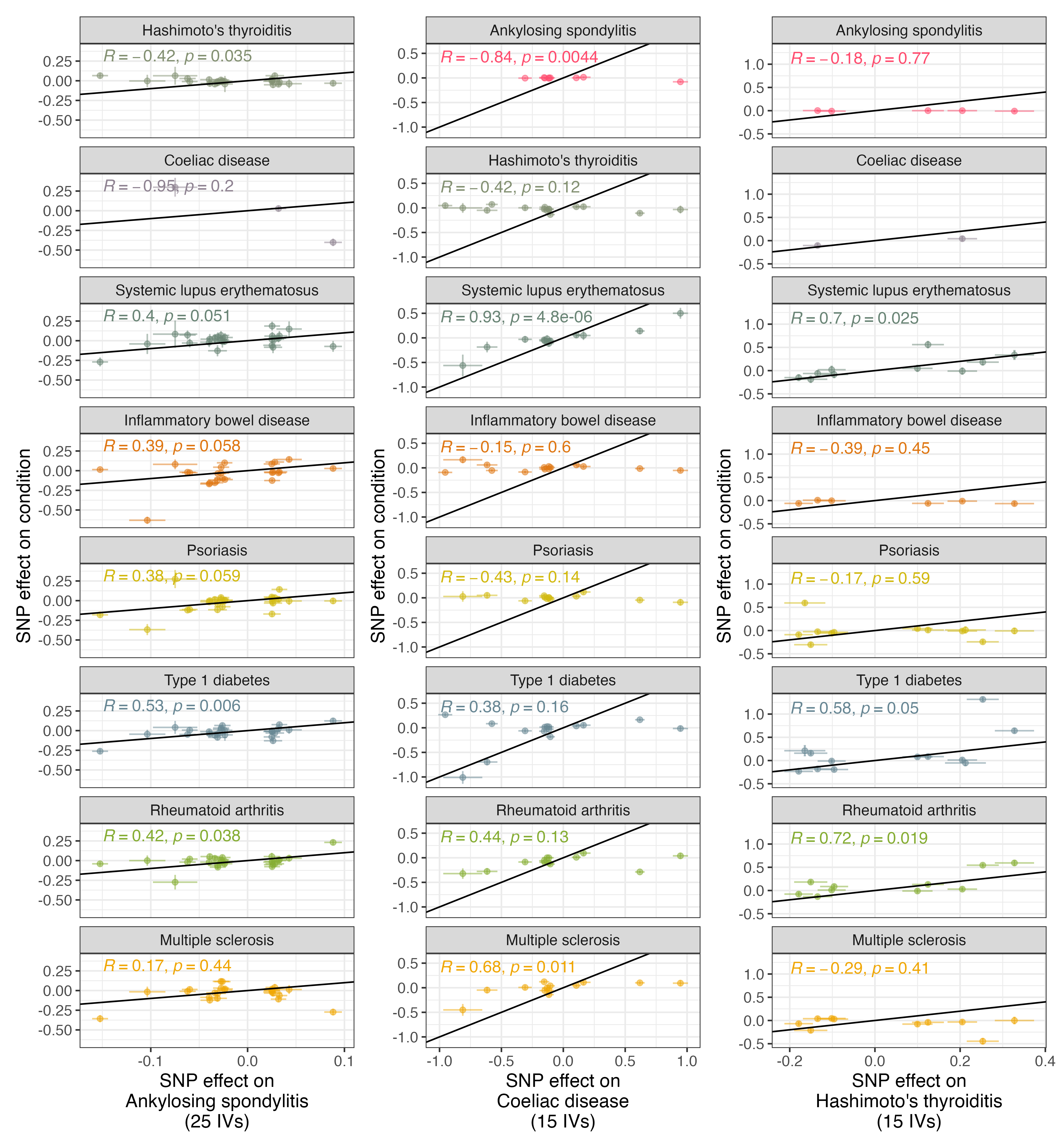


Figure 4. Scatter plots illustrating the SNP-condition betas (log odds units) for the genetic instruments for ankylosing spondylitis, coeliac disease and Hashimoto’s thyroiditis against other autoimmune conditions considered, including the genetic instruments in the human leukocyte antigen (HLA) region. Pearson’s correlation coefficient and uncorrected P-value are displayed for each comparison. Error bars represent 95% confidence intervals around estimated SNP-condition betas. Number of SNPs available varies between condition GWAS, and in instances where 2 or fewer SNPs were available for comparison correlation was not estimated. The GWAS for coeliac disease was conducting using a custom array without genome-wide coverage so there is a high degree of missingness in this condition, for instance 2/15 Hashimoto’s thyroiditis instruments were available here. Full summary statistics were not made available for systemic sclerosis so this condition was not included. An overview of the correlations across all conditions is presented as a heatmap above in Figure 2; correlations excluding instruments located in the HLA region are presented in above in Figure 3. IVs = instrumental variables.


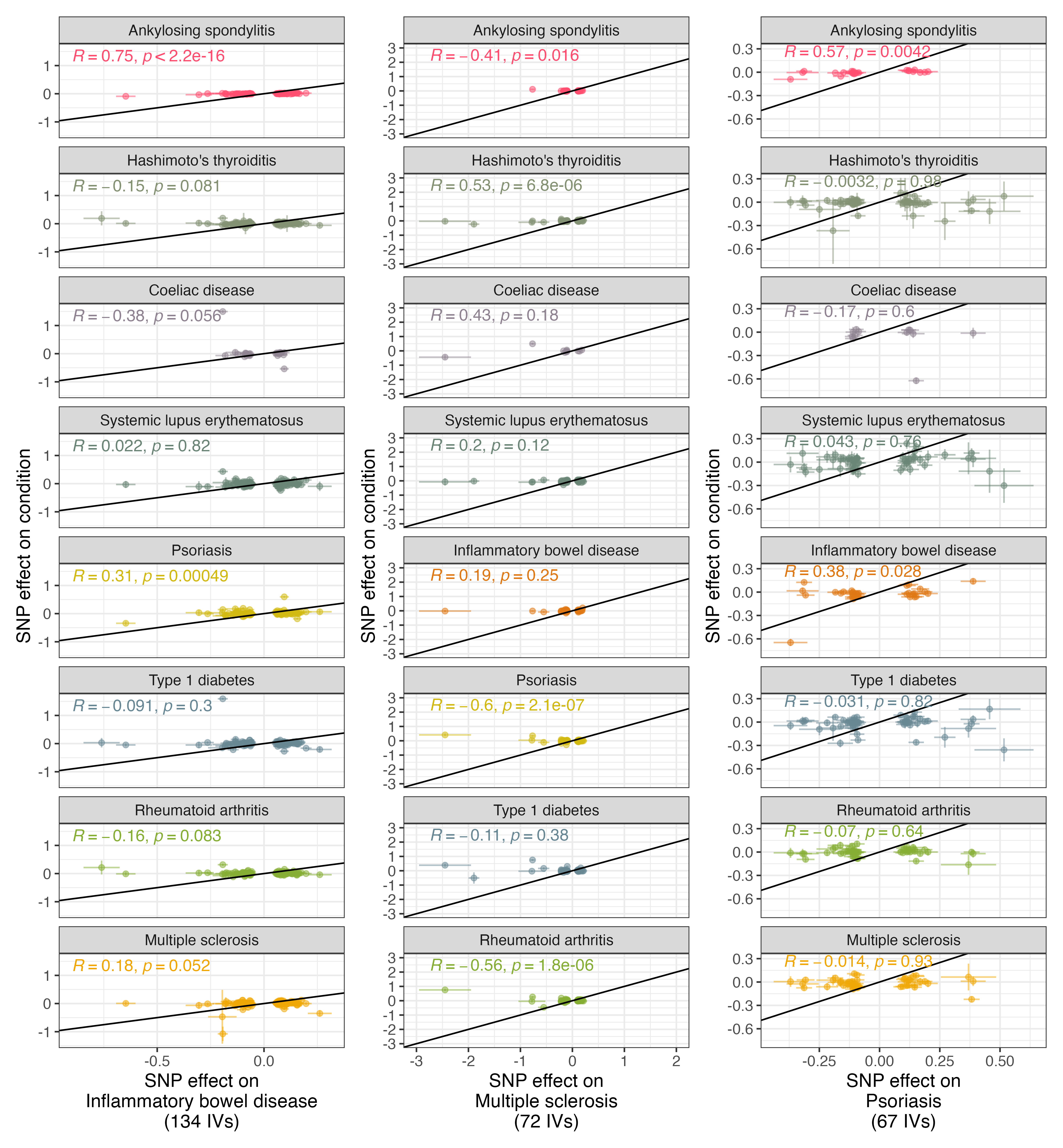


Figure 5. Scatter plots illustrating the SNP-condition betas (log odds units) for the genetic instruments for inflammatory bowel disease, multiple sclerosis and psoraises against other autoimmune conditions considered, including the genetic instruments in the human leukocyte antigen (HLA) region. Pearson’s correlation coefficient and uncorrected P-value are displayed for each comparison. Error bars represent 95% confidence intervals around estimated SNP-condition betas. Number of SNPs available varies between condition GWAS, and in instances where 2 or fewer SNPs were available for comparison correlation was not estimated. The GWAS for coeliac disease was conducting using a custom array without genome-wide coverage so there is a high degree of missingness in this condition. Full summary statistics were not made available for systemic sclerosis so this condition was not included. An overview of the correlations across all conditions is presented as a heatmap above in Figure 2; correlations excluding instruments located in the HLA region are presented in above in Figure 3. IVs = instrumental variables.


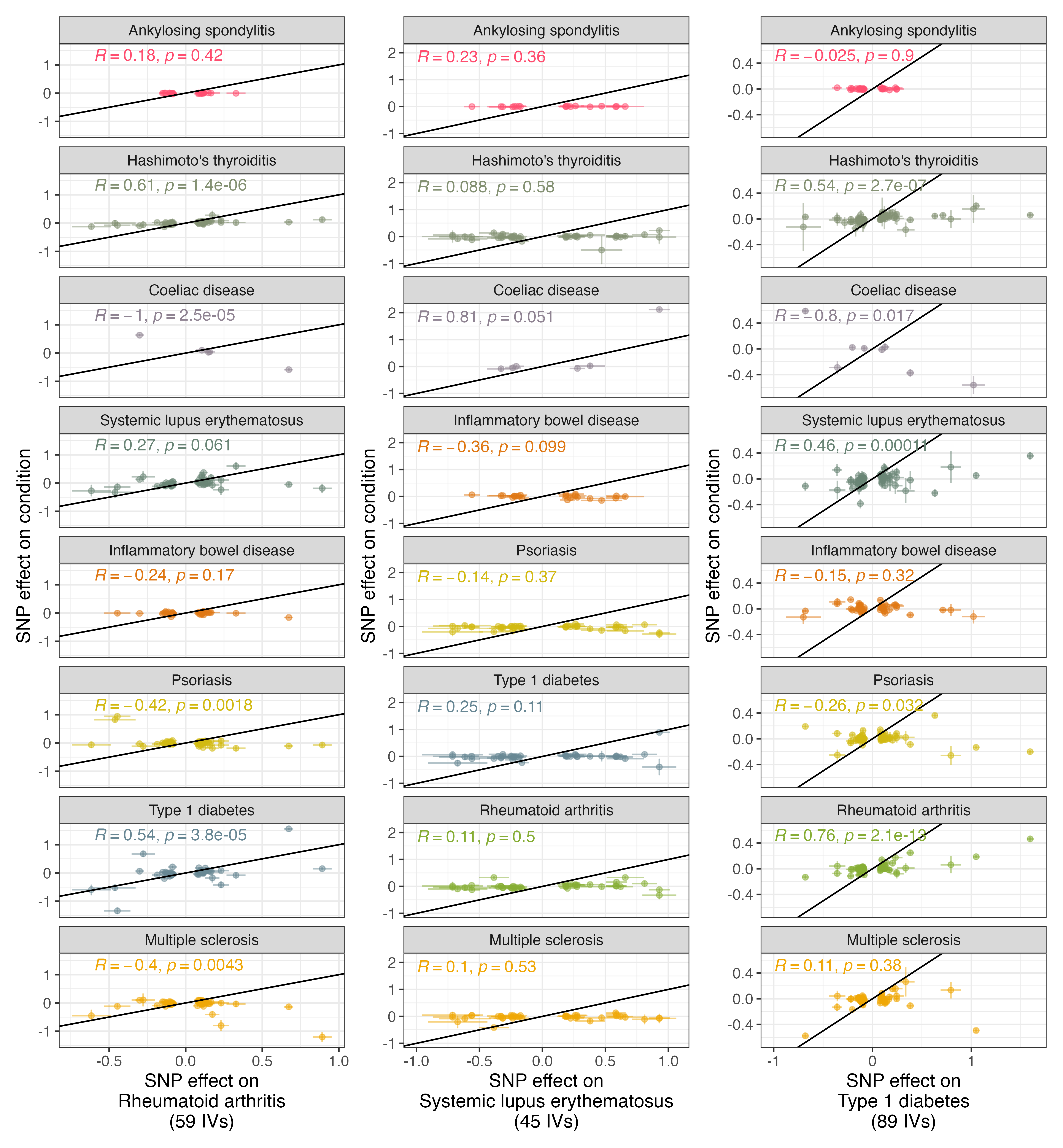


Figure 6. Scatter plots illustrating the SNP-condition betas (log odds units) for the genetic instruments for rheumatoid arthritis, systemic lupus erythematosus, and type 1 diabetes against other autoimmune conditions considered, including the genetic instruments in the human leukocyte antigen (HLA) region. Pearson’s correlation coefficient and uncorrected P-value are displayed for each comparison. Error bars represent 95% confidence intervals around estimated SNP-condition betas. Number of SNPs available varies between condition GWAS, and in instances where 2 or fewer SNPs were available for comparison correlation was not estimated. The GWAS for coeliac disease was conducting using a custom array without genome-wide coverage so there is a high degree of missingness in this condition, for instance 4/59 rheumatoid arthritis instruments were available here. Full summary statistics were not made available for systemic sclerosis so this condition was not included. An overview of the correlations across all conditions is presented as a heatmap above in Figure 2; correlations excluding instruments located in the HLA region are presented in above in Figure 3. IVs = instrumental variables.

## Secondary outcomes


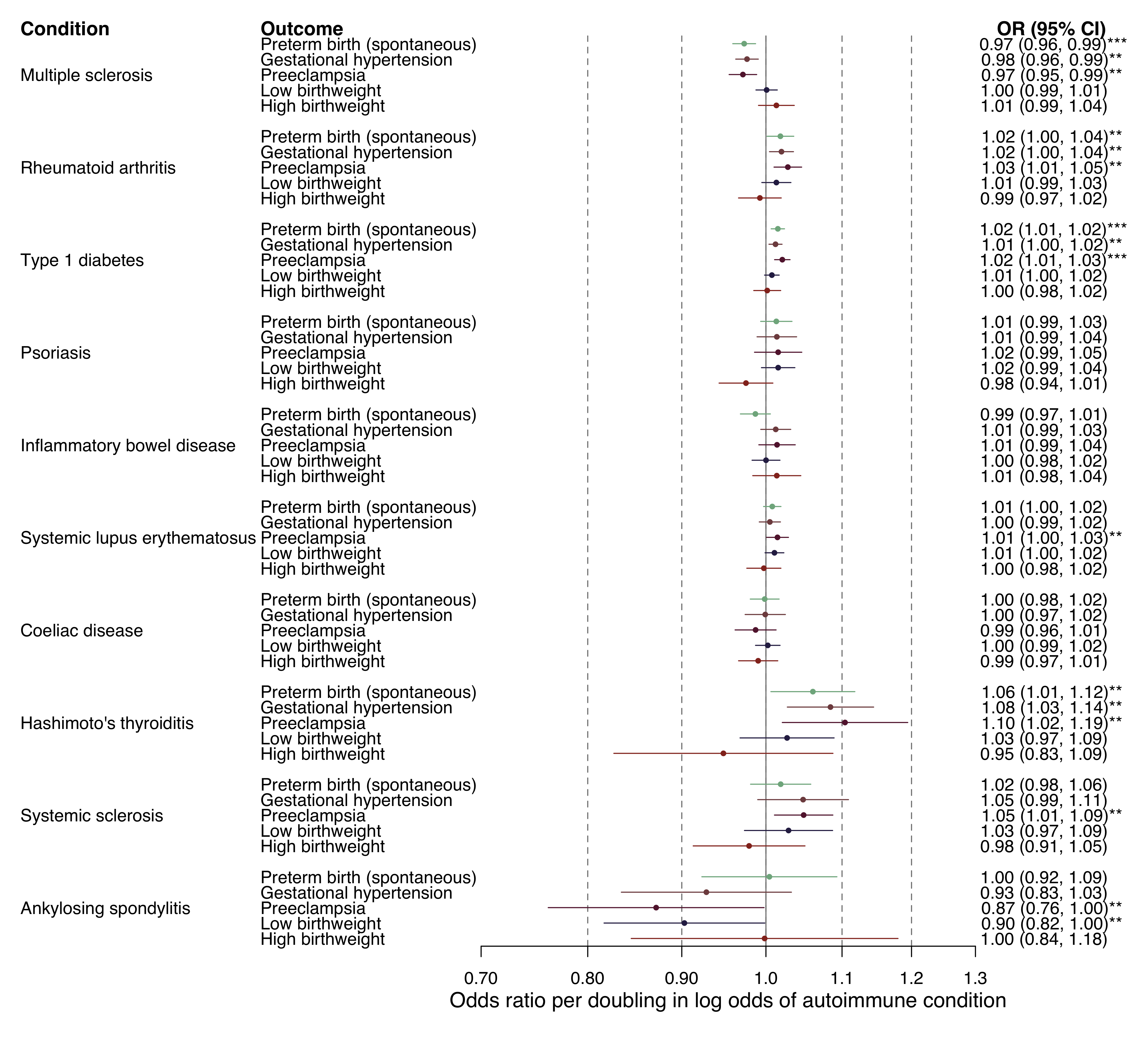


Figure 7. Mendelian randomization estimates for the effect of log odds of autoimmune conditions on binary secondary pregnancy outcomes. Estimates reflect odds ratio of pregnancy outcome per doubling in log odds of autoimmune condition. * indicates p<0.1 and change in odds of 5% or more, ** indicates p<0.05, *** indicates p<0.05 after correcting for multiple testing using false discovery rate.


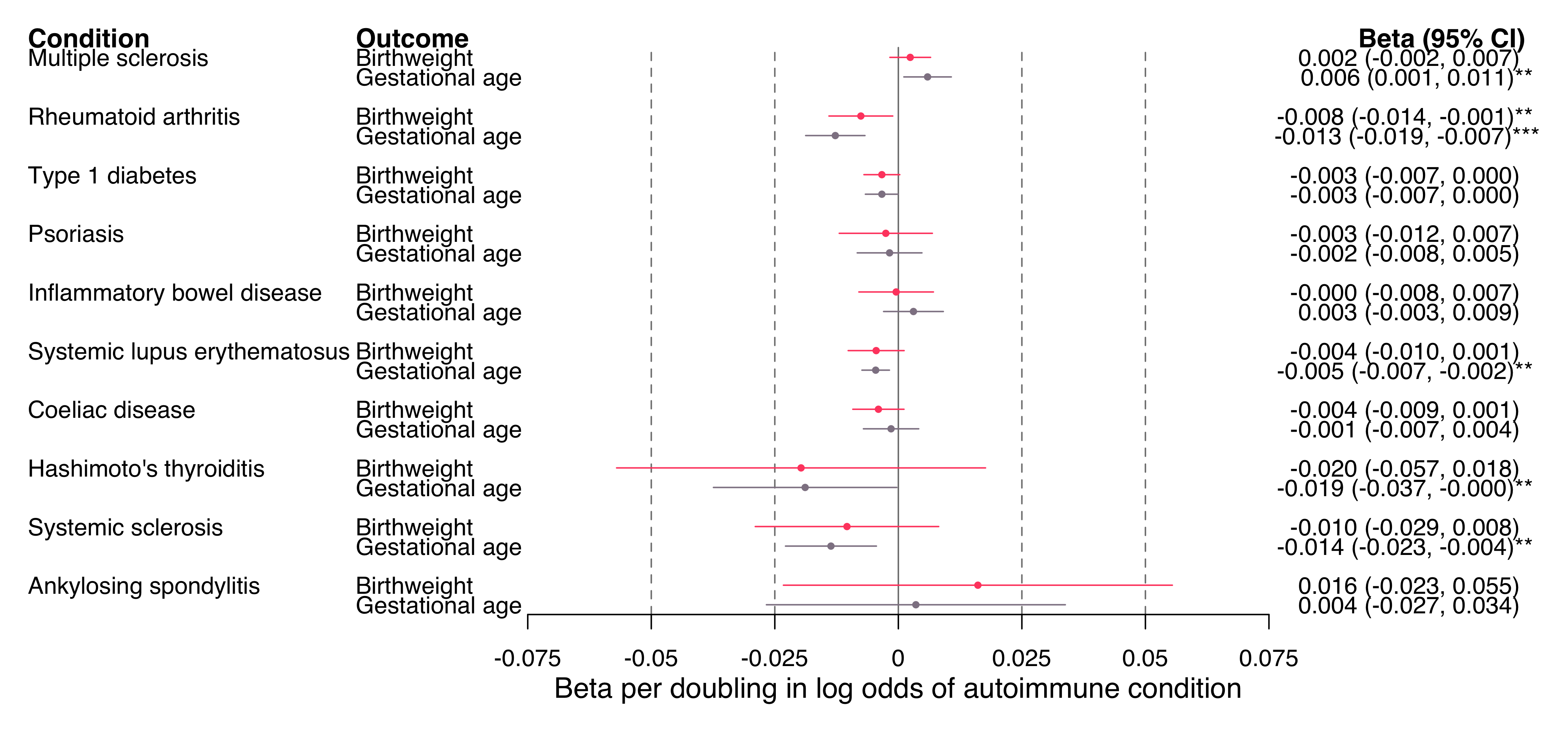


Figure 8. Mendelian randomization estimates for the effect of log odds of autoimmune conditions on continuous secondary pregnancy outcomes. Estimates reflect mean difference in outcome (in standard deviation units) per doubling in log odds of autoimmune conditions. One SD birthweight is ~601.9 grams and one SD of gestational age is 1.8 weeks or 12.6 days. ** indicates p<0.05, *** indicates p < 0.05 after correcting for multiple testing using false discovery rate.

## Scatter plots


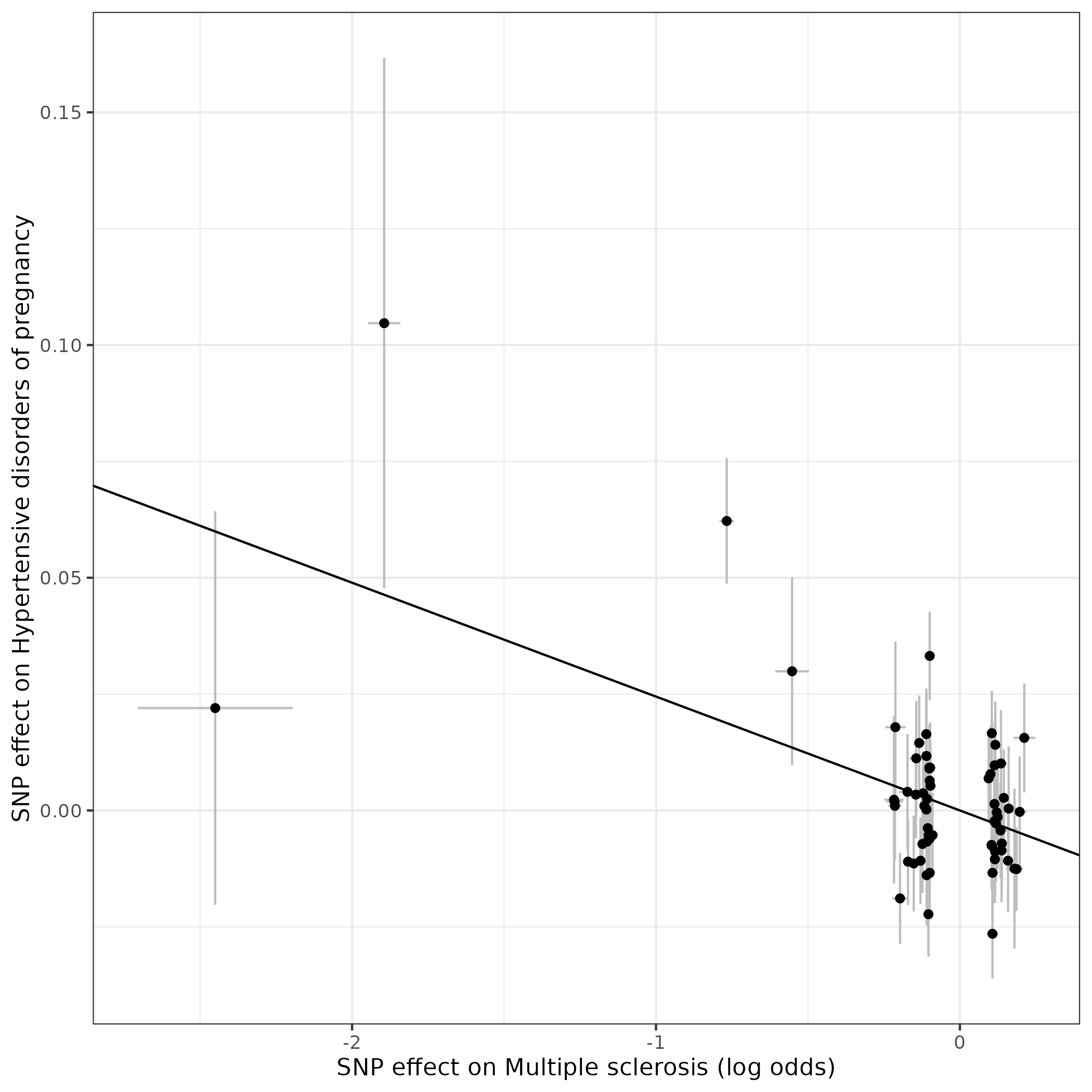


Figure 9. SNP-condition and SNP-outcome scatterplots for the effect of multiple sclerosis genetic liability (log odds) on hypertensive disorders of pregnancy (log odds). Error bars indicate one standard error either side of beta point estimate, black line illustrates the inverse variance weighted estimator.


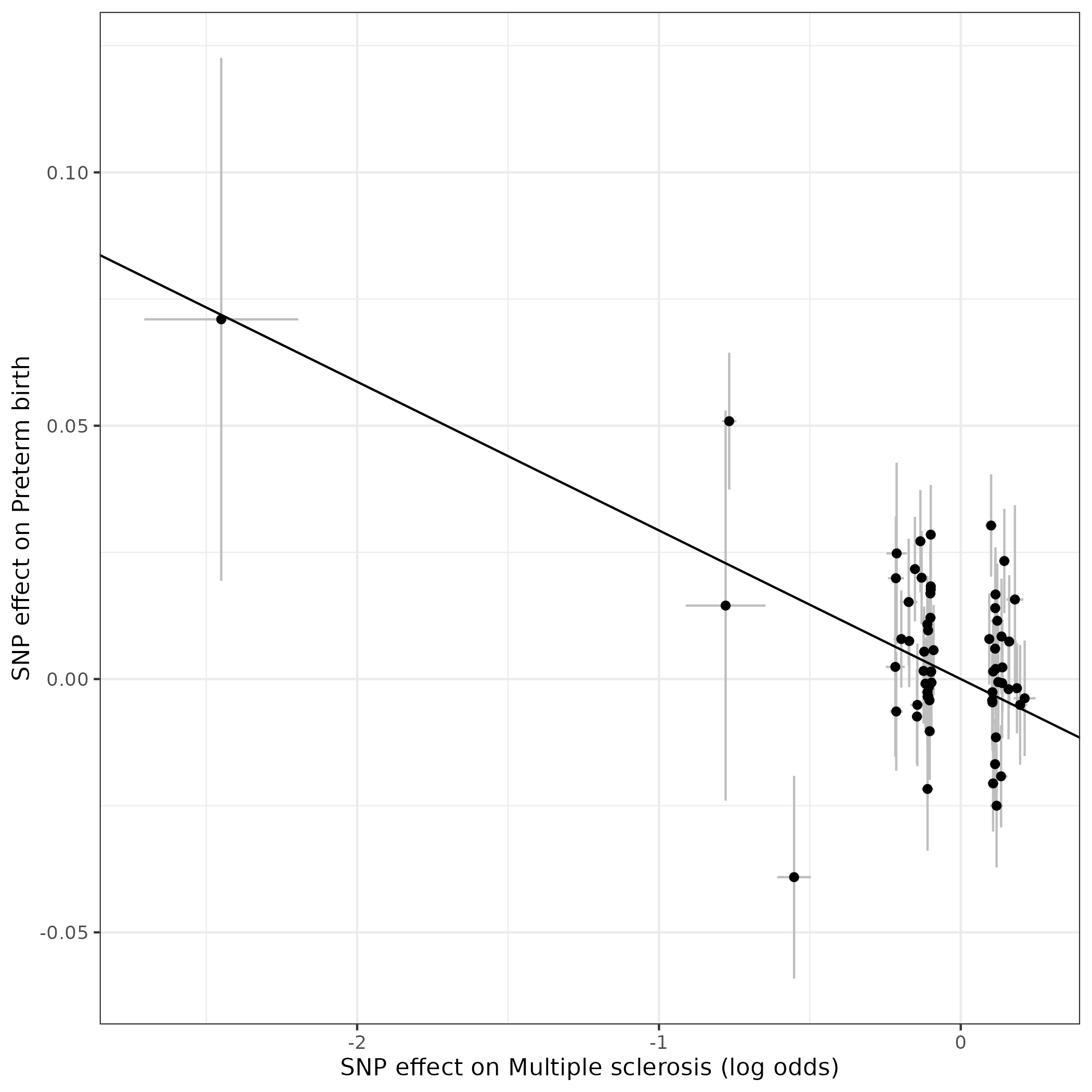


Figure 10. SNP-condition and SNP-outcome scatterplots for the effect of multiple sclerosis genetic liability (log odds) on preterm birth (log odds). Error bars indicate one standard error either side of beta point estimate, black line illustrates the inverse variance weighted estimator.


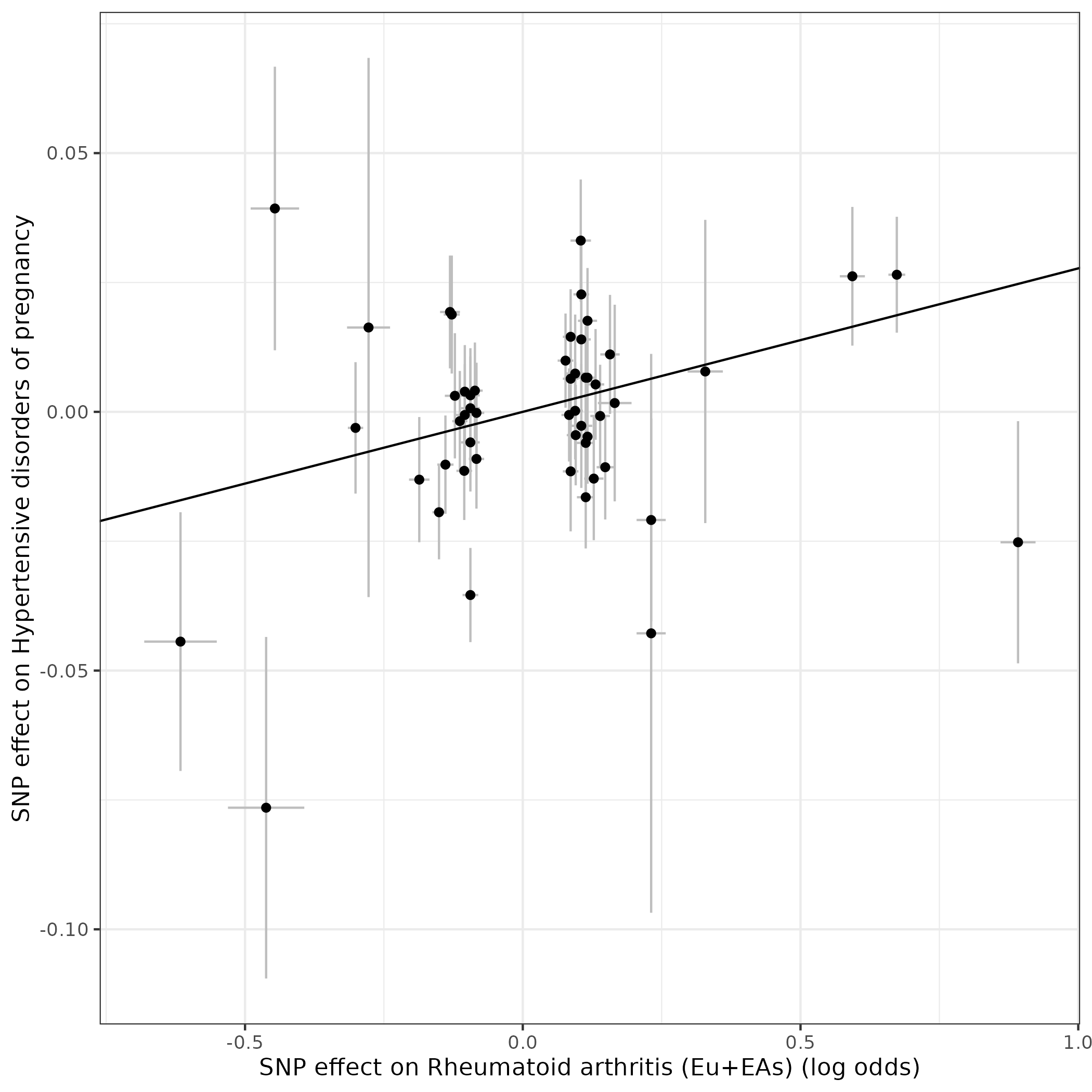


Figure 11. SNP-condition and SNP-outcome scatterplots for the effect of rheumatoid arthritis genetic liability (log odds) on hypertensive disorders of pregnancy (log odds). Error bars indicate one standard error either side of beta point estimate, black line illustrates the inverse variance weighted estimator.


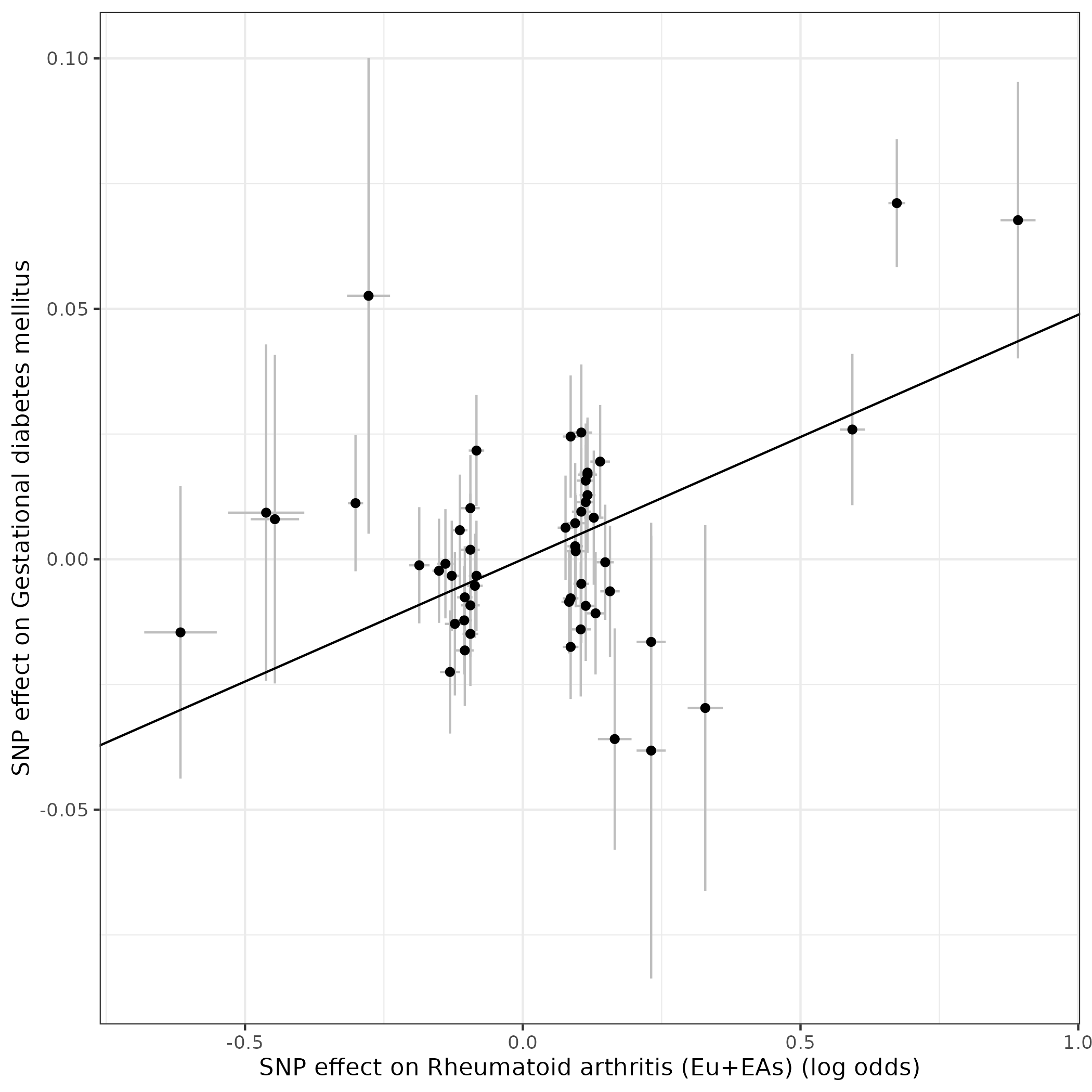


Figure 12. SNP-condition and SNP-outcome scatterplots for the effect of rheumatoid genetic liability (log odds) on gestational diabetes mellitus (log odds). Error bars indicate one standard error either side of beta point estimate, black line illustrates the inverse variance weighted estimator.


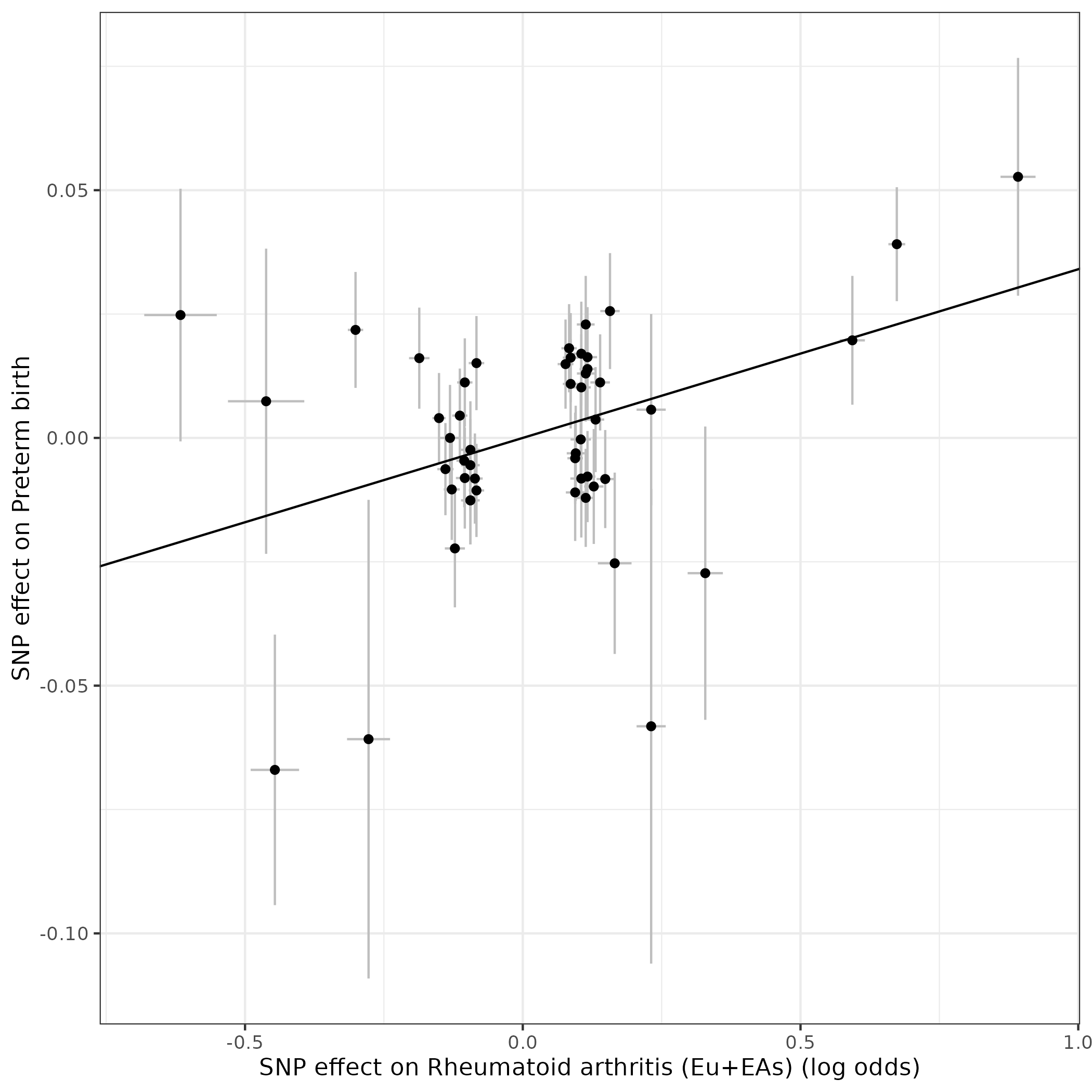


Figure 13. SNP-condition and SNP-outcome scatterplots for the effect of rheumatoid arthritis genetic liability (log odds) on preterm birth (log odds). Error bars indicate one standard error either side of beta point estimate, black line illustrates the inverse variance weighted estimator.


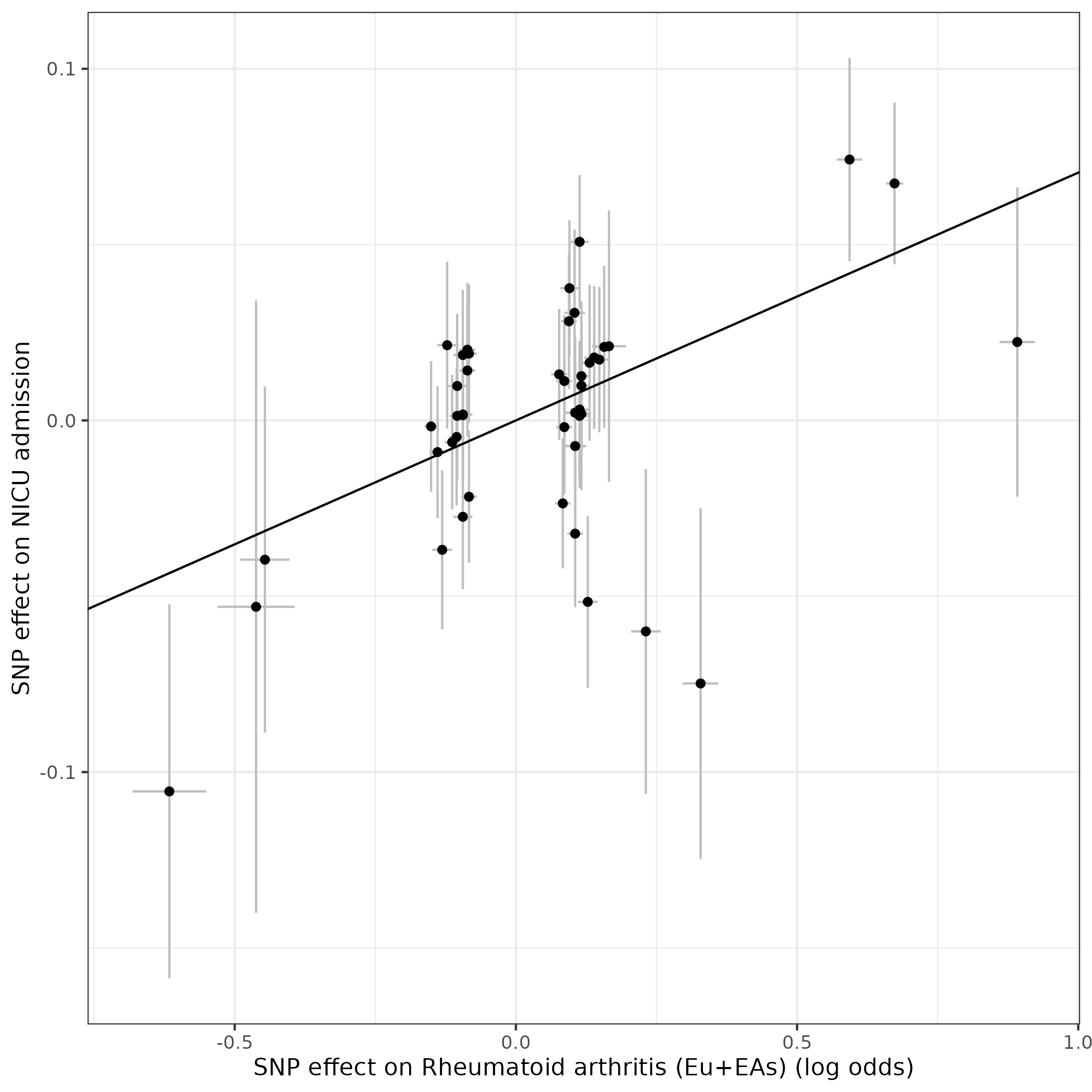


Figure 14. SNP-condition and SNP-outcome scatterplots for the effect of rheumatoid arthritis genetic liability (log odds) on neonatal intensive care unit (NICU) admission (log odds). Error bars indicate one standard error either side of beta point estimate, black line illustrates the inverse variance weighted estimator.


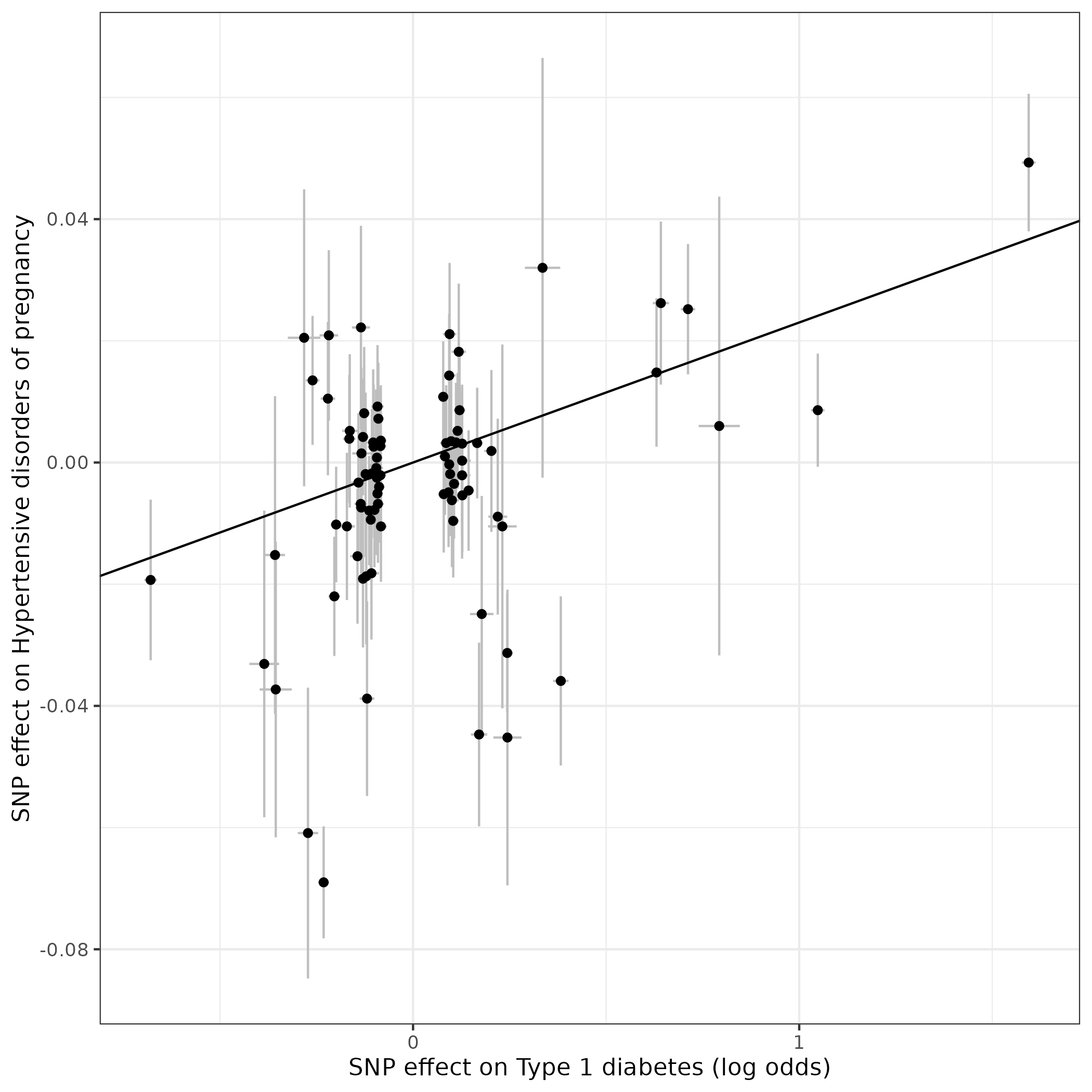


Figure 15. SNP-condition and SNP-outcome scatterplots for the effect of type 1 diabetes genetic liability (log odds) on hypertensive disorders of pregnancy (log odds). Error bars indicate one standard error either side of beta point estimate, black line illustrates the inverse variance weighted estimator.


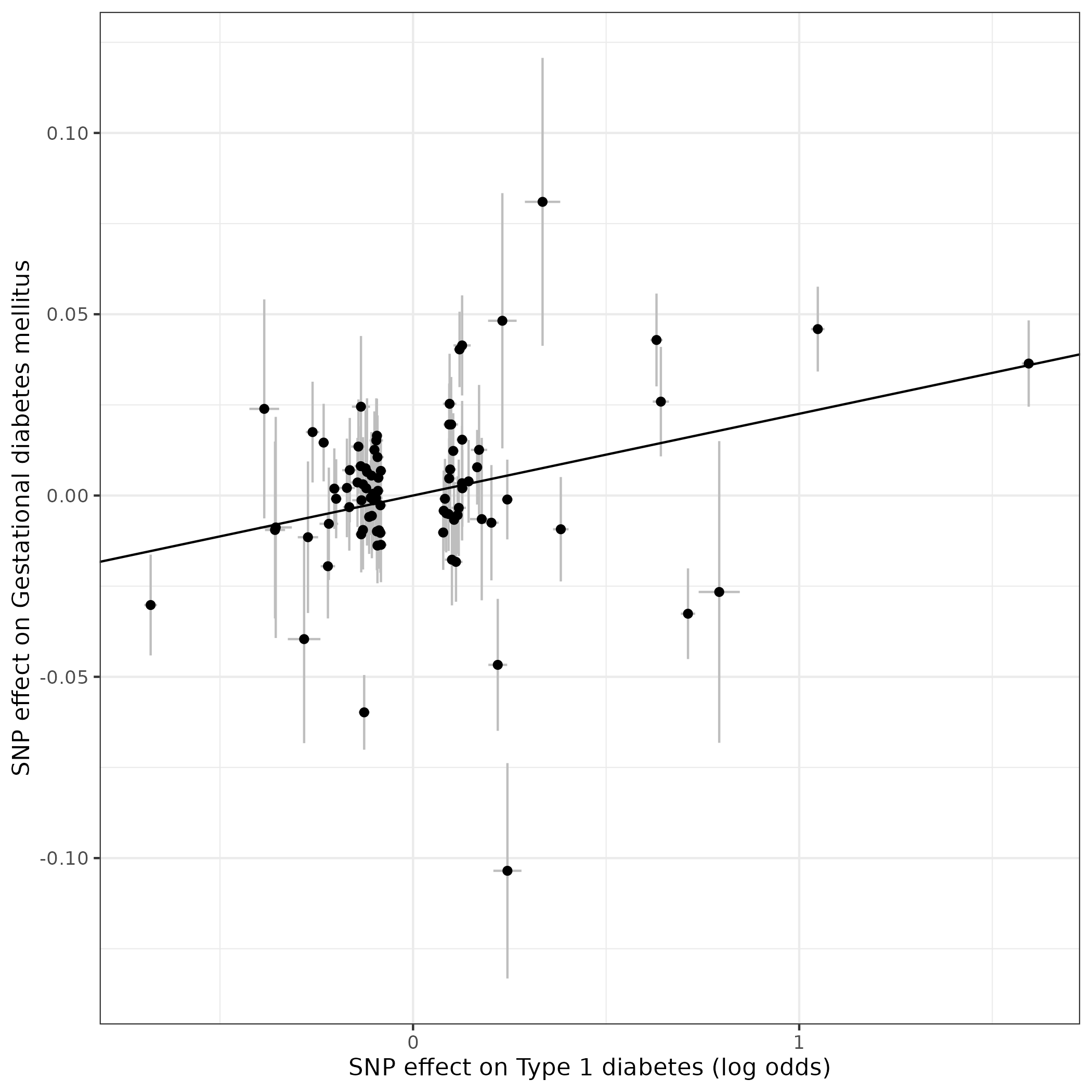


Figure 16. SNP-condition and SNP-outcome scatterplots for the effect of type 1 diabetes genetic liability (log odds) on gestational diabetes mellitus (log odds). Error bars indicate one standard error either side of beta point estimate, black line illustrates the inverse variance weighted estimator.


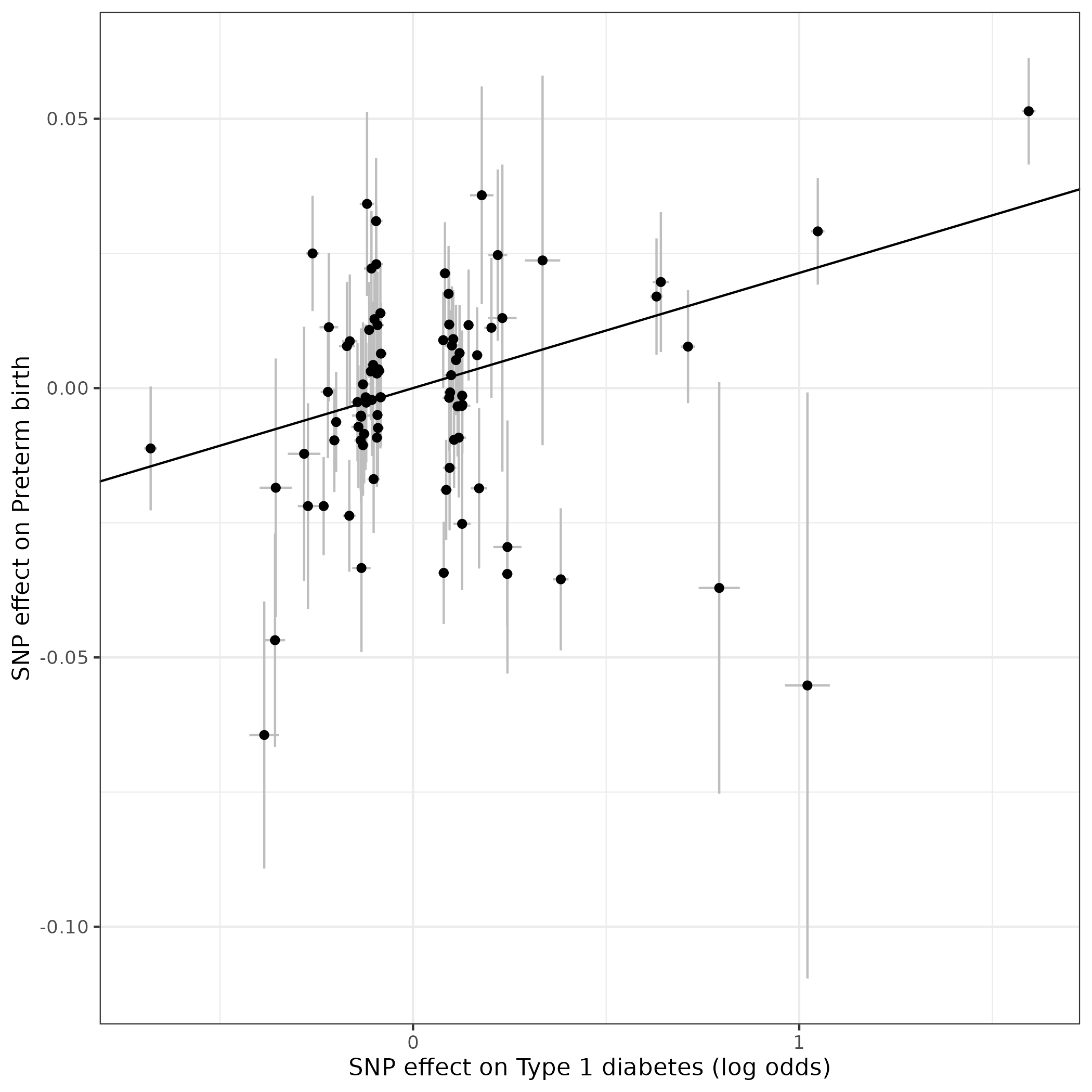


Figure 17. SNP-condition and SNP-outcome scatterplots for the effect of type 1 diabetes genetic liability (log odds) on preterm birth (log odds). Error bars indicate one standard error either side of beta point estimate, black line illustrates the inverse variance weighted estimator.


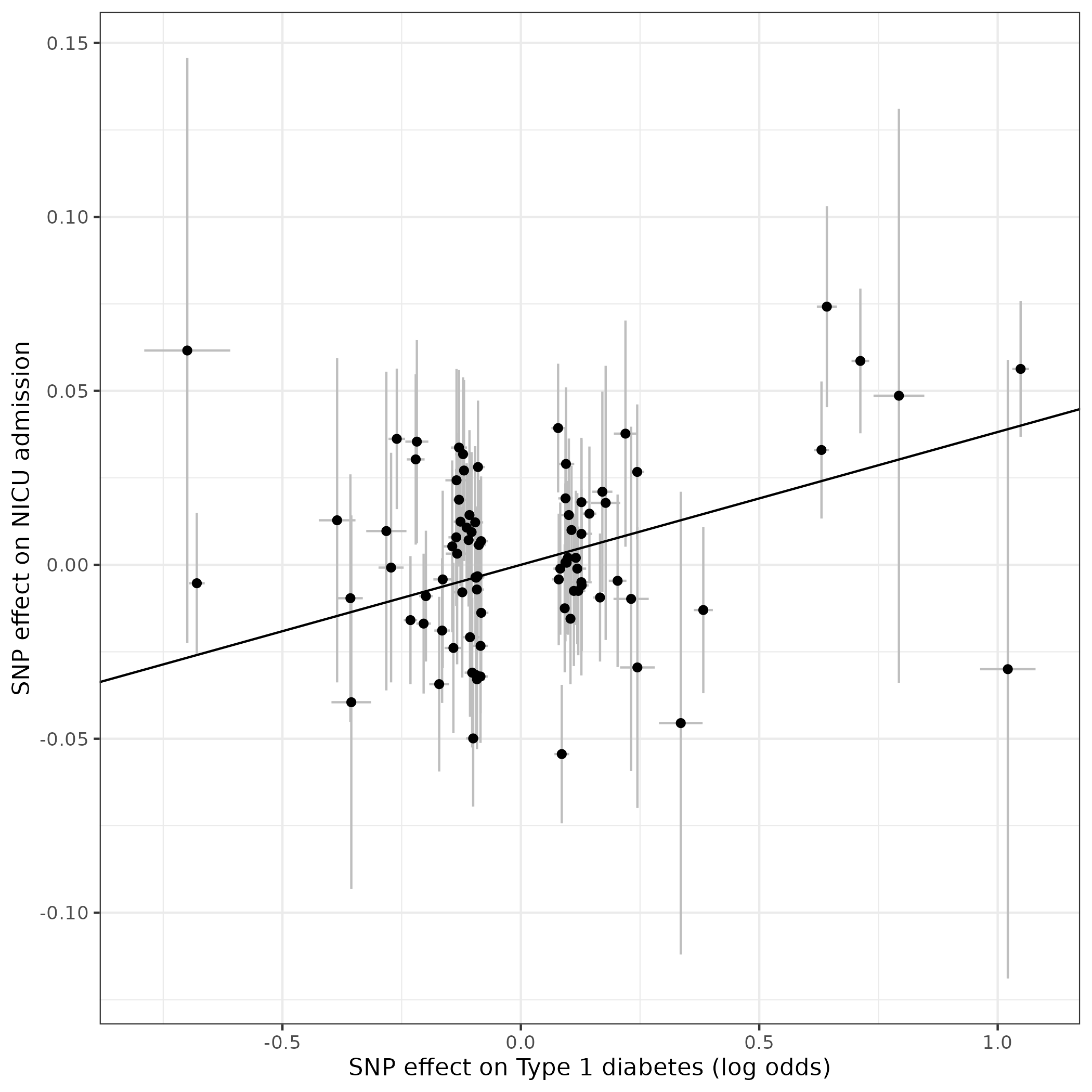


Figure 18. SNP-condition and SNP-outcome scatterplots for the effect of type 1 diabetes genetic liability (log odds) on neonatal intensive care unit (NICU) admission (log odds). Error bars indicate one standard error either side of beta point estimate, black line illustrates the inverse variance weighted estimator.


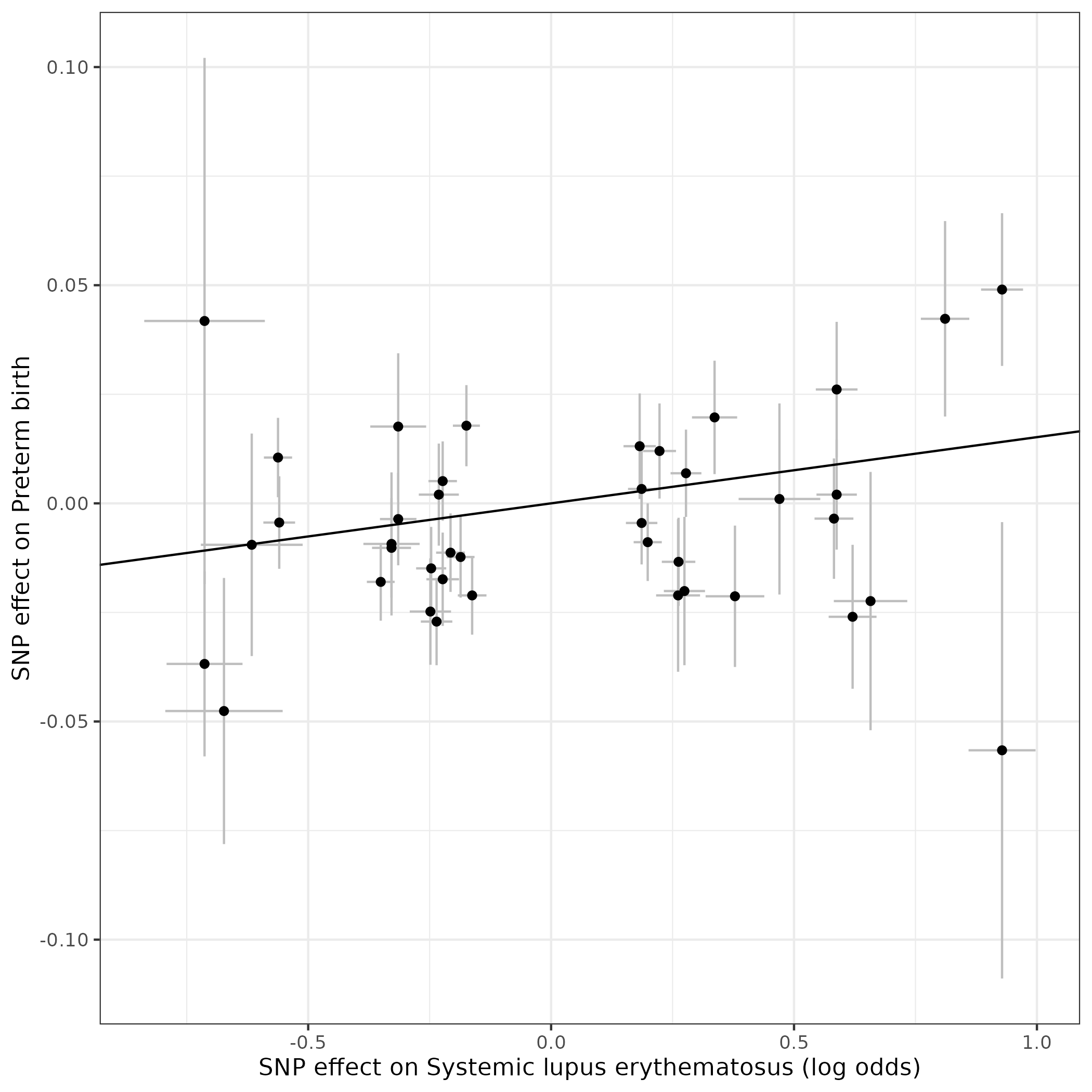


Figure 19. SNP-condition and SNP-outcome scatterplots for the effect systemic lupus erythematosus genetic liability (log odds) on preterm birth (log odds). Error bars indicate one standard error either side of beta point estimate, black line illustrates the inverse variance weighted estimator.


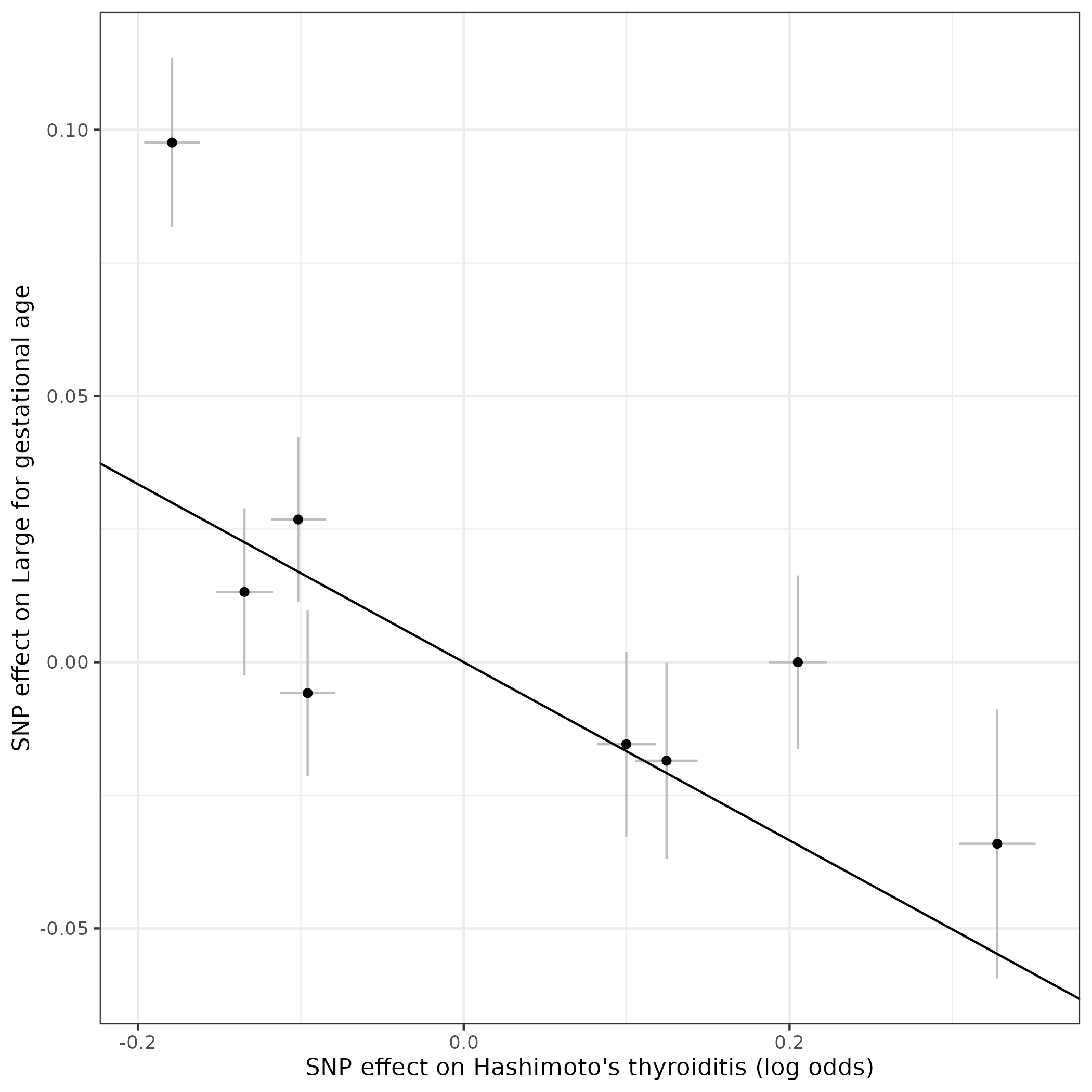


Figure 20. SNP-condition and SNP-outcome scatterplots for the effect of Hashimoto’s thyroiditis genetic liability (log odds) on large-for-gestational-age (log odds). Error bars indicate one standard error either side of beta point estimate, black line illustrates the inverse variance weighted estimator.


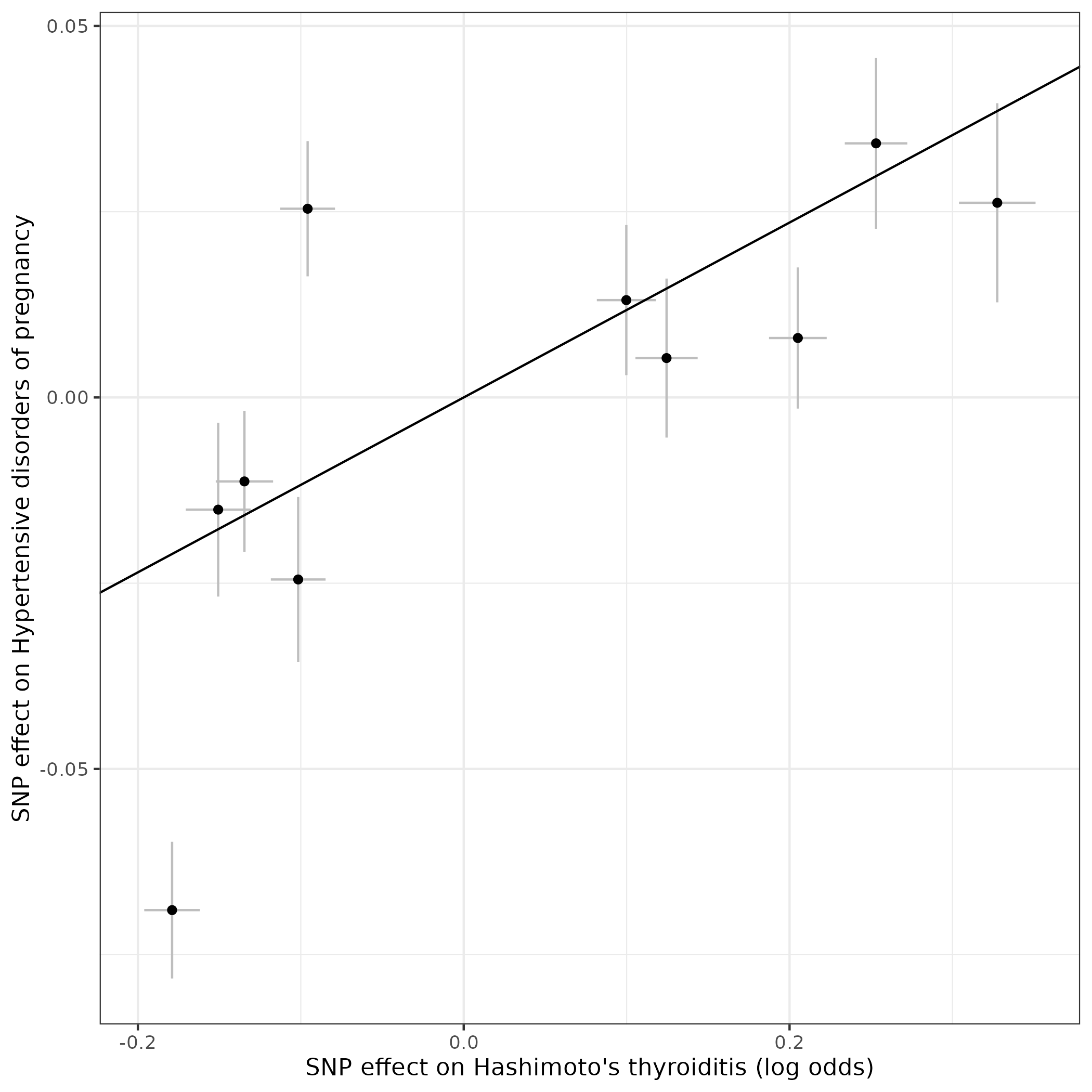


Figure 21. SNP-condition and SNP-outcome scatterplots for the effect of Hashimoto’s thyroiditis genetic liability (log odds) on hypertensive disorders of pregnancy (log odds). Error bars indicate one standard error either side of beta point estimate, black line illustrates the inverse variance weighted estimator.


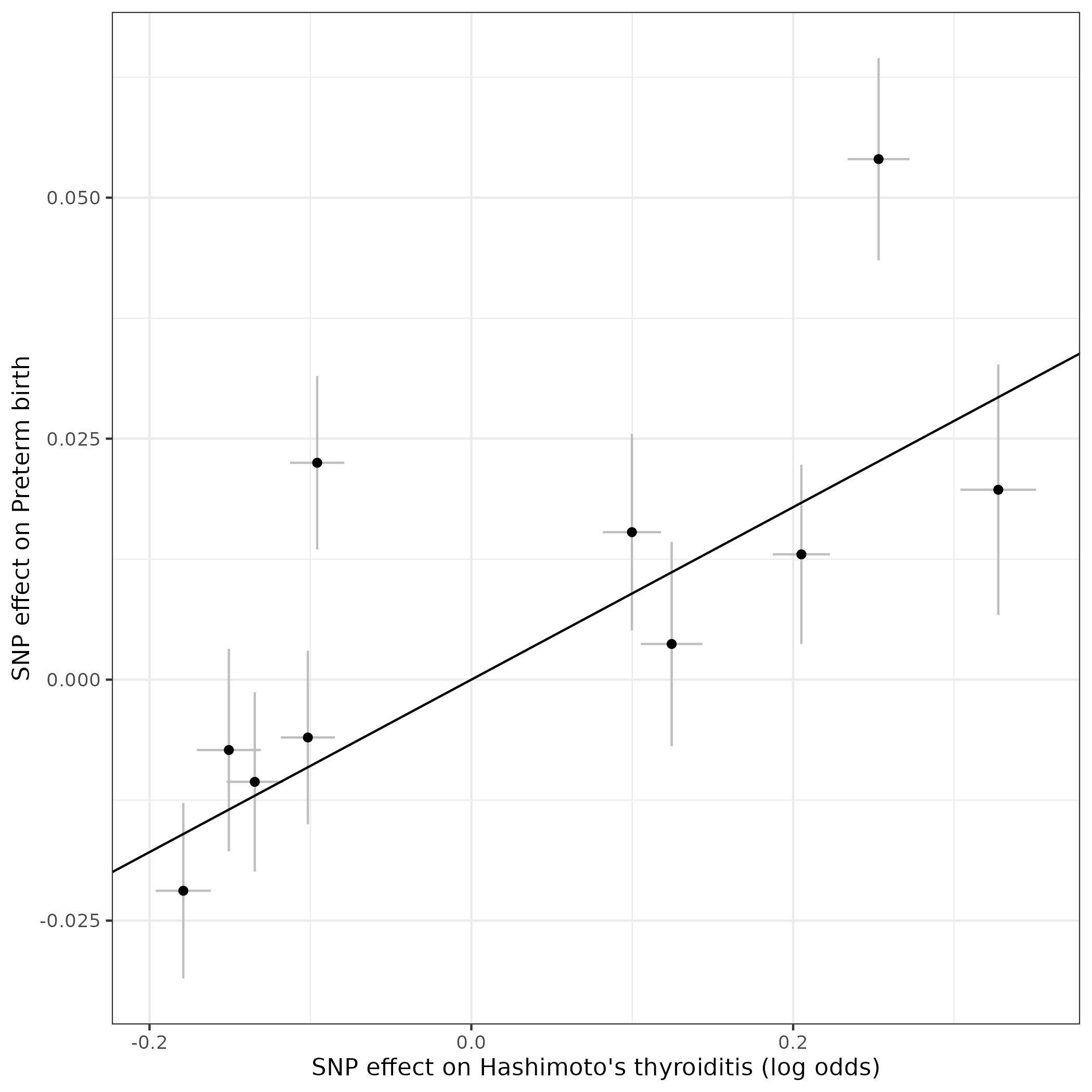


Figure 22. SNP-condition and SNP-outcome scatterplots for the effect of Hashimoto’s thyroiditis genetic liability (log odds) on preterm birth (log odds). Error bars indicate one standard error either side of beta point estimate, black line illustrates the inverse variance weighted estimator.


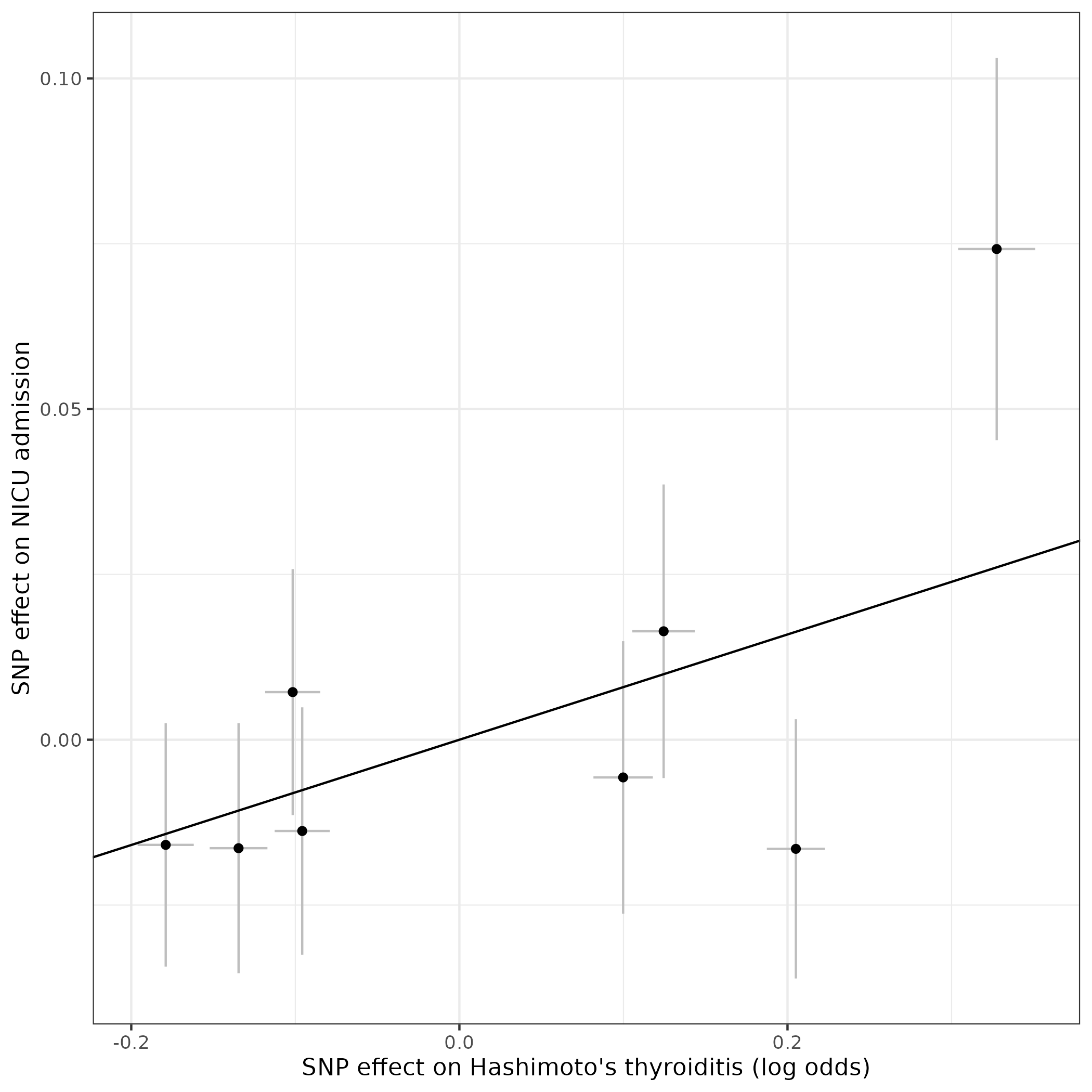


Figure 23. SNP-condition and SNP-outcome scatterplots for the effect of Hashimoto’s thyroiditis genetic liability (log odds) on neonatal intensive care unit (NICU) admission (log odds). Error bars indicate one standard error either side of beta point estimate, black line illustrates the inverse variance weighted estimator.


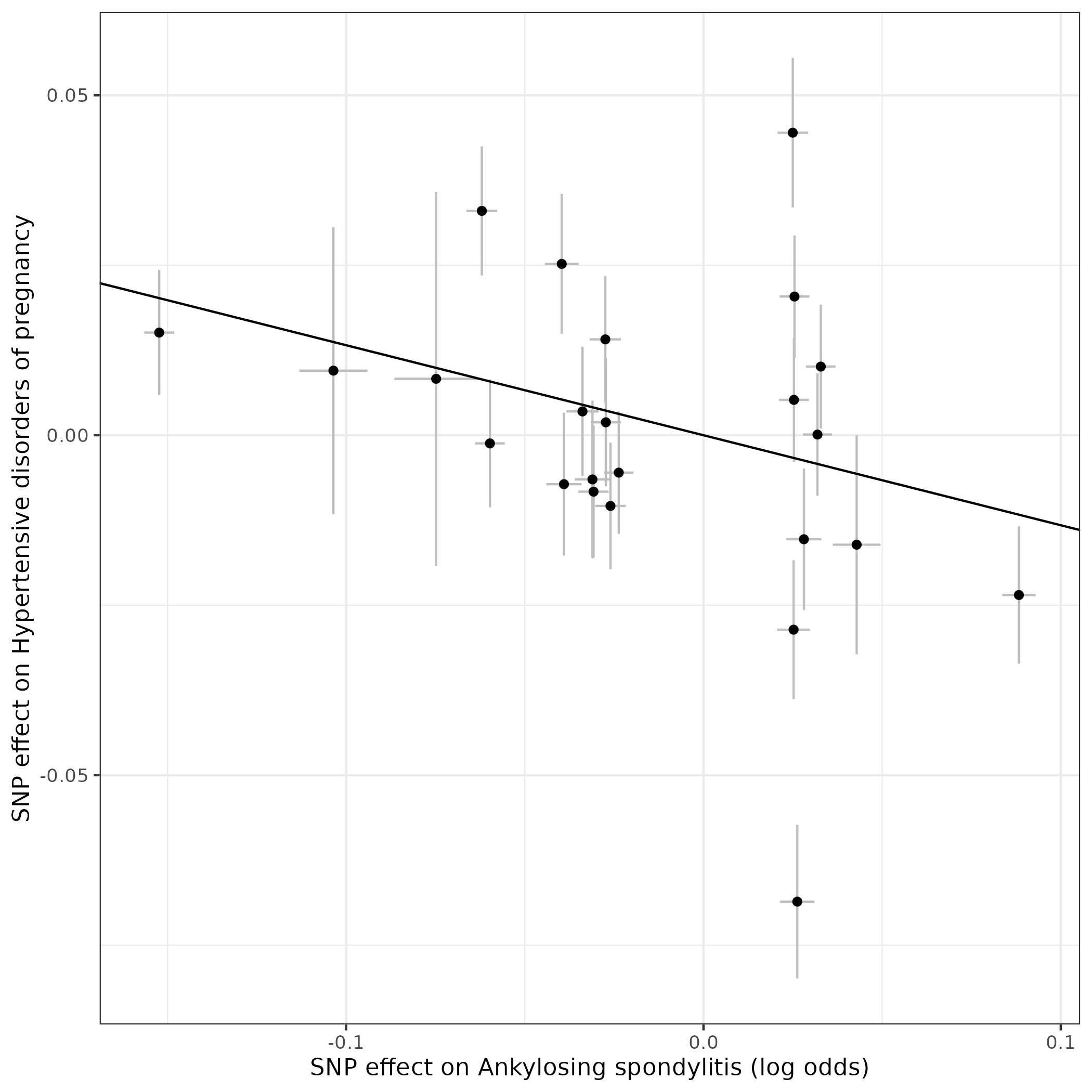


Figure 24. SNP-condition and SNP-outcome scatterplots for the effect of ankylosing spondylitis genetic liability (log odds) on hypertensive disorders of pregnancy (log odds). Error bars indicate one standard error either side of beta point estimate, black line illustrates the inverse variance weighted estimator.


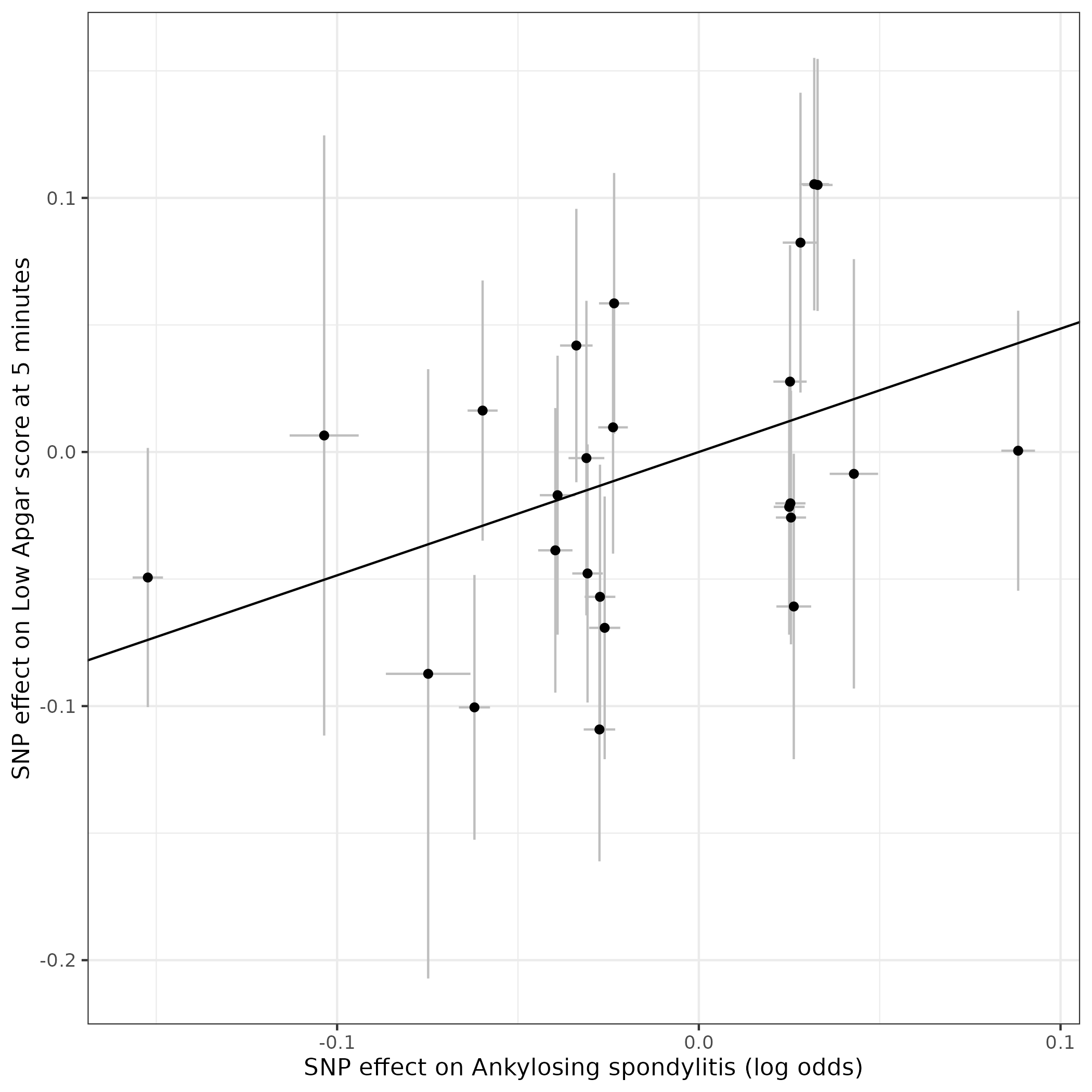


Figure 25. SNP-condition and SNP-outcome scatterplots for the effect of ankylosing spondylitis genetic liability (log odds) on low Apgar score after 5 minutes (log odds). Error bars indicate one standard error either side of beta point estimate, black line illustrates the inverse variance weighted estimator.

## Pleiotropy-robust MR methods and excluding HLA variants


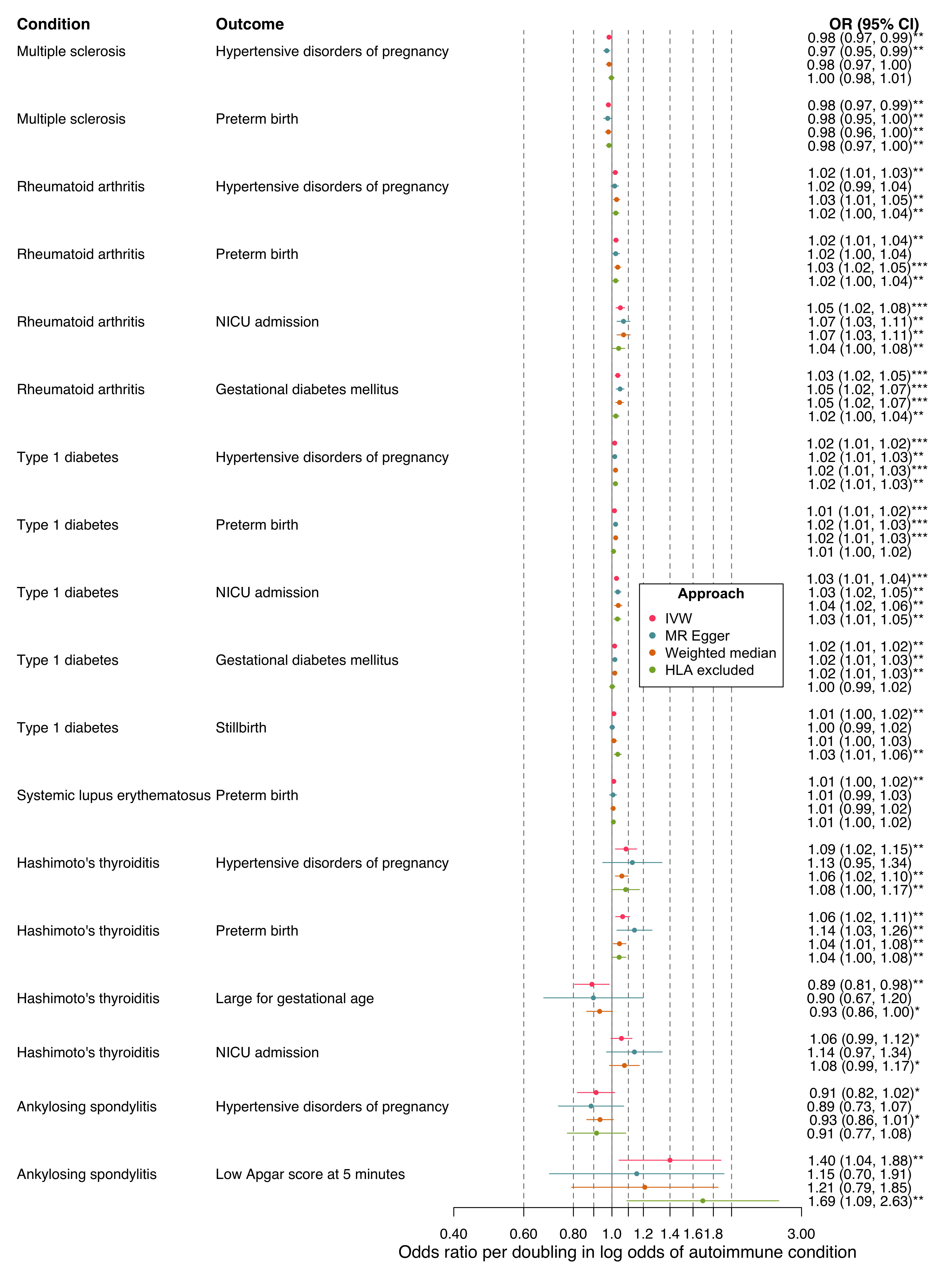


Figure 26. Pleiotropy-robust methods and HLA excluded Mendelian randomization estimates for the effects of increased log odds of autoimmune conditions on pregnancy outcomes, for results selected for follow up. Estimates reflect odds ratio of pregnancy outcome per doubling in log odds of autoimmune condition. NICU = neonatal intensive care unit. IVW = inverse variance weighted. Effects excluding HLA could not be estimated for two effects of Hashimoto’s thyroiditis liability since no HLA SNPs were available in these outcome datasets (n = 2 SNPs located in HLA for Hashimoto’s thyroiditis instruments). * indicates p<0.1 and change in odds of 5% or more, ** indicates p < 0.05, *** indicates p<0.05 after correcting for multiple testing using false discovery rate.

## Steiger filtering


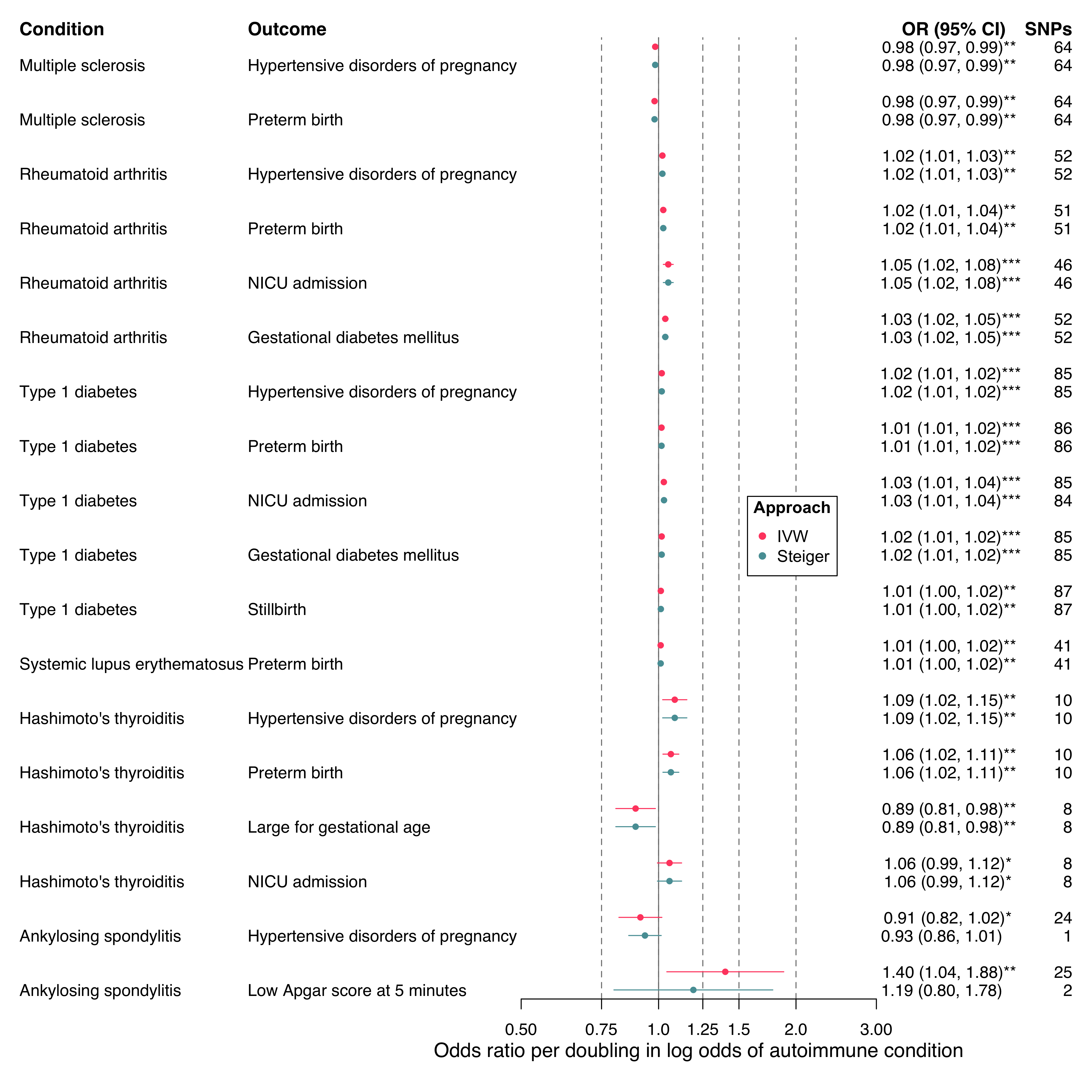


Figure 27. Steiger filtered Mendelian randomization effect estimates exploring potential reverse causation in the effects of increased log odds of autoimmune conditions on pregnancy outcomes, for results selected for follow up. Estimates reflect odds ratio of pregnancy outcome per doubling in log odds of autoimmune condition for mother. NICU = neonatal intensive care unit. * indicates p<0.1 and change in odds of 5% or more, ** indicates p<0.05, *** indicates p<0.05 after correcting for multiple testing using false discovery rate.

## Fetal genetic effects


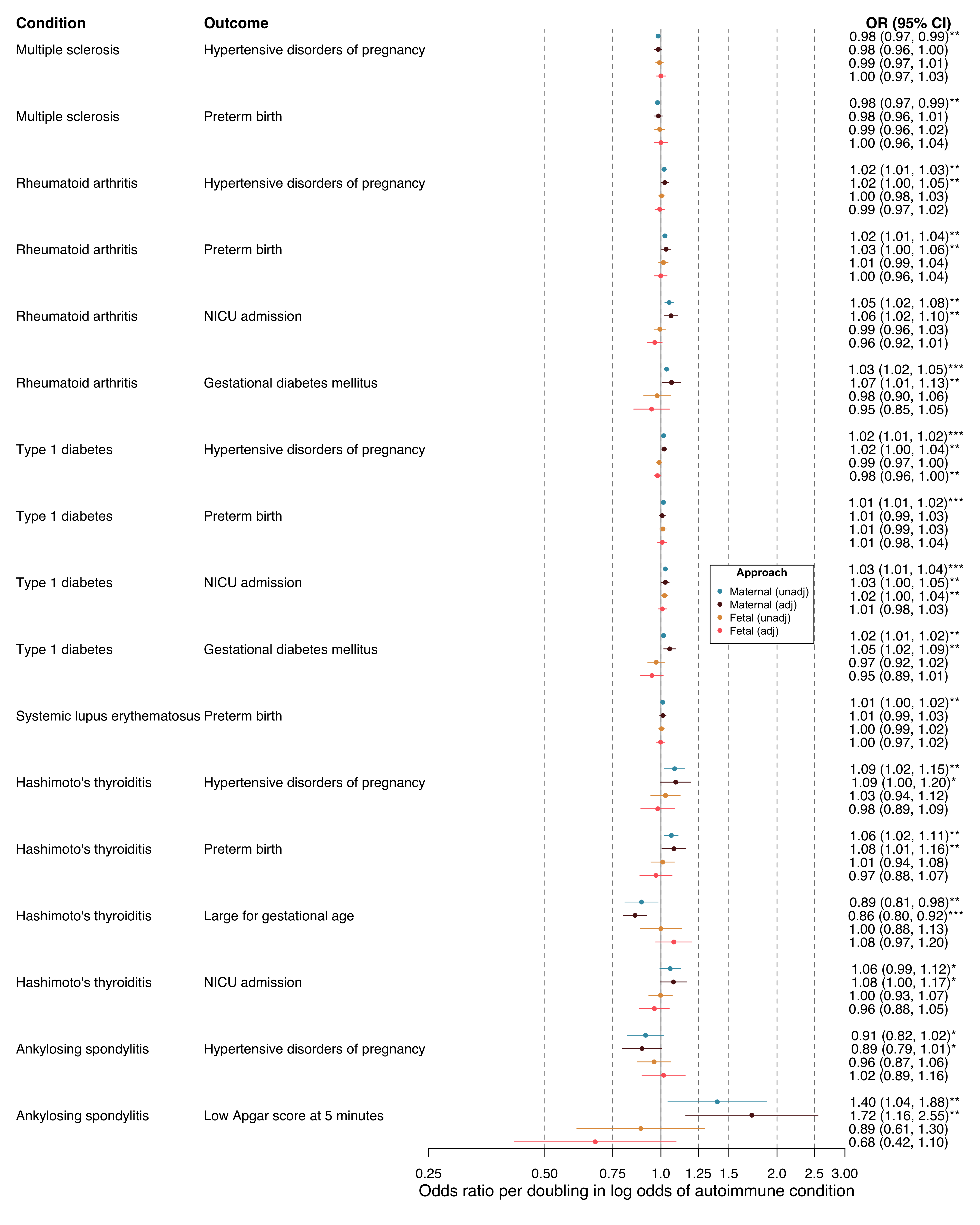


Figure 28. Weighted linear model Mendelian randomization effect estimates exploring fetal genetic effects on the effects of increased log odds of autoimmune conditions on pregnancy outcomes, for results selected for follow up. Estimates reflect odds ratio of pregnancy outcome per doubling in log odds of autoimmune condition. Models shown are 1) maternal effects (unadjusted; same as main analysis inverse-variance weighted (IVW) estimates), 2) maternal effects adjusted for fetal genotype, 3) fetal effects (unadjusted), and 4) fetal effects adjusted for maternal genotype. Estimates reflect odds ratio of pregnancy outcome per doubling in log odds of autoimmune condition for mother (models 1 & 2) or fetus (models 3 & 4). NICU = neonatal intensive care unit. The effect of type 1 diabetes liability on stillbirth is not included here since fetal genotypes are not available for this outcome. * indicates p<0.1 and change in odds of 5% or more, ** indicates p<0.05, *** indicates p<0.05 after correcting for multiple testing using false discovery rate.

## Leave one study out


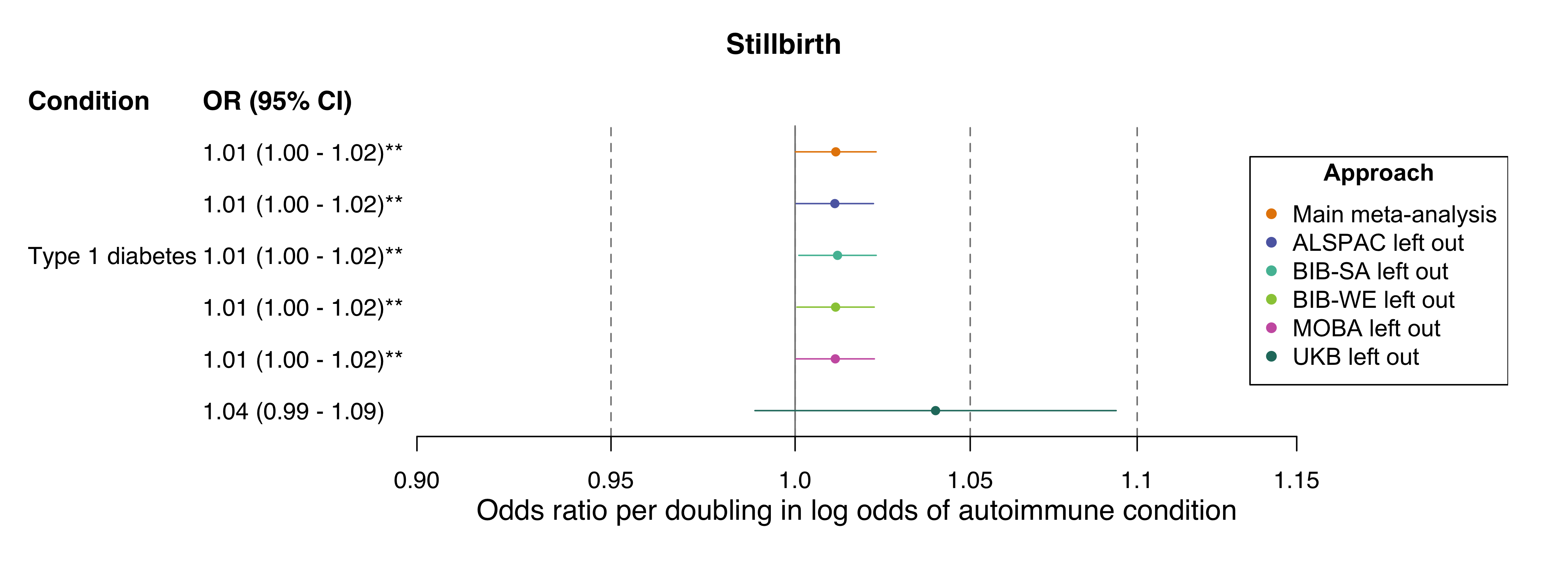


Figure 29. Leave-one-study-out and main meta-analysis Mendelian randomization effect estimates for effects of increased log odds of autoimmune conditions on stillbirth, for results selected for follow up. Estimates reflect odds ratio of pregnancy outcome per doubling in log odds of autoimmune condition. * indicates p<0.1 and change in odds of 5% or more, ** indicates p<0.05, *** indicates p<0.05 after correcting for multiple testing using false discovery rate.


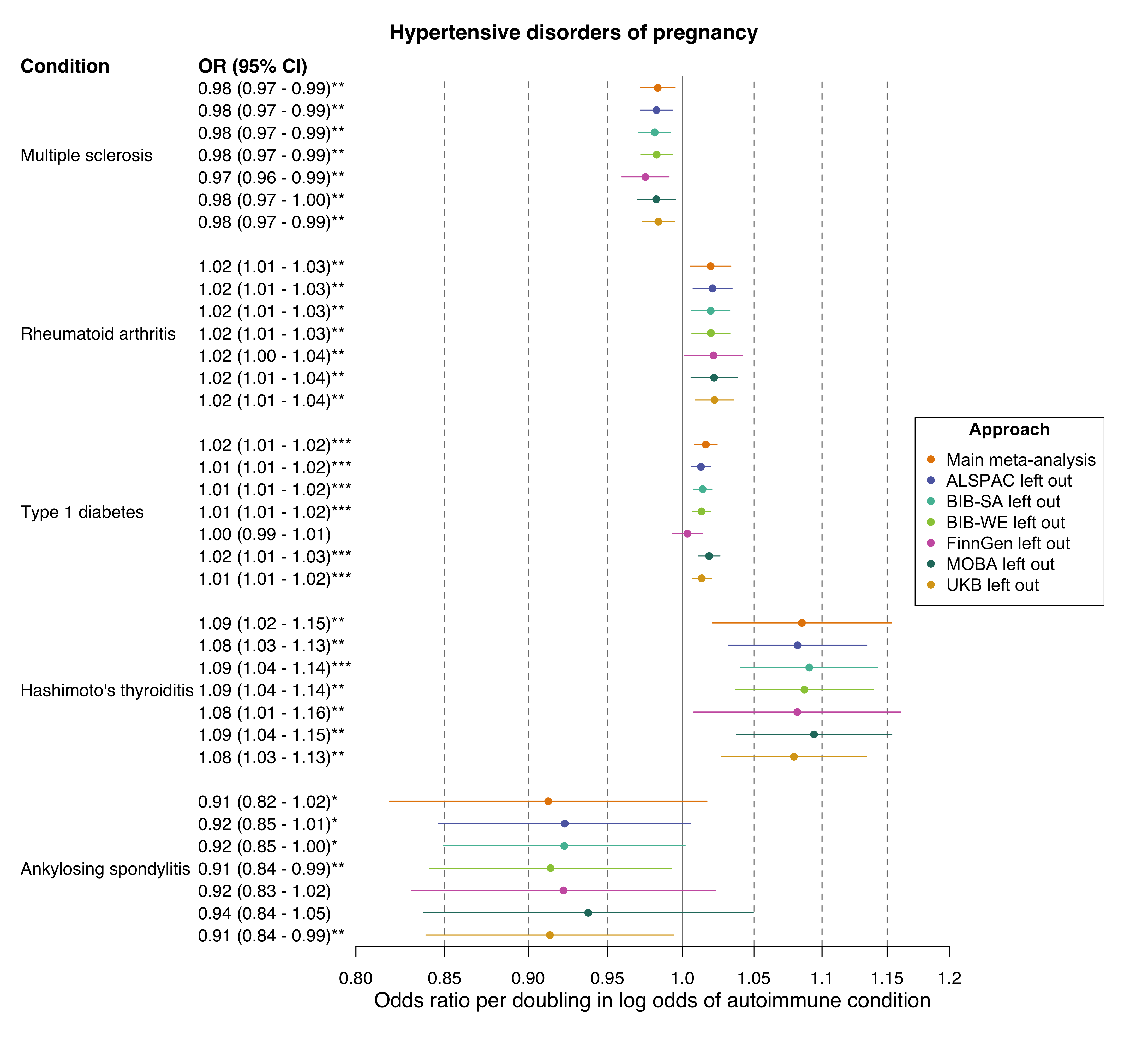


Figure 30. Leave-one-study-out and main meta-analysis Mendelian randomization effect estimates for effects of increased log odds of autoimmune conditions on hypertensive disorders of pregnancy, for results selected for follow up. Estimates reflect odds ratio of pregnancy outcome per doubling in log odds of autoimmune condition.* indicates p<0.1 and change in odds of 5% or more, ** indicates p<0.05, *** indicates p<0.05 after correcting for multiple testing using false discovery rate.


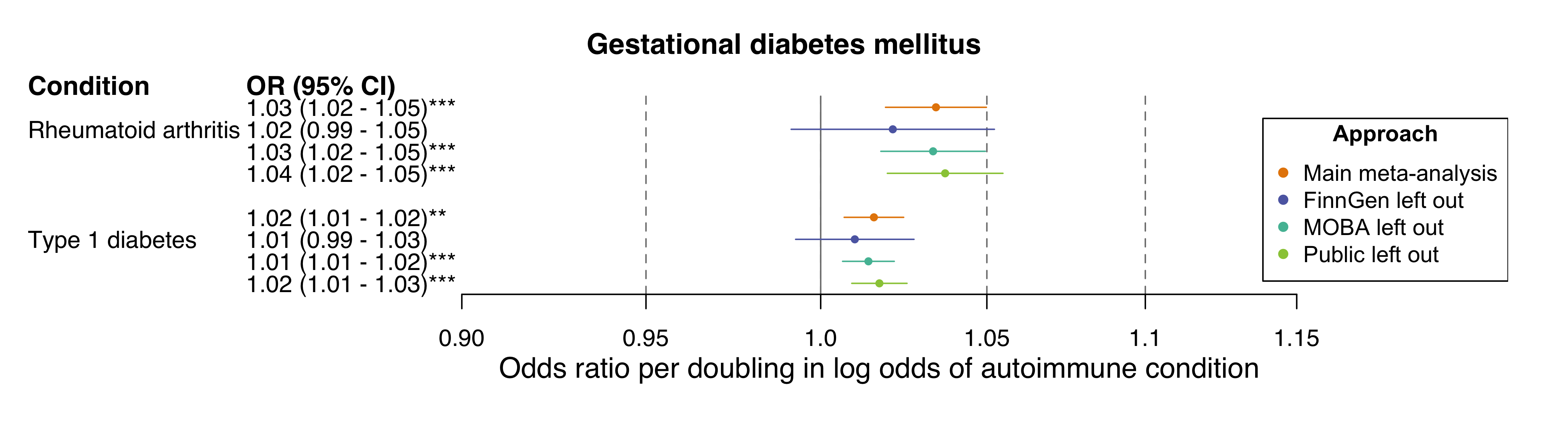


Figure 31. Leave-one-study-out and main meta-analysis Mendelian randomization effect estimates for effects of increased log odds of autoimmune conditions on gestational diabetes mellitus, for results selected for follow up. Estimates reflect odds ratio of pregnancy outcome per doubling in log odds of autoimmune condition.* indicates p<0.1 and change in odds of 5% or more, ** indicates p<0.05, *** indicates p<0.05 after correcting for multiple testing using false discovery rate.


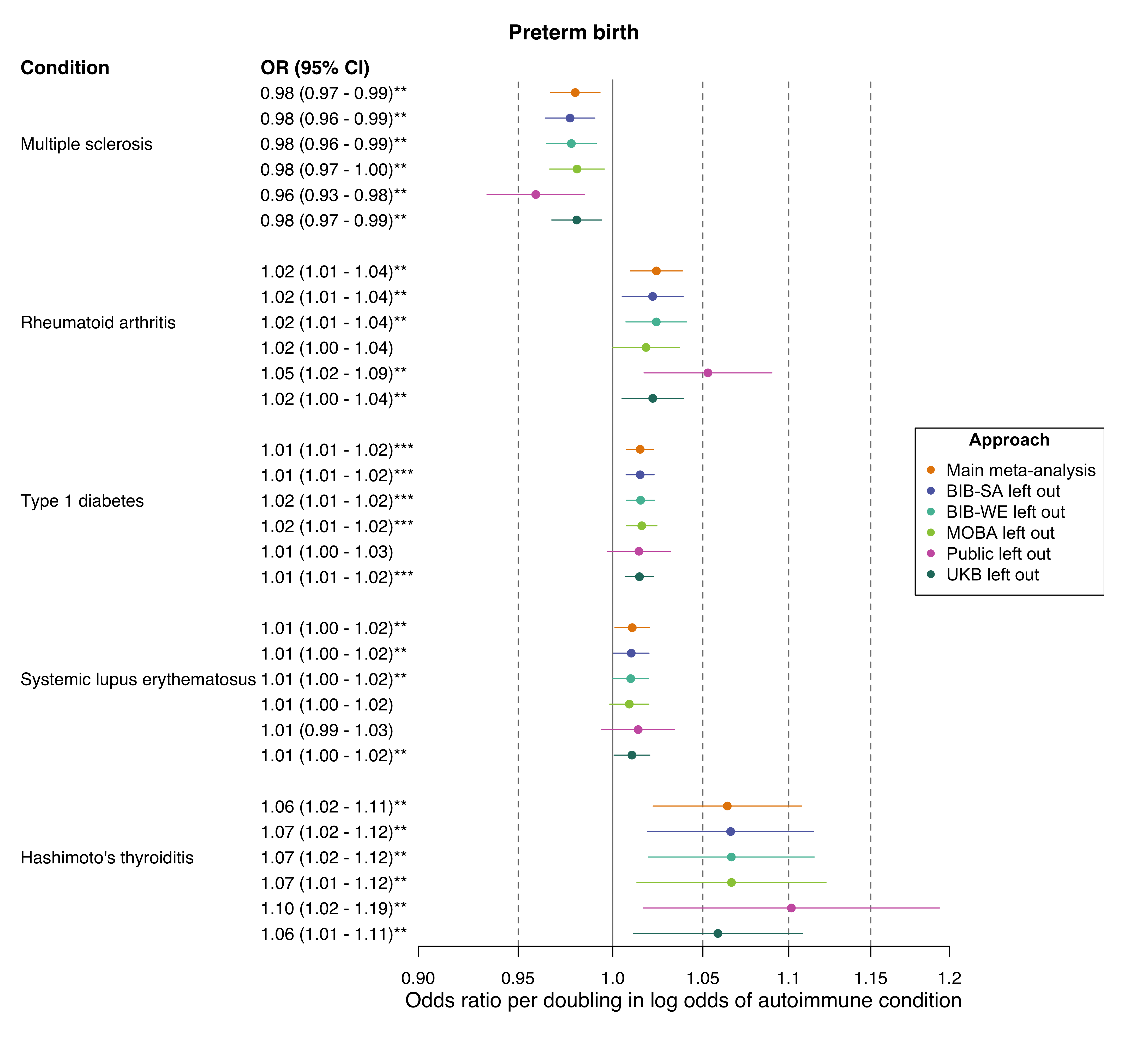


Figure 32. Leave-one-study-out and main meta-analysis Mendelian randomization effect estimates for effects of increased log odds of autoimmune conditions on preterm birth, for results selected for follow up. Estimates reflect odds ratio of pregnancy outcome per doubling in log odds of autoimmune condition. * indicates p<0.1 and change in odds of 5% or more, ** indicates p<0.05, *** indicates p<0.05 after correcting for multiple testing using false discovery rate.


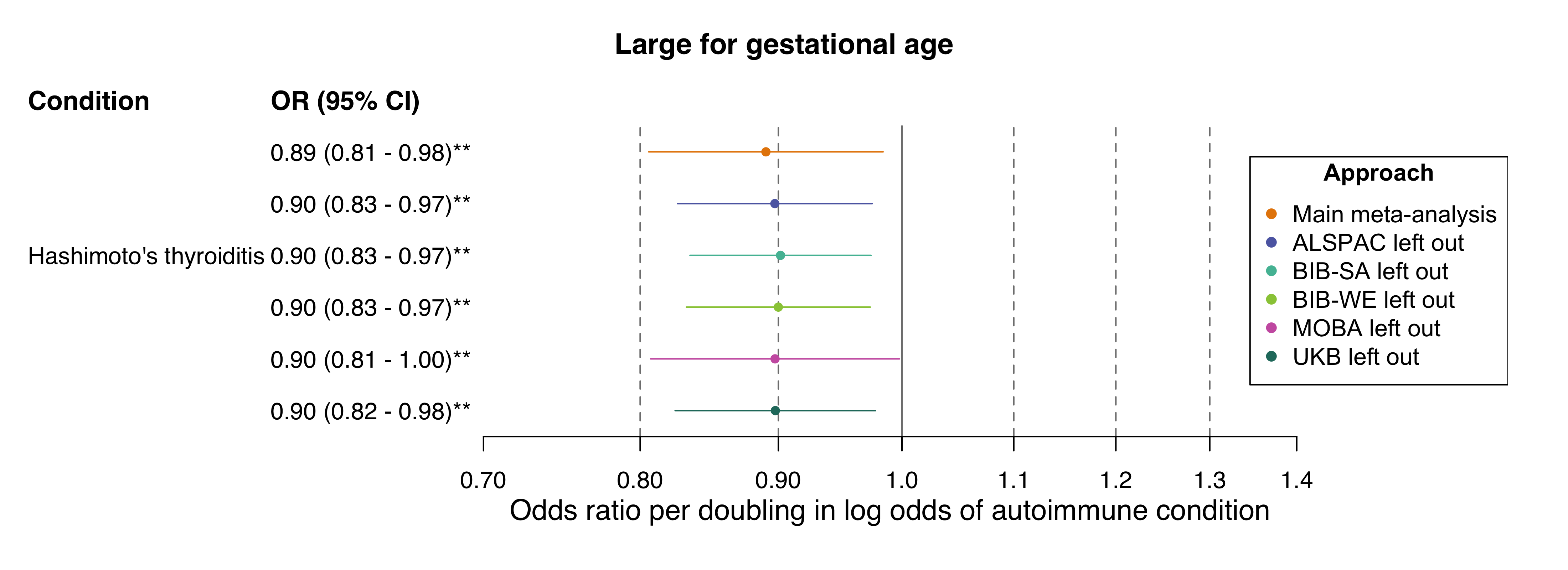


Figure 33. Leave-one-study-out and main meta-analysis Mendelian randomization effect estimates for effects of increased log odds of autoimmune conditions on large for gestational age, for results selected for follow up. Estimates reflect odds ratio of pregnancy outcome per doubling in log odds of autoimmune condition. * indicates p<0.1 and change in odds of 5% or more, ** indicates p<0.05, *** indicates p<0.05 after correcting for multiple testing using false discovery rate.


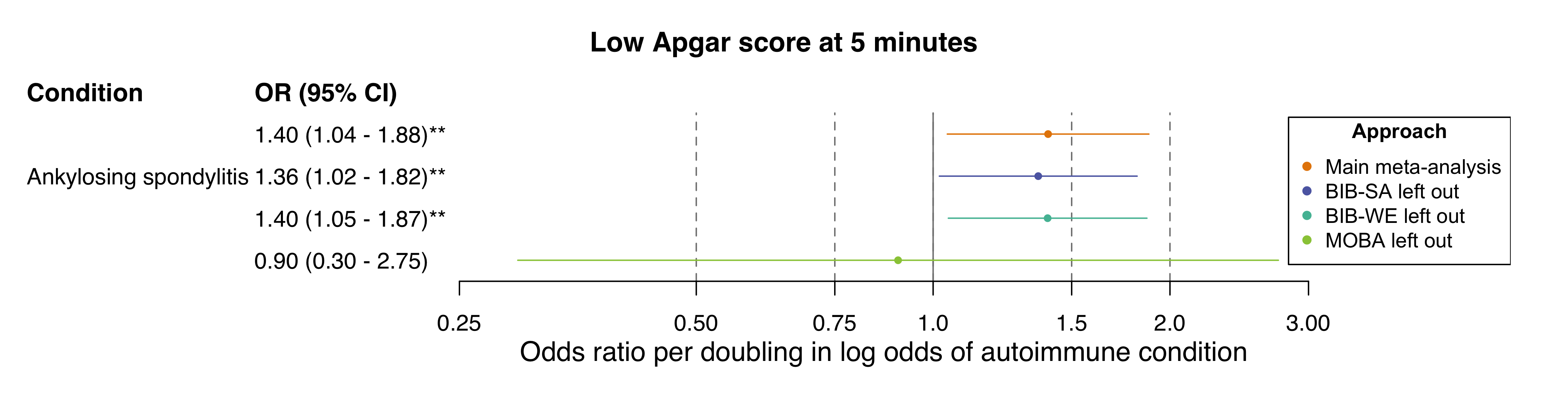


Figure 34. Leave-one-study-out and main meta-analysis Mendelian randomization effect estimates for effects of increased log odds of autoimmune conditions on low Apgar score at 5 minutes, for results selected for follow up. Estimates reflect odds ratio of pregnancy outcome per doubling in log odds of autoimmune condition. * indicates p<0.1 and change in odds of 5% or more, ** indicates p<0.05, *** indicates p<0.05 after correcting for multiple testing using false discovery rate.


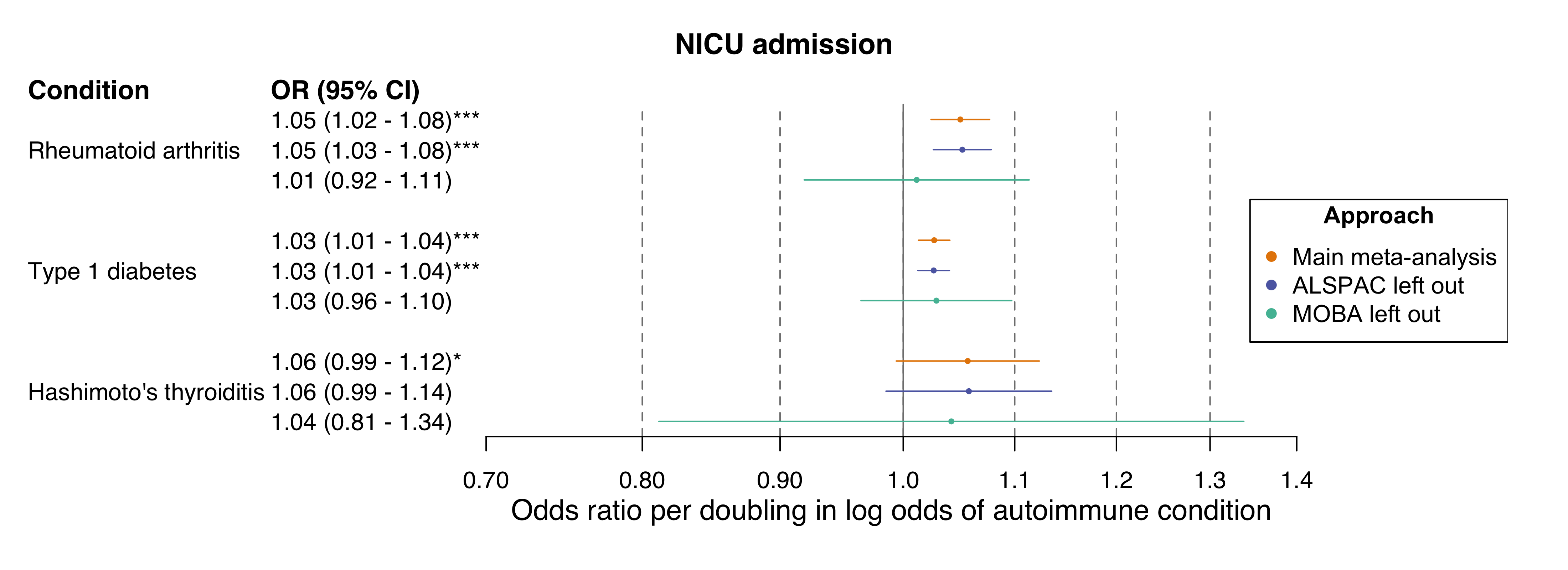


Figure 35. Leave-one-study-out and main meta-analysis Mendelian randomization effect estimates for effects of increased log odds of autoimmune conditions on NICU admission, for results selected for follow up. Estimates reflect odds ratio of pregnancy outcome per doubling in log odds of autoimmune condition. NICU = neonatal intensive care unit. * indicates p<0.1 and change in odds of 5% or more, ** indicates p<0.05, *** indicates p<0.05 after correcting for multiple testing using false discovery rate.

## Leave one SNP out estimates


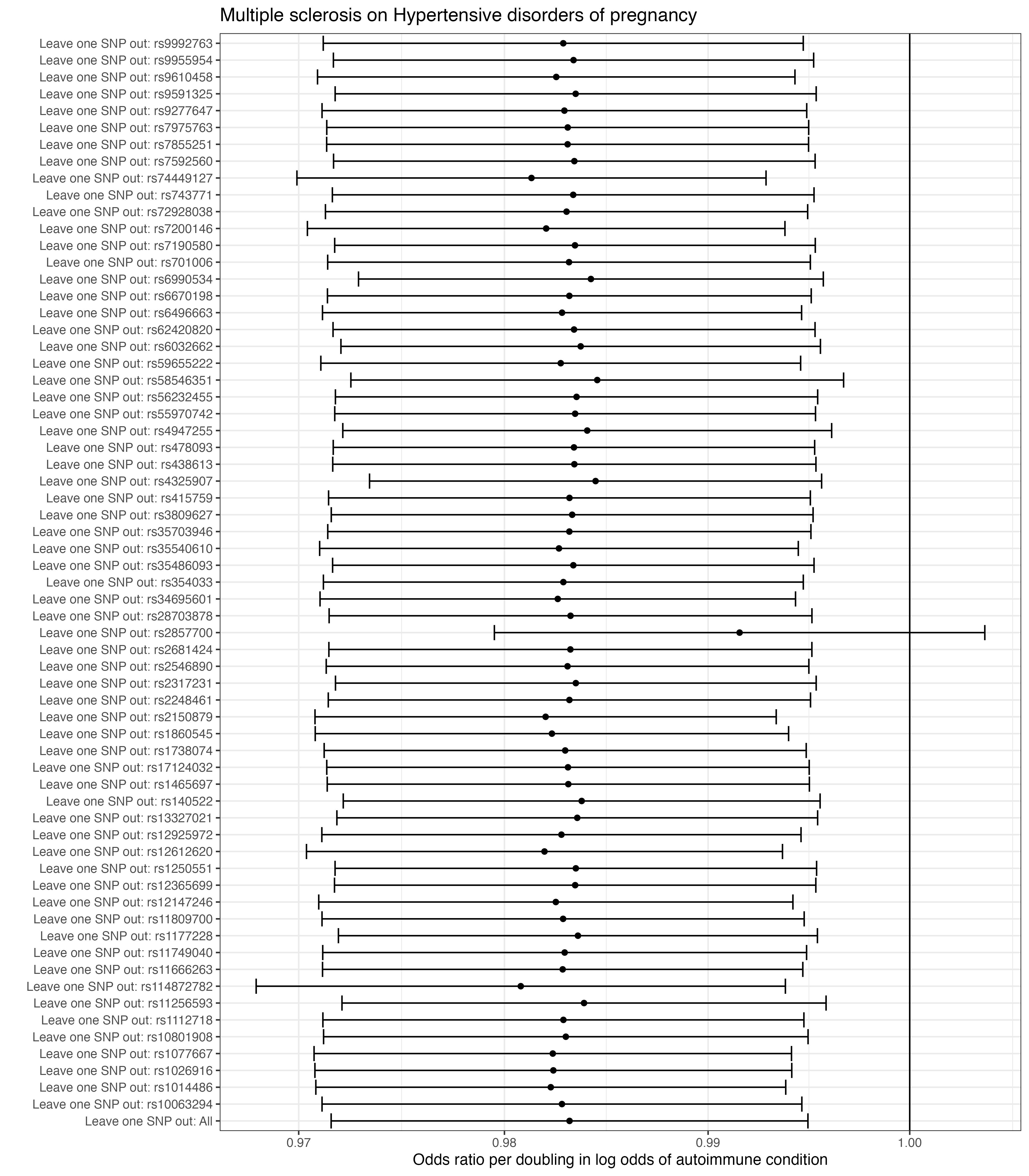


Figure 36. Leave-one-SNP-out Mendelian randomization estimates for the effect of multiple sclerosis on hypertensive disorders of pregnancy. Estimates reflect odds ratio of pregnancy outcome per doubling in log odds of autoimmune condition.


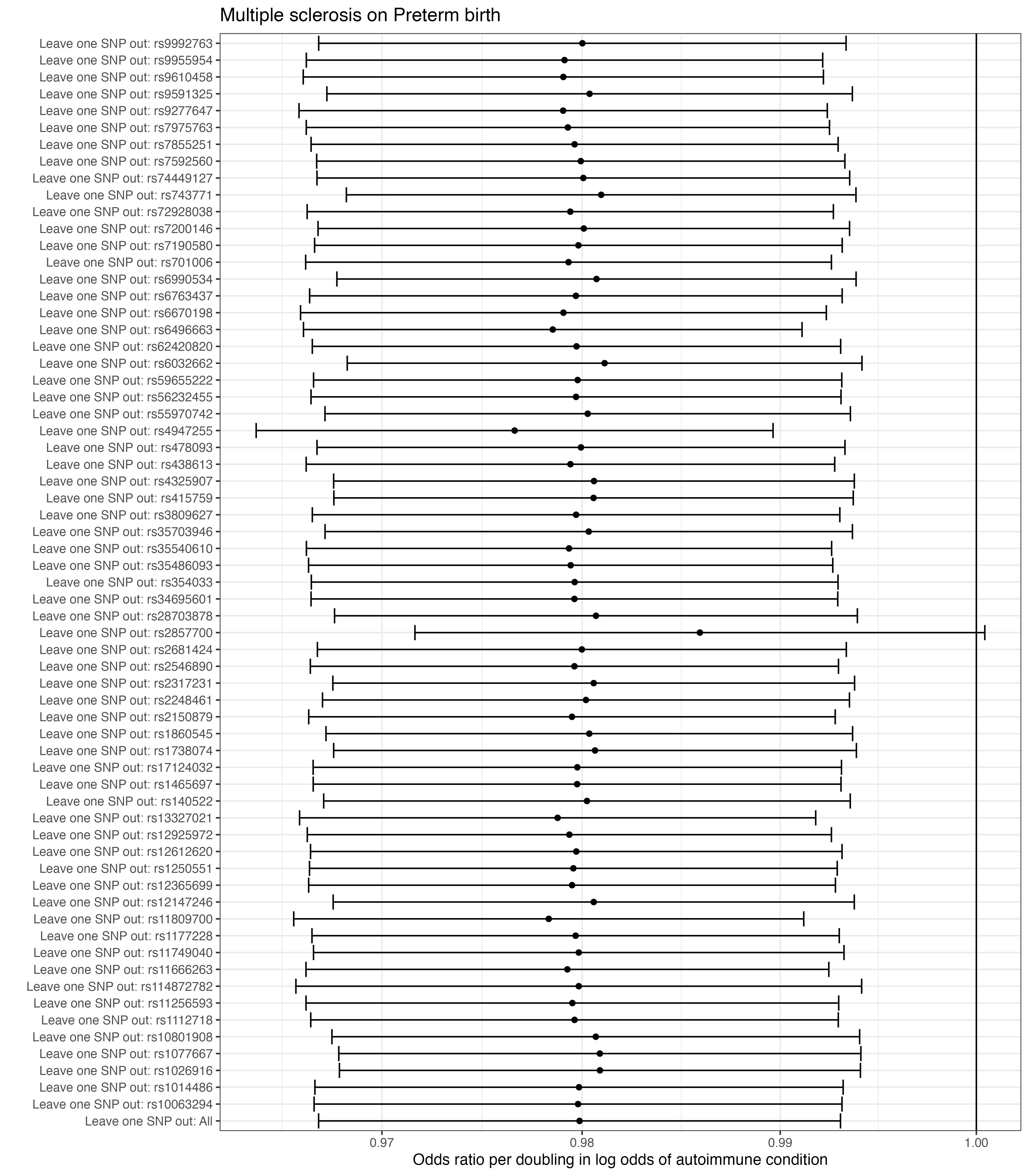


Figure 37. Leave-one-SNP-out Mendelian randomization estimates for the effect of multiple sclerosis on preterm birth. Estimates reflect odds ratio of pregnancy outcome per doubling in log odds of autoimmune condition.


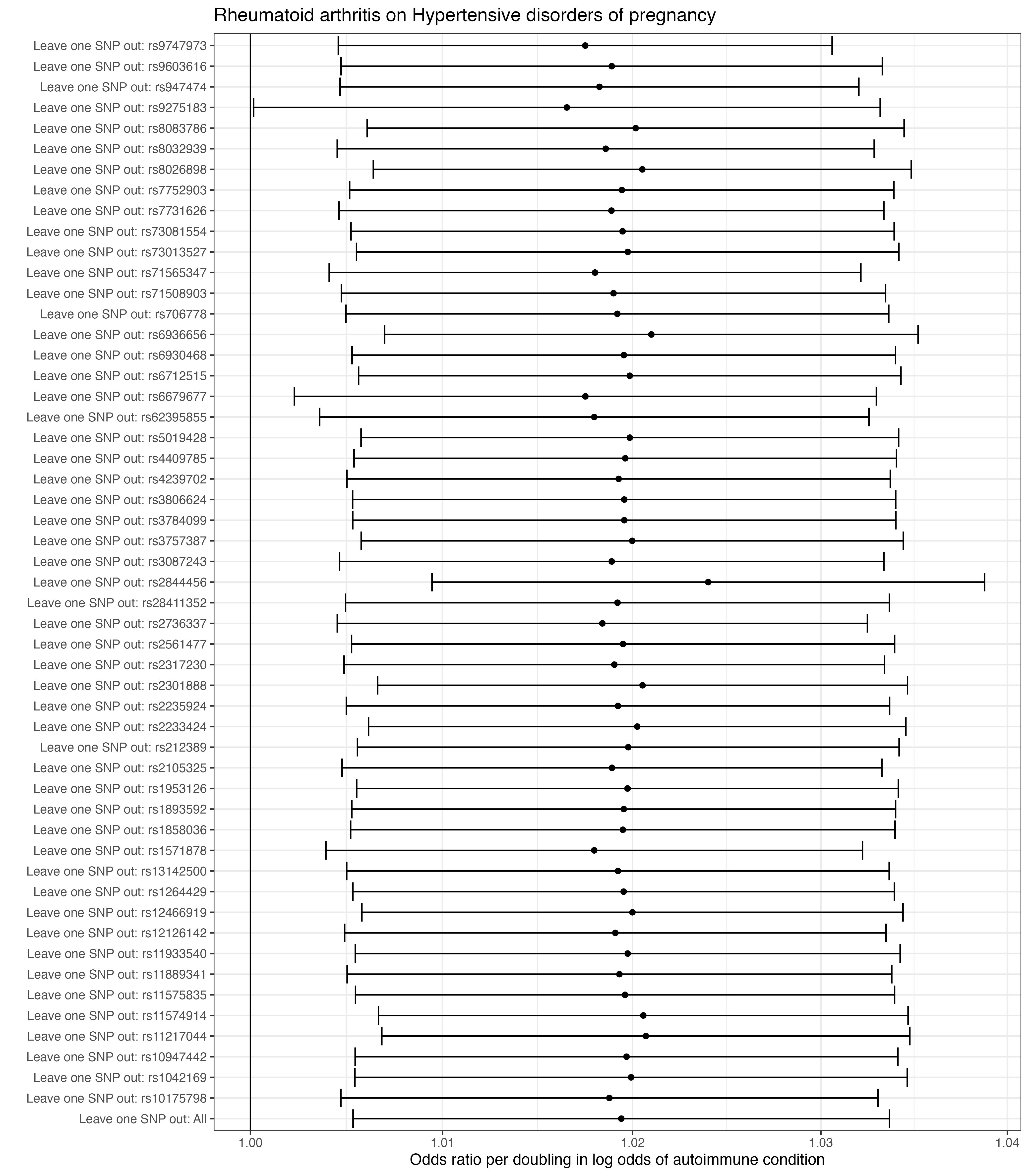


Figure 38. Leave-one-SNP-out Mendelian randomization estimates for the effect of rheumatoid arthritis on hypertensive disorders of pregnancy. Estimates reflect odds ratio of pregnancy outcome per doubling in log odds of autoimmune condition.


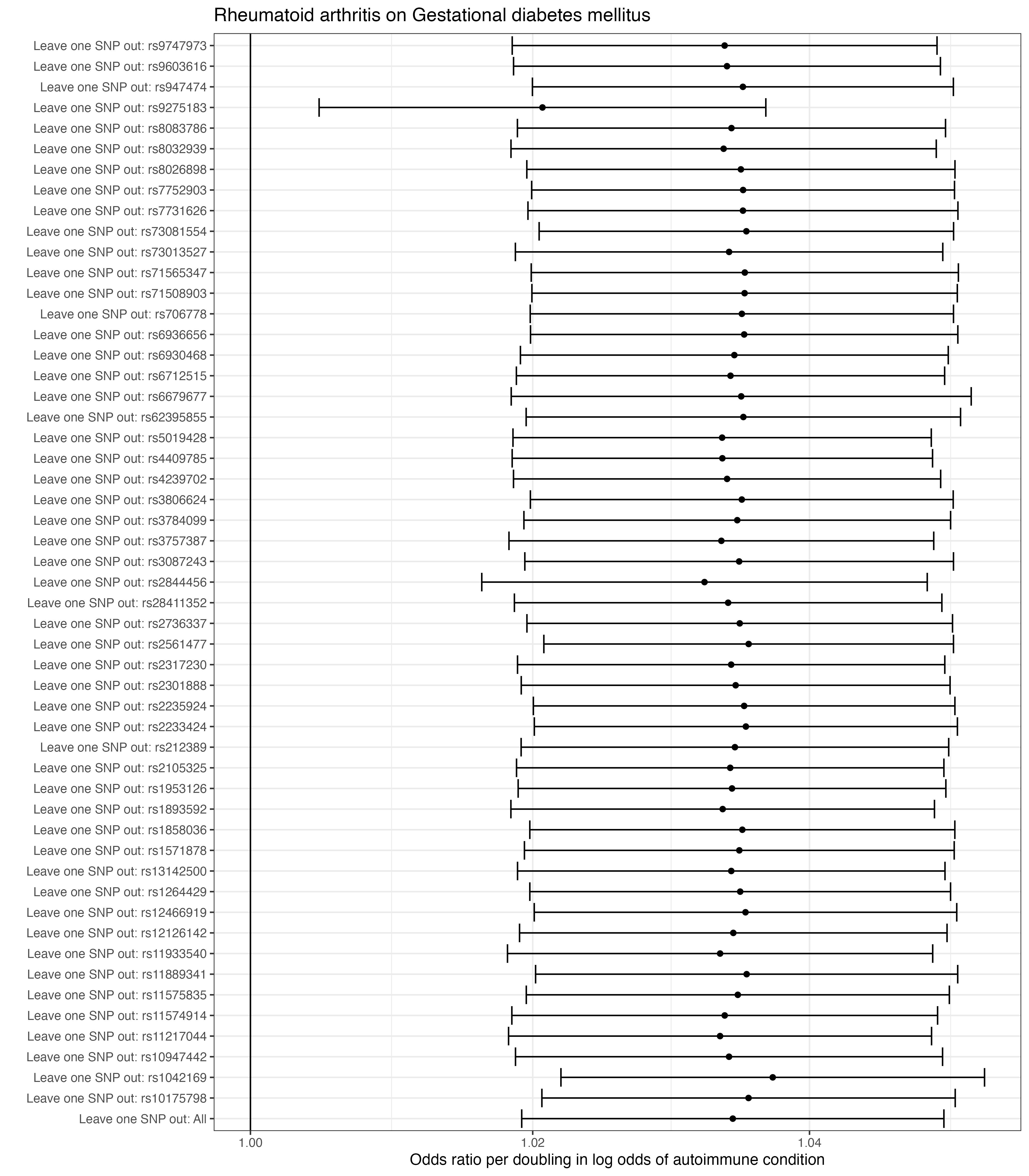


Figure 39. Leave-one-SNP-out Mendelian randomization estimates for the effect of rheumatoid arthritis on gestational diabetes mellitus. Estimates reflect odds ratio of pregnancy outcome per doubling in log odds of autoimmune condition.


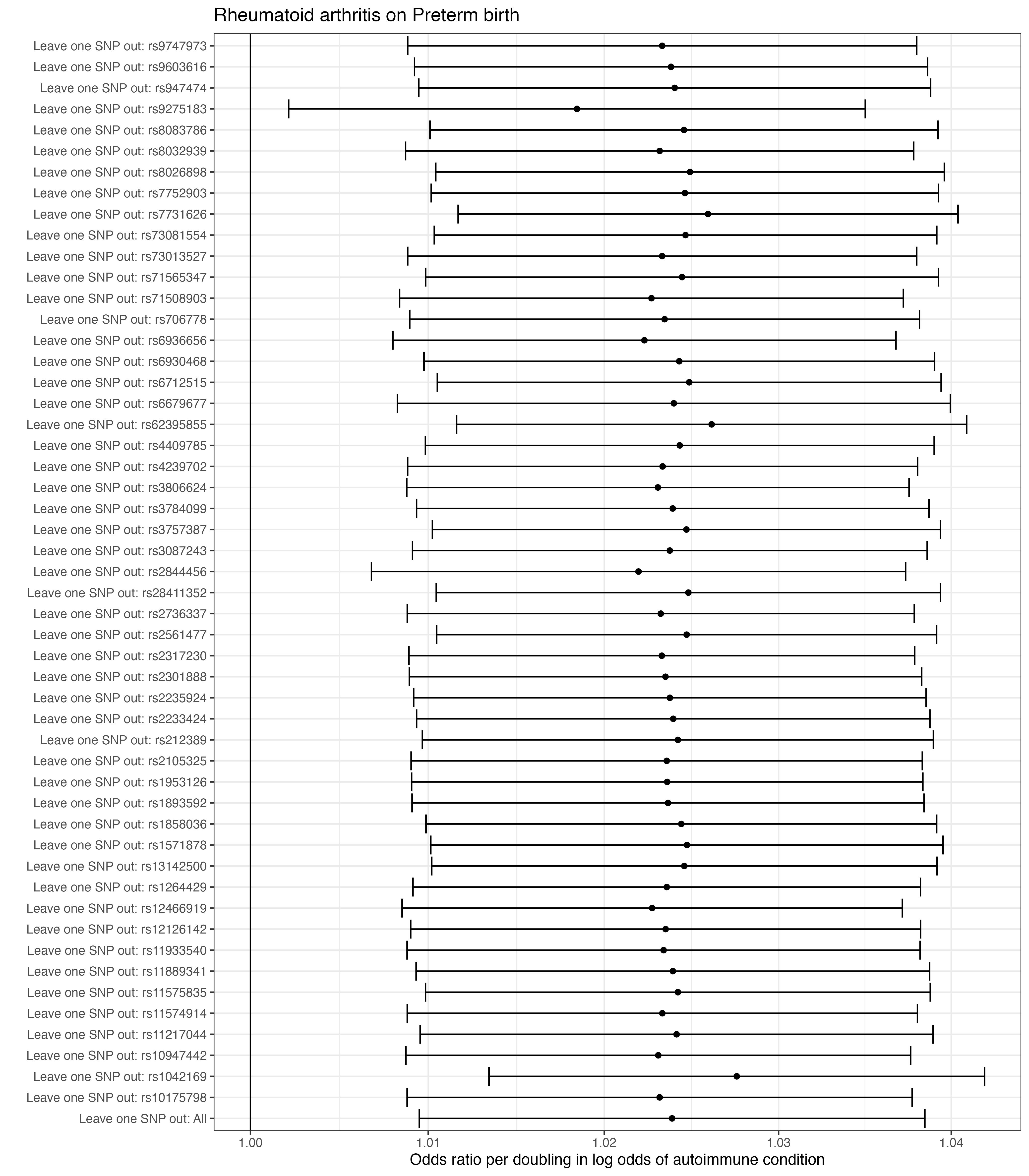


Figure 40. Leave-one-SNP-out Mendelian randomization estimates for the effect of rheumatoid arthritis on preterm birth. Estimates reflect odds ratio of pregnancy outcome per doubling in log odds of autoimmune condition.


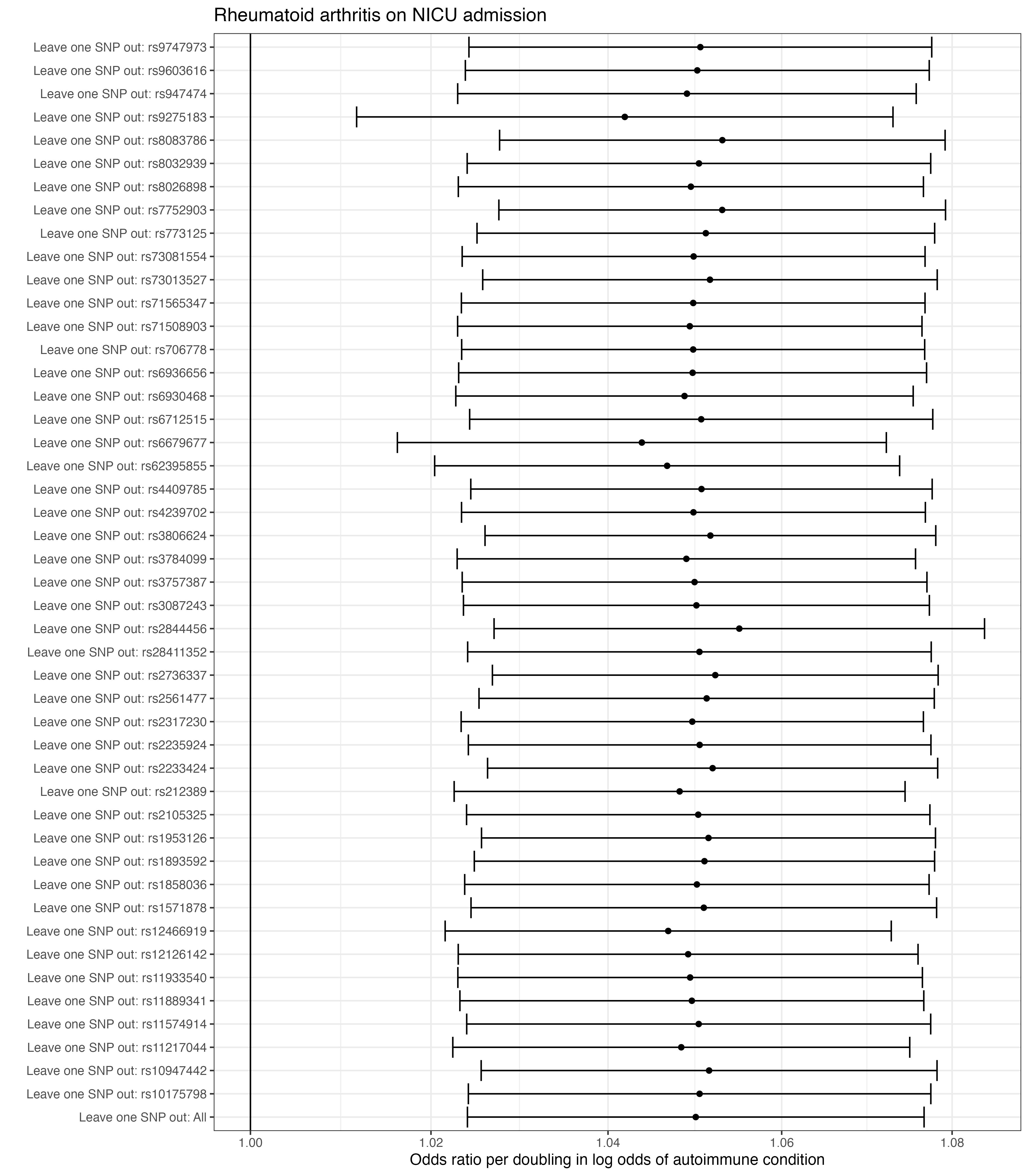


Figure 41. Leave-one-SNP-out Mendelian randomization estimates for the effect of rheumatoid arthritis on NICU admission. Estimates reflect odds ratio of pregnancy outcome per doubling in log odds of autoimmune condition.


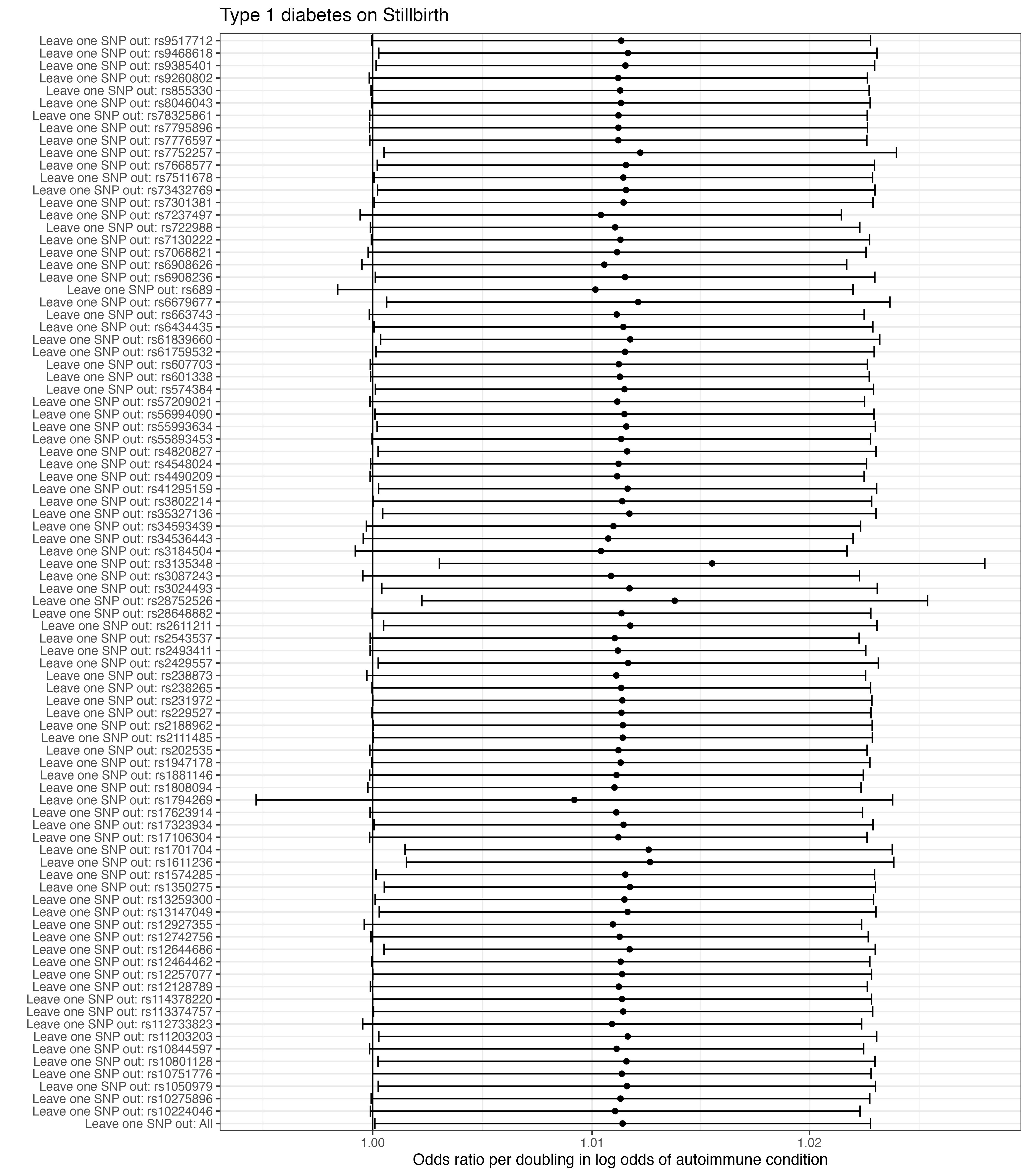


Figure 42. Leave-one-SNP-out Mendelian randomization estimates for the effect of type 1 diabetes on stillbirth. Estimates reflect odds ratio of pregnancy outcome per doubling in log odds of autoimmune condition.


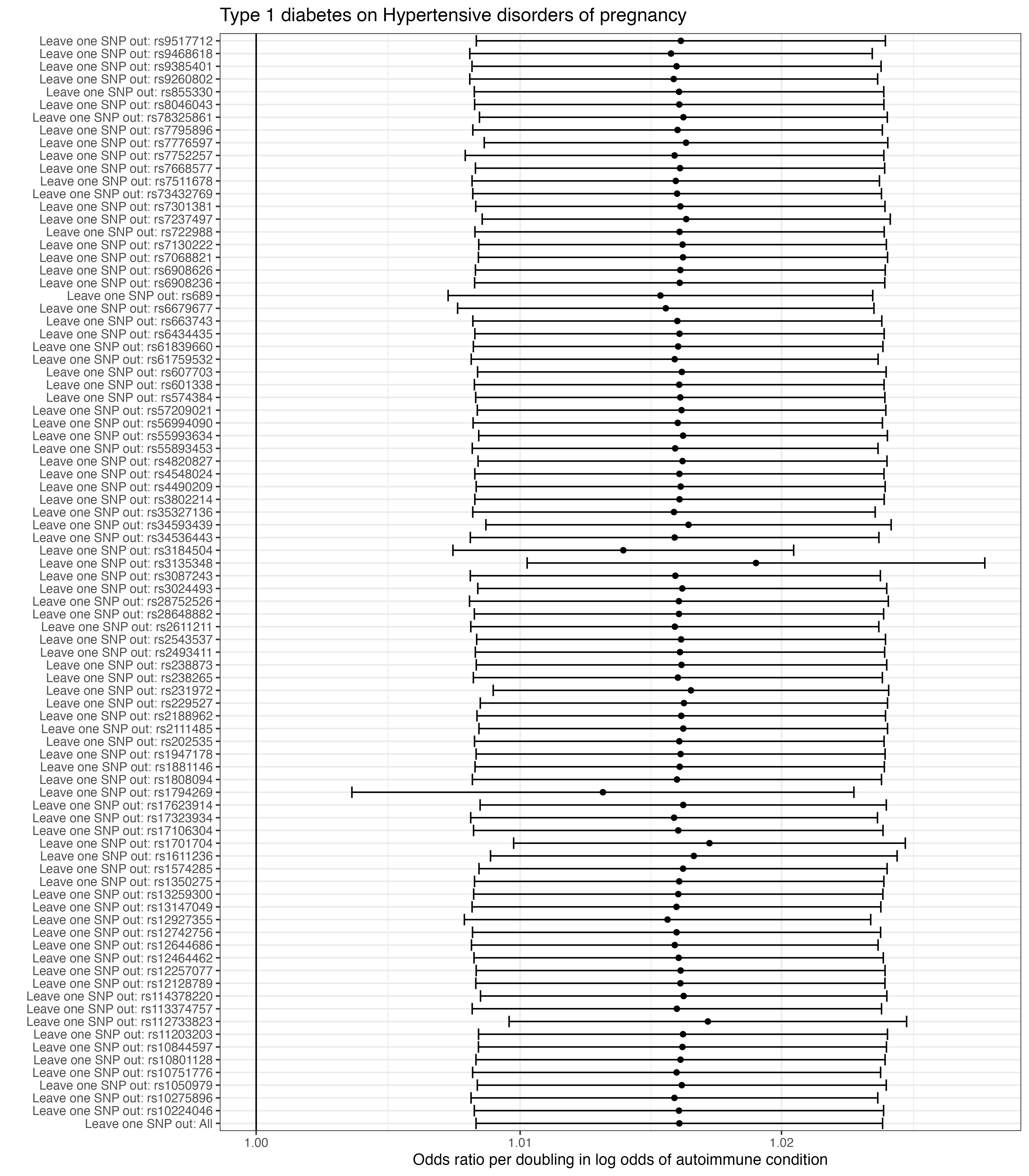


Figure 43. Leave-one-SNP-out Mendelian randomization estimates for the effect of type 1 diabetes on hypertensive disorders of pregnancy. Estimates reflect odds ratio of pregnancy outcome per doubling in log odds of autoimmune condition.


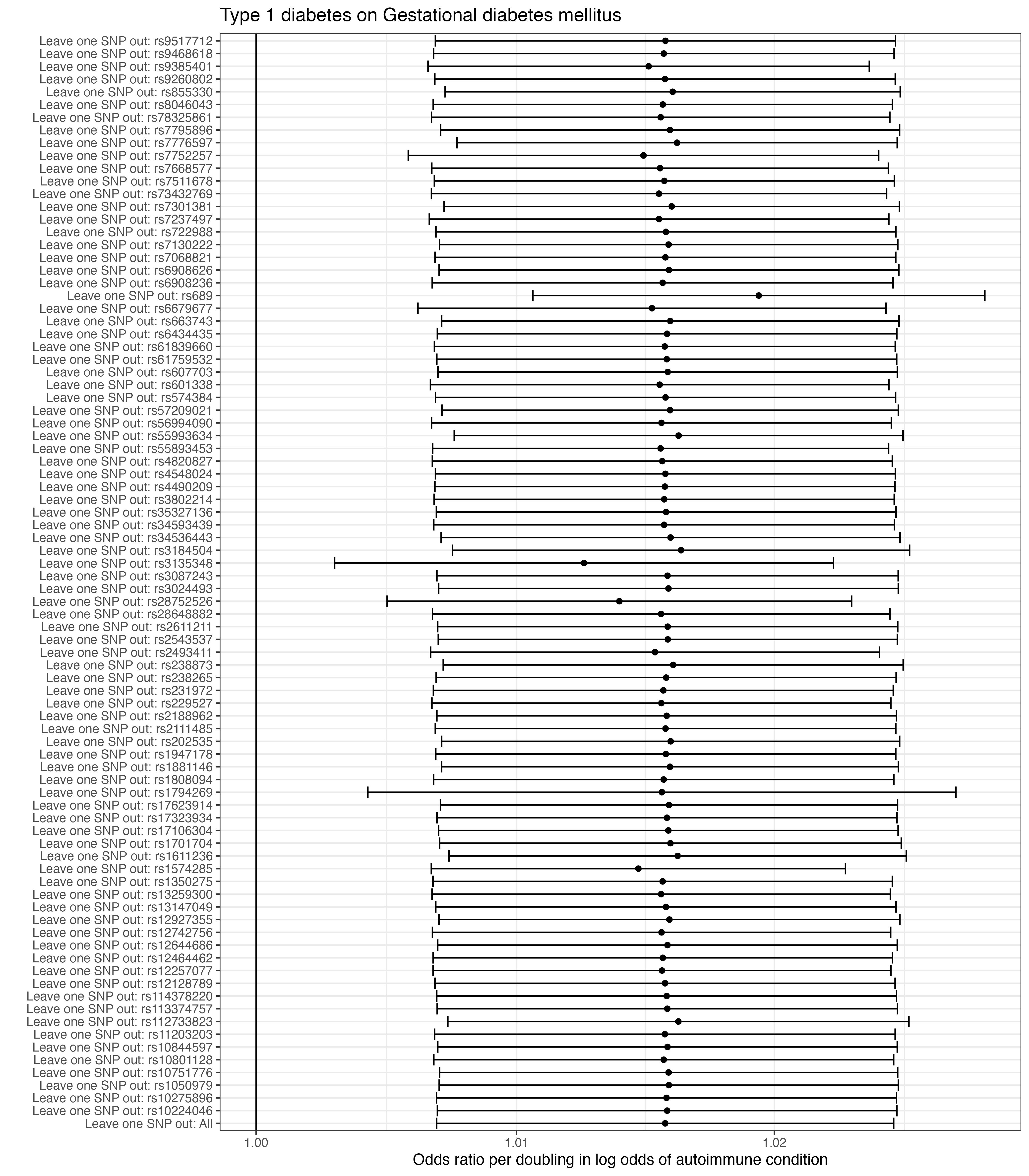


Figure 44. Leave-one-SNP-out Mendelian randomization estimates for the effect of type 1 diabetes on gestational diabetes mellitus. Estimates reflect odds ratio of pregnancy outcome per doubling in log odds of autoimmune condition.


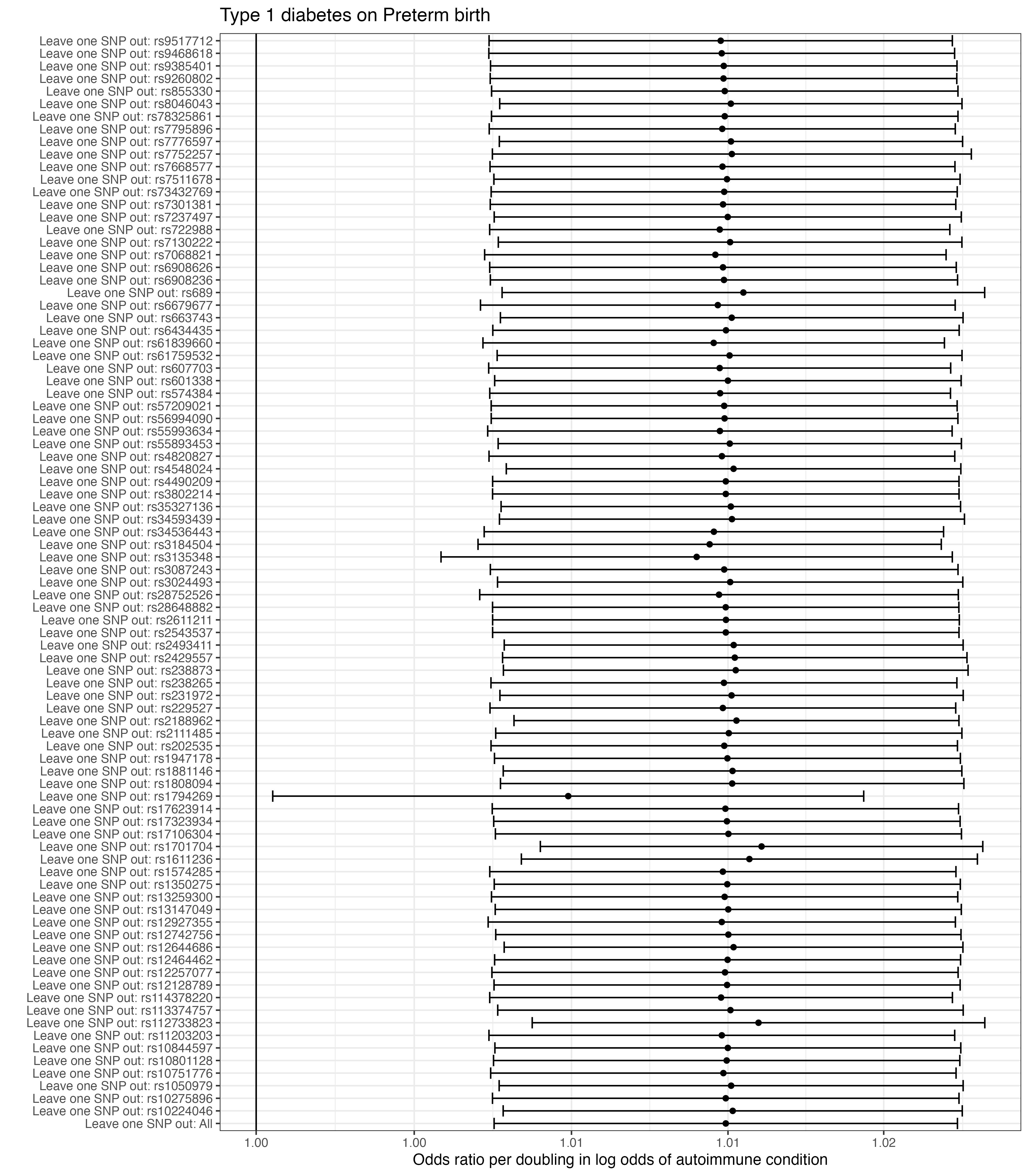


Figure 45. Leave-one-SNP-out Mendelian randomization estimates for the effect of type 1 diabetes on preterm birth. Estimates reflect odds ratio of pregnancy outcome per doubling in log odds of autoimmune condition.


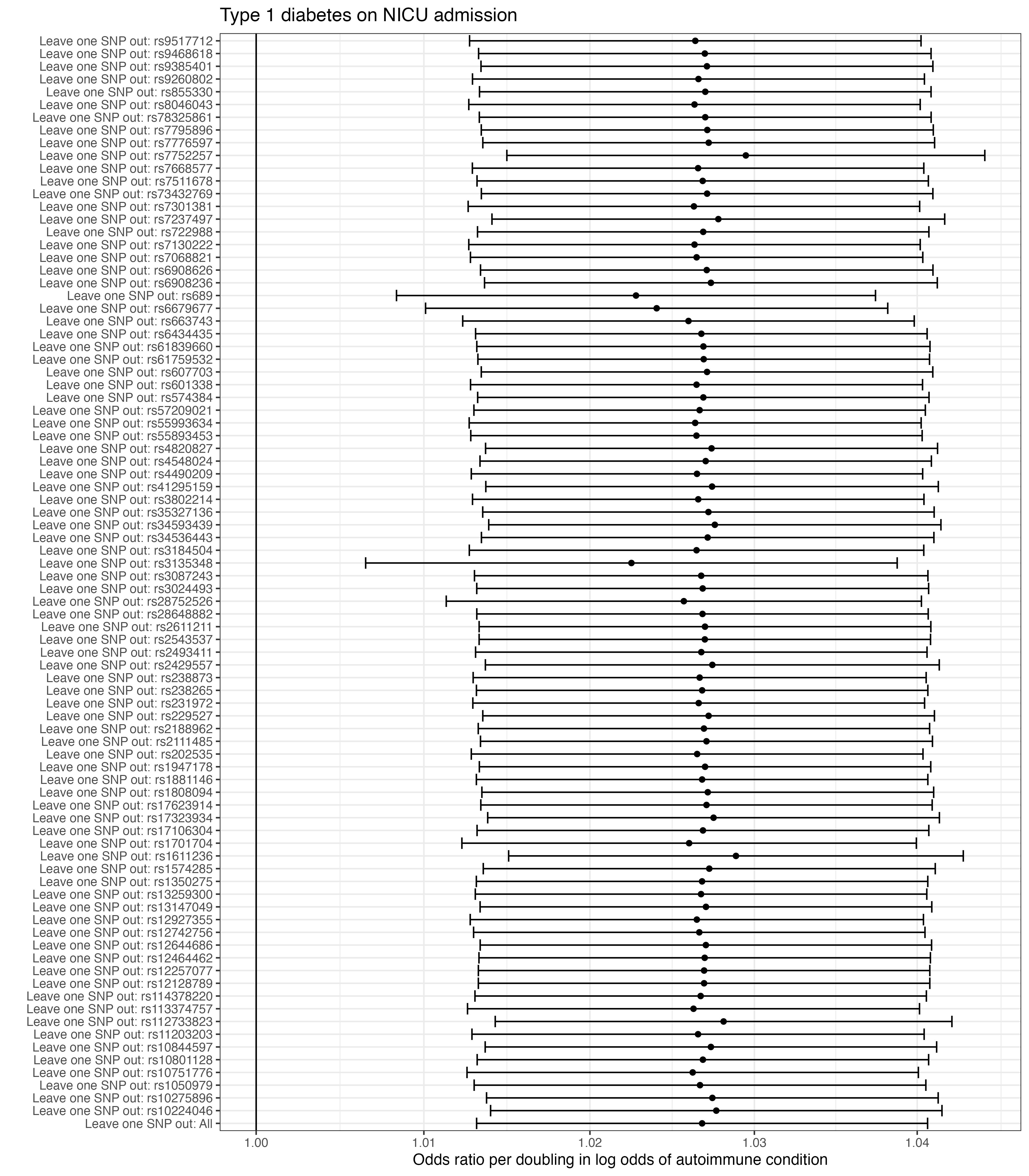


Figure 46. Leave-one-SNP-out Mendelian randomization estimates for the effect of type 1 diabetes on NICU admission. Estimates reflect odds ratio of pregnancy outcome per doubling in log odds of autoimmune condition.


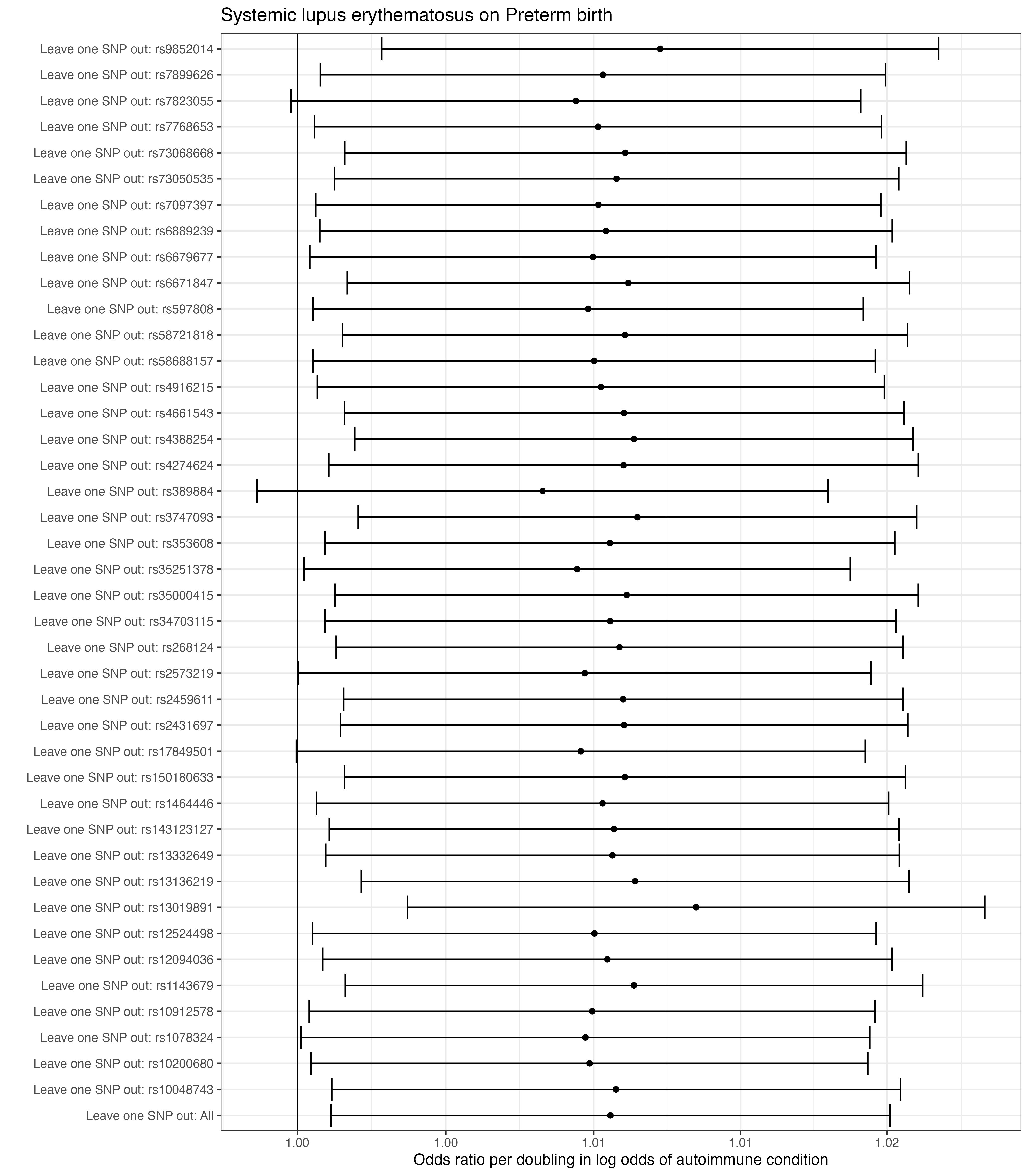


Figure 47. Leave-one-SNP-out Mendelian randomization estimates for the effect of systemic lupus erythematosus on preterm birth. Estimates reflect odds ratio of pregnancy outcome per doubling in log odds of autoimmune condition.


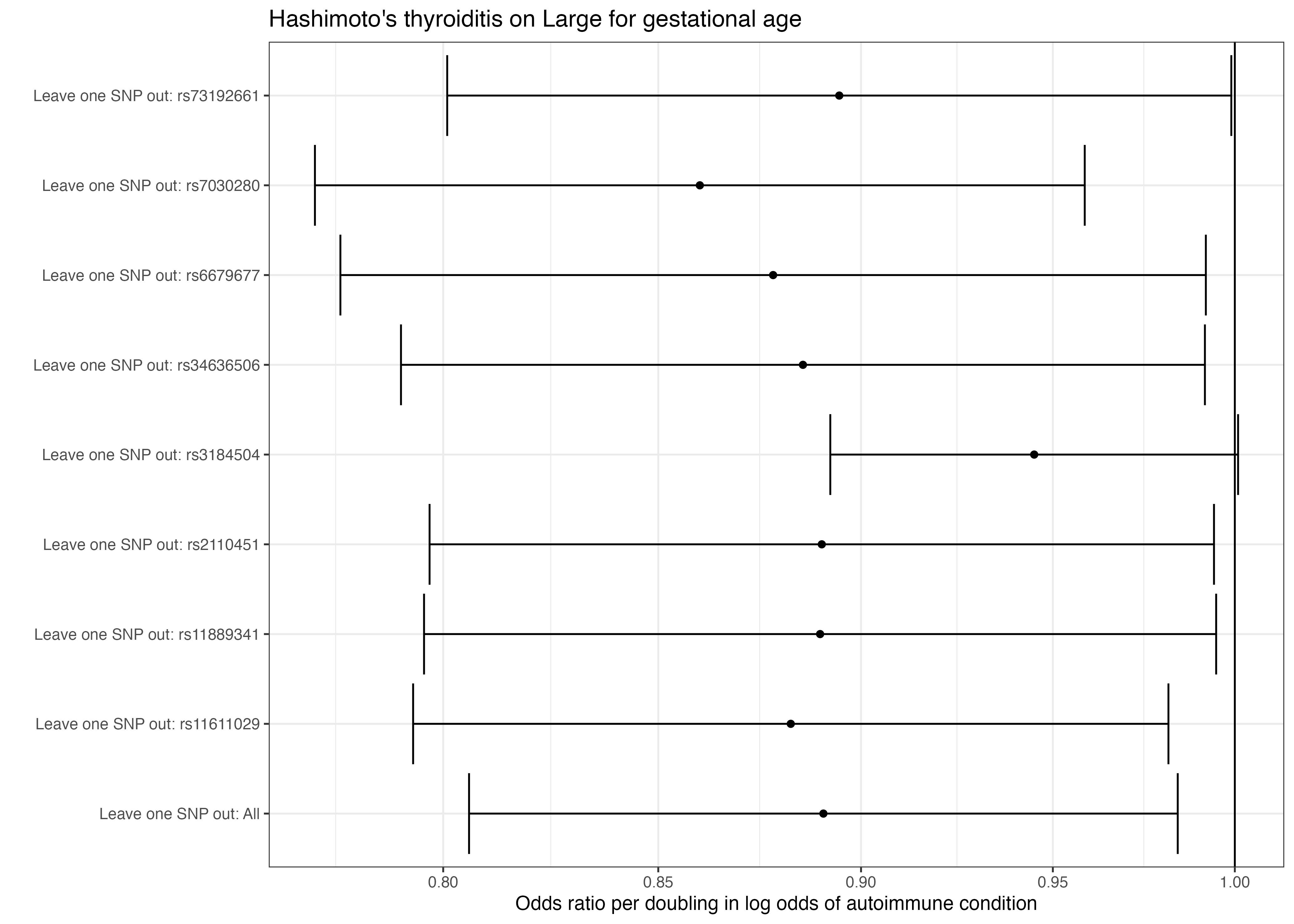


Figure 48. Leave-one-SNP-out Mendelian randomization estimates for the effect of Hashimoto’s thyroiditis on large-for-gestational-age. Estimates reflect odds ratio of pregnancy outcome per doubling in log odds of autoimmune condition.


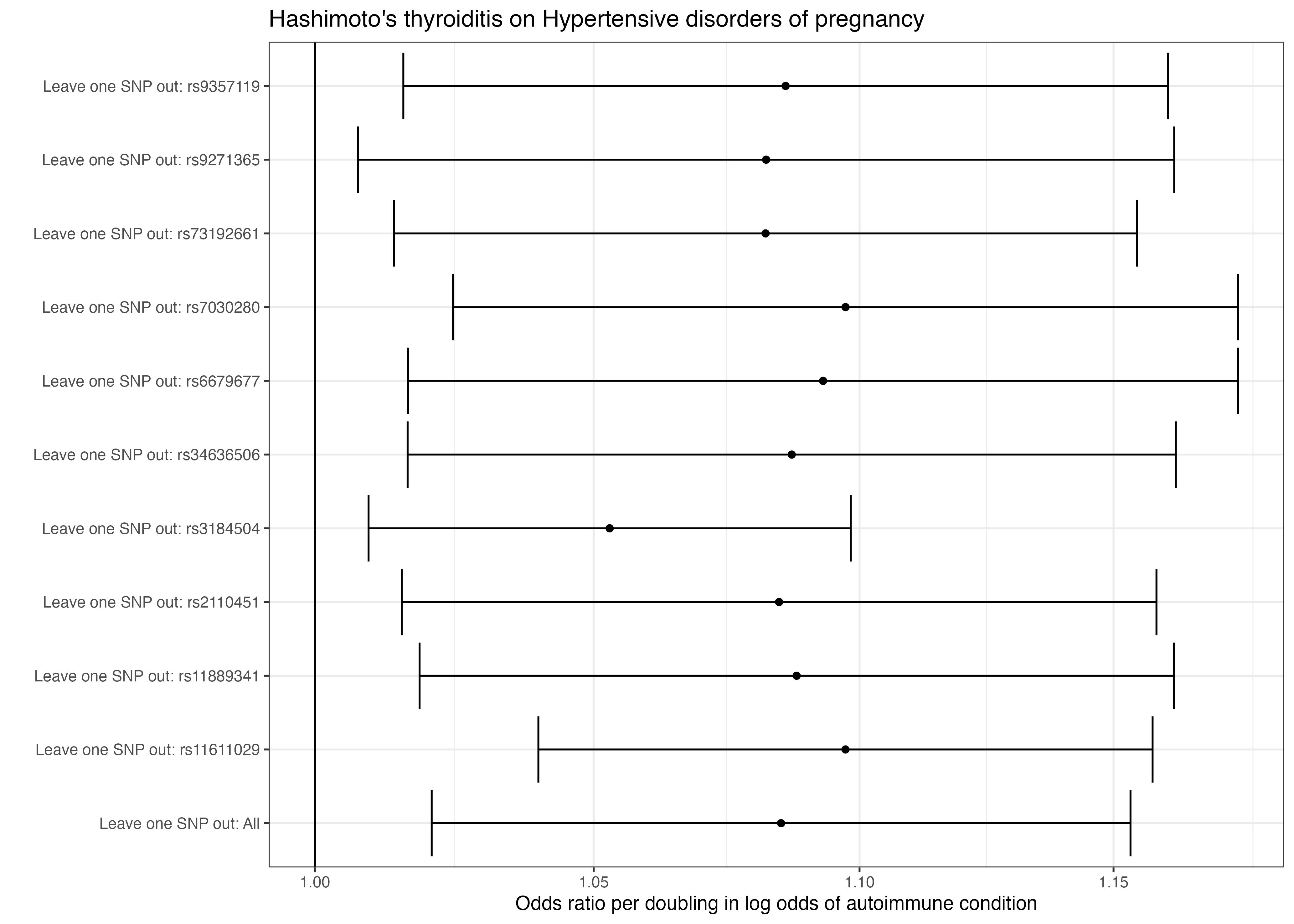
Figure 49. Leave-one-SNP-out Mendelian randomization estimates for the effect of Hashimoto’s thyroiditis on hypertensive disorders of pregnancy. Estimates reflect odds ratio of pregnancy outcome per doubling in log odds of autoimmune condition.


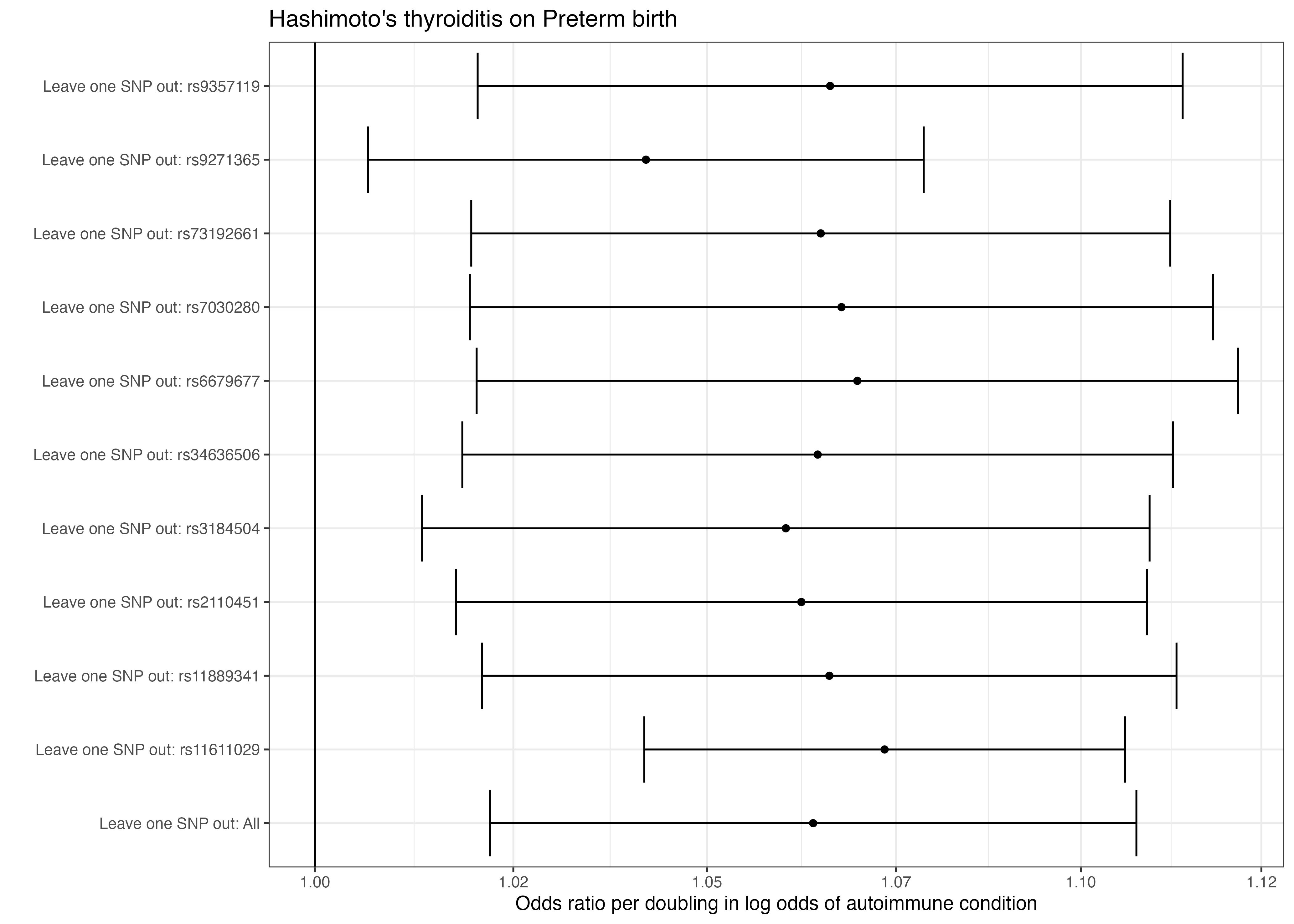


Figure 50. Leave-one-SNP-out Mendelian randomization estimates for the effect of Hashimoto’s thyroiditis on preterm birth. Estimates reflect odds ratio of pregnancy outcome per doubling in log odds of autoimmune condition.


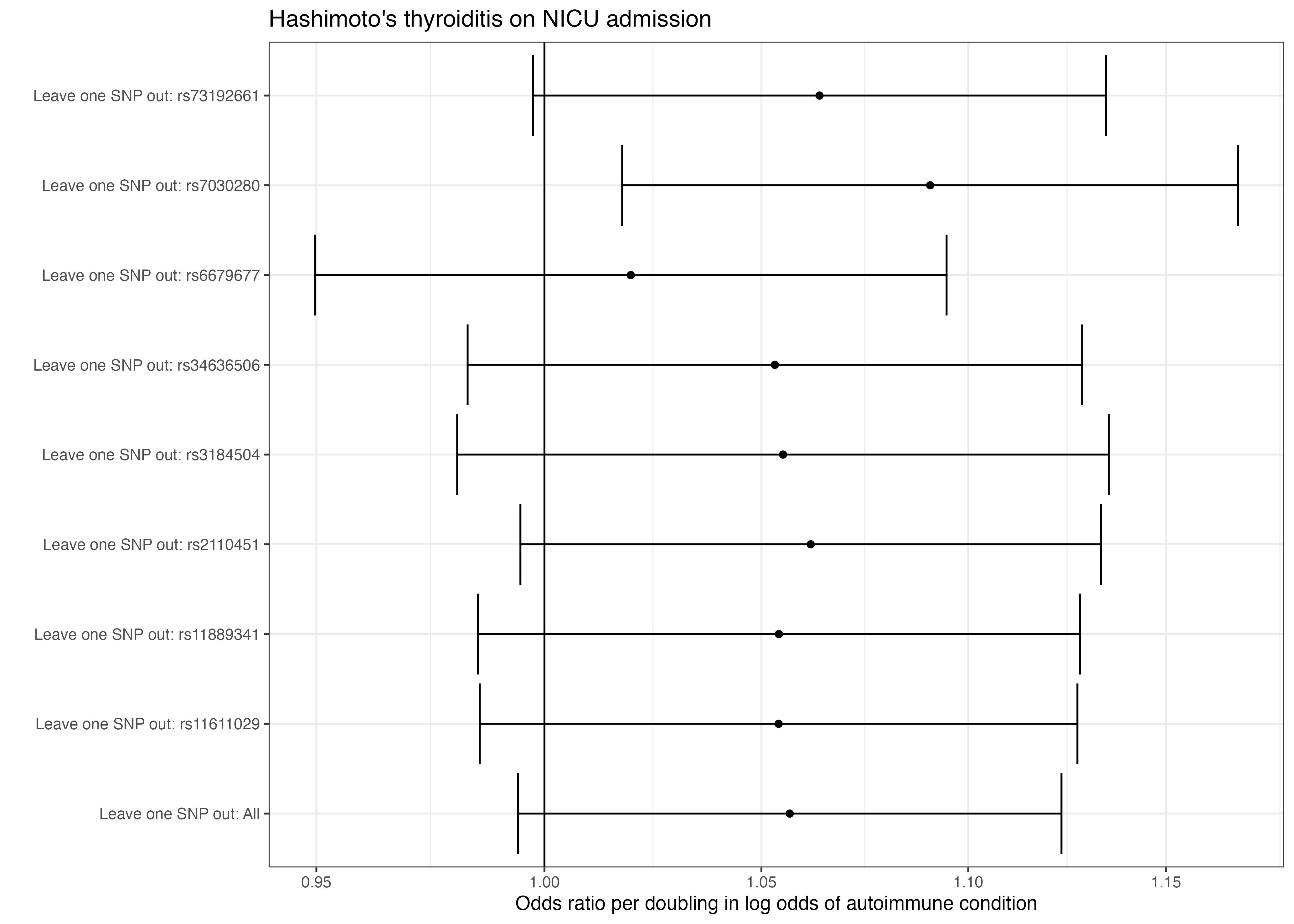


Figure 51. Leave-one-SNP-out Mendelian randomization estimates for the effect of Hashimoto’s thyroiditis on NICU admission. Estimates reflect odds ratio of pregnancy outcome per doubling in log odds of autoimmune condition.


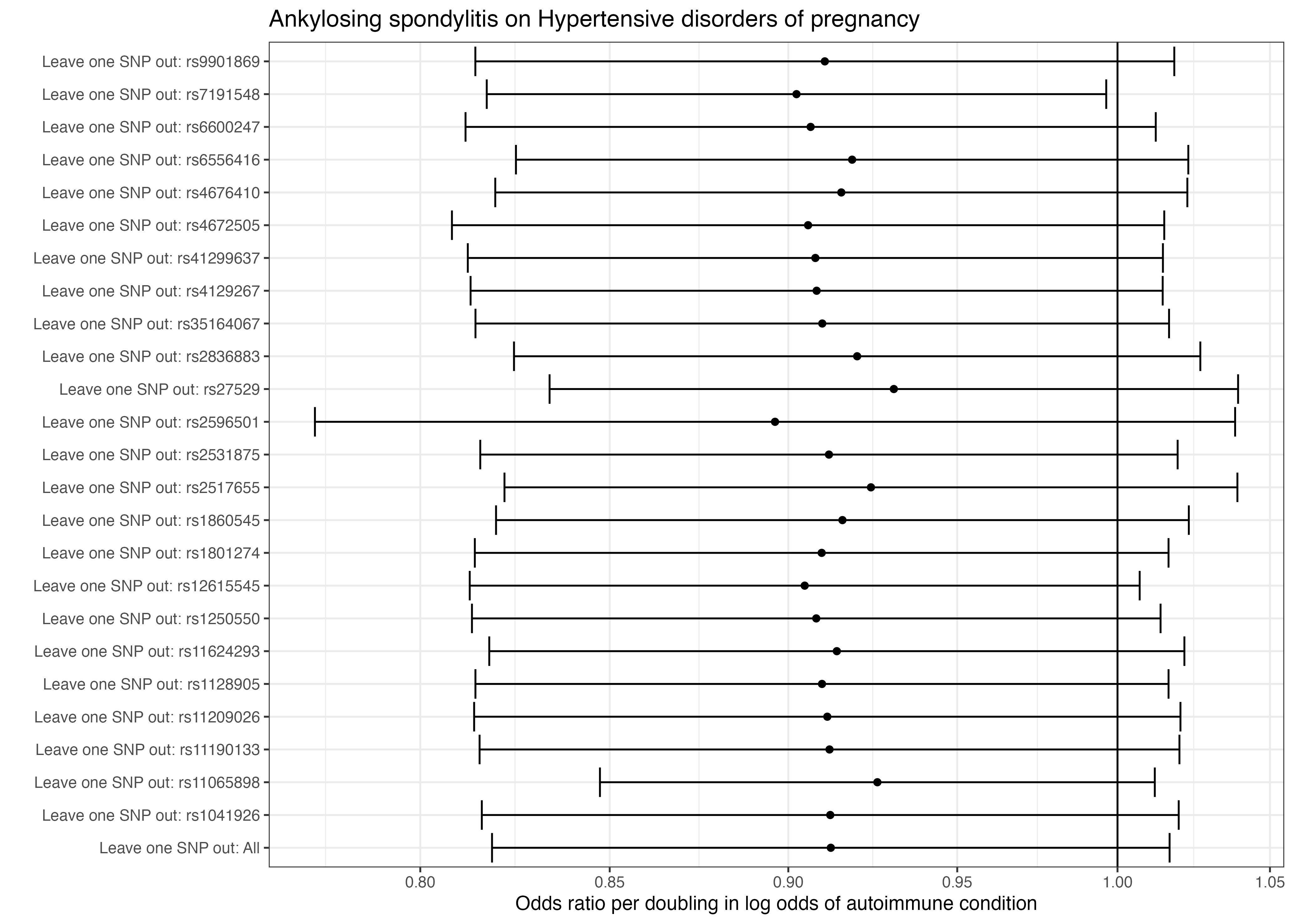
Figure 52. Leave-one-SNP-out Mendelian randomization estimates for the effect of ankylosing spondylitis on hypertensive disorders of pregnancy. Estimates reflect odds ratio of pregnancy outcome per doubling in log odds of autoimmune condition.


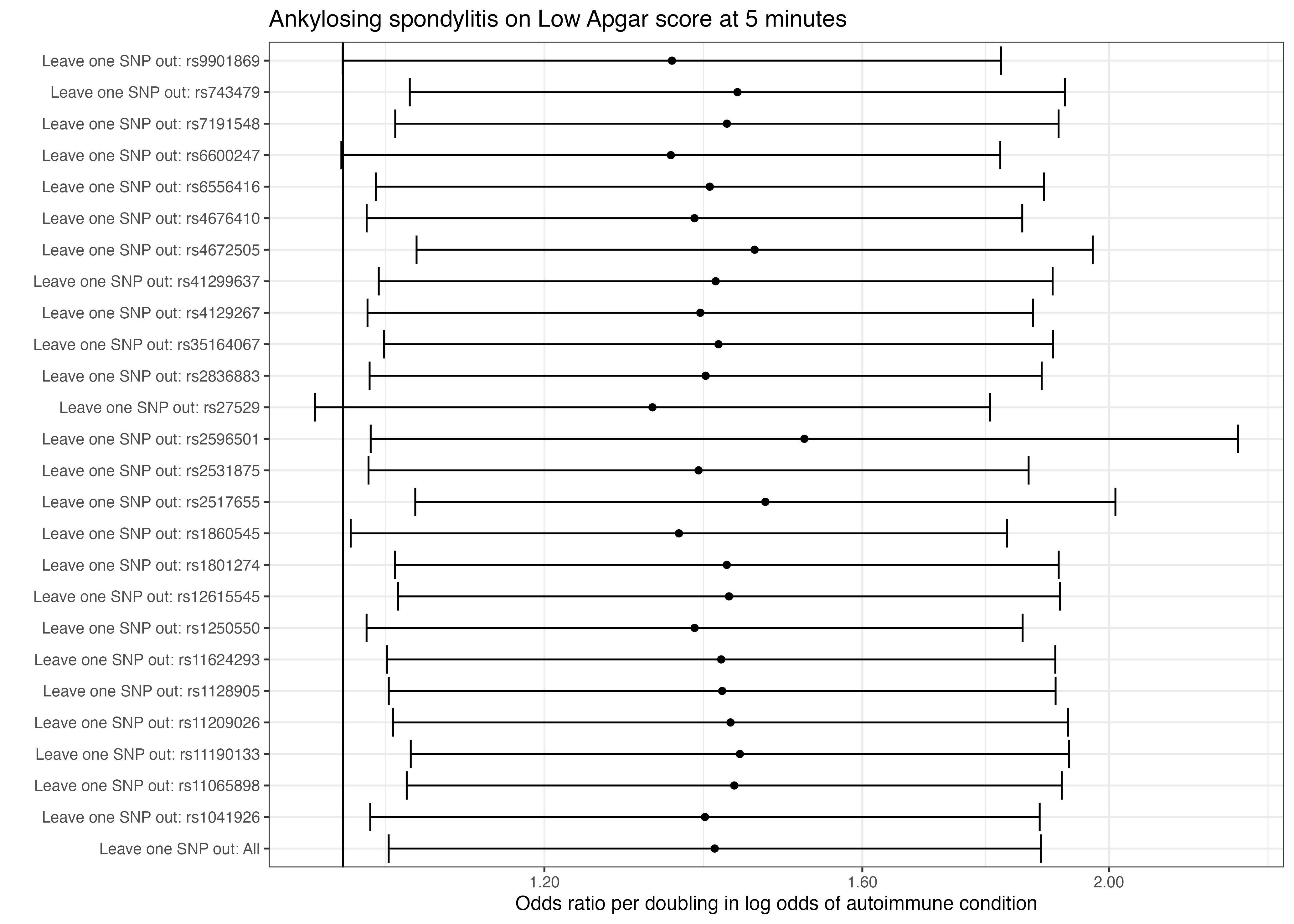


Figure 53. Leave-one-SNP-out Mendelian randomization estimates for the effect of ankylosing spondylitis on low Apgar score at 5 minutes. Estimates reflect odds ratio of pregnancy outcome per doubling in log odds of autoimmune condition.

## Phenome wide scan for influential SNPs


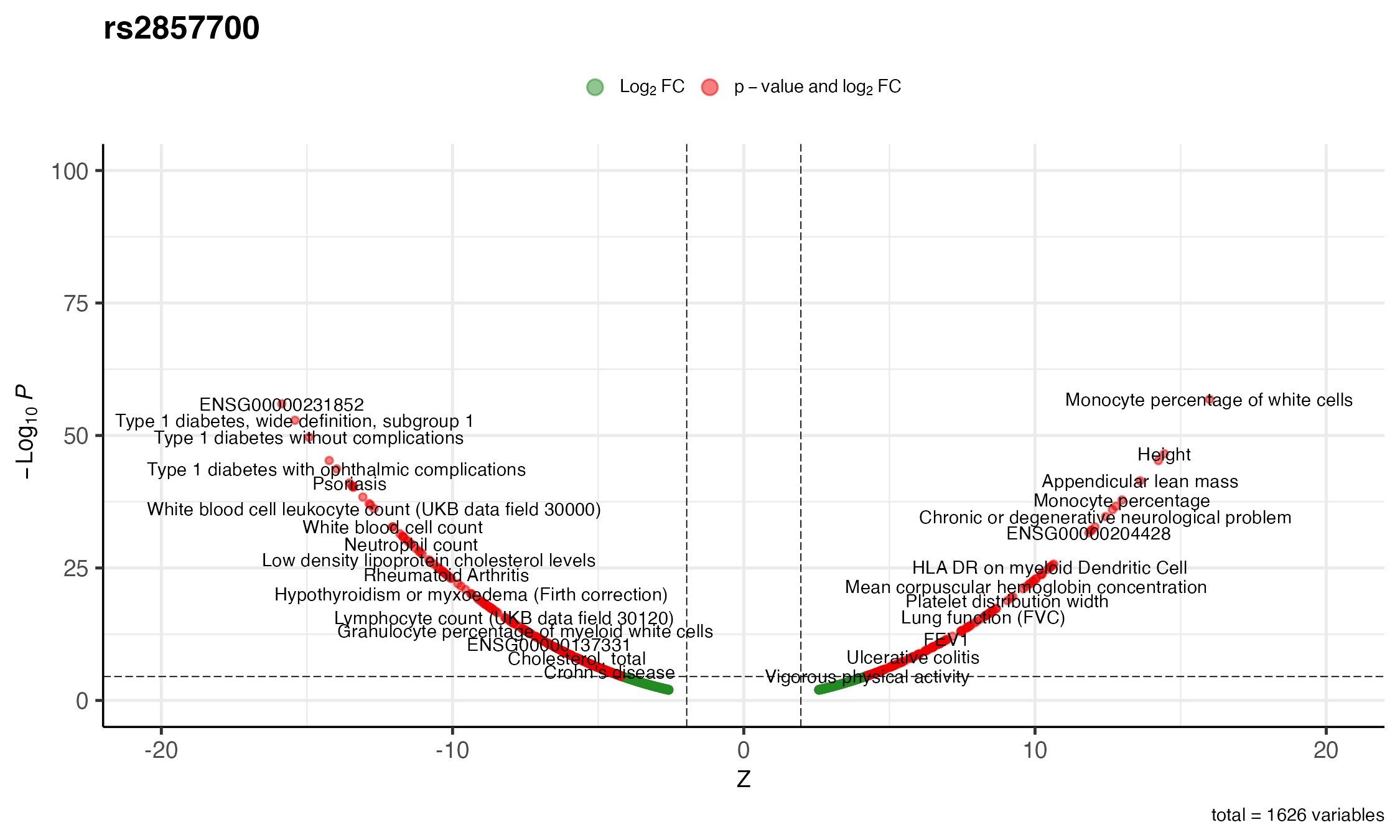


Figure 54. Phenome scan results for rs2857700, instrumenting multiple sclerosis.


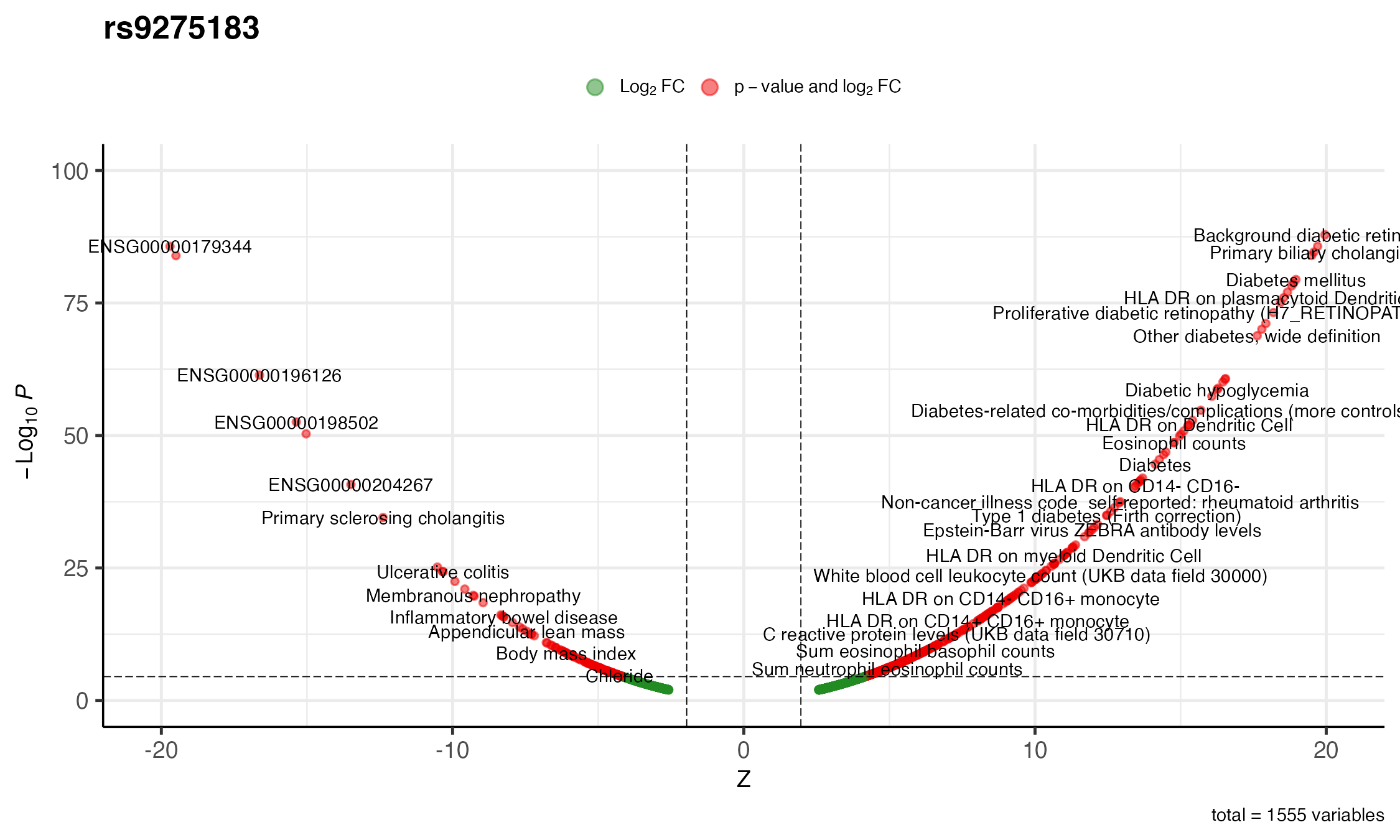


Figure 55. PheWAS results for rs9275183, instrumenting rheumatoid arthritis.


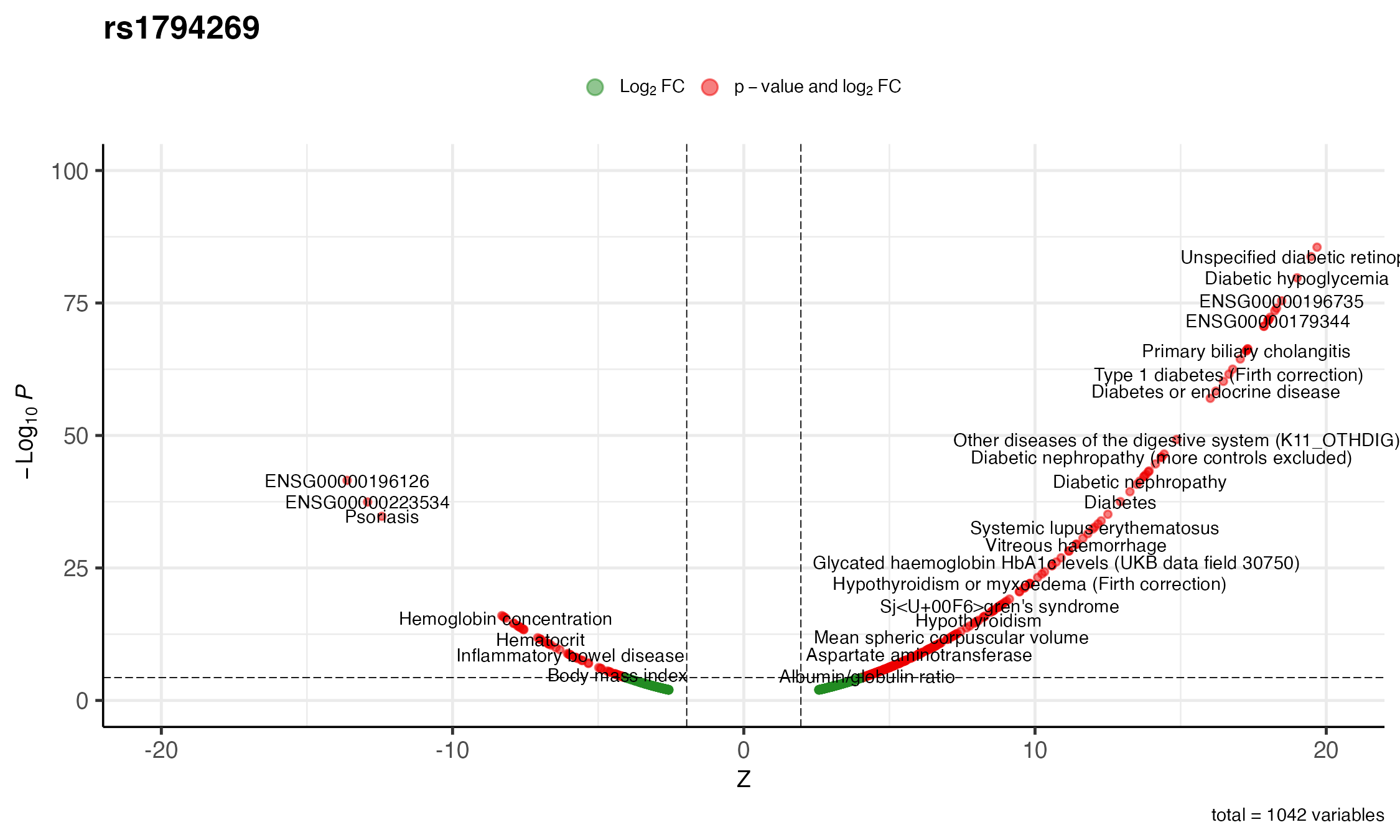


Figure 56. PheWAS results for rs1794269, instrumenting type 1 diabetes.


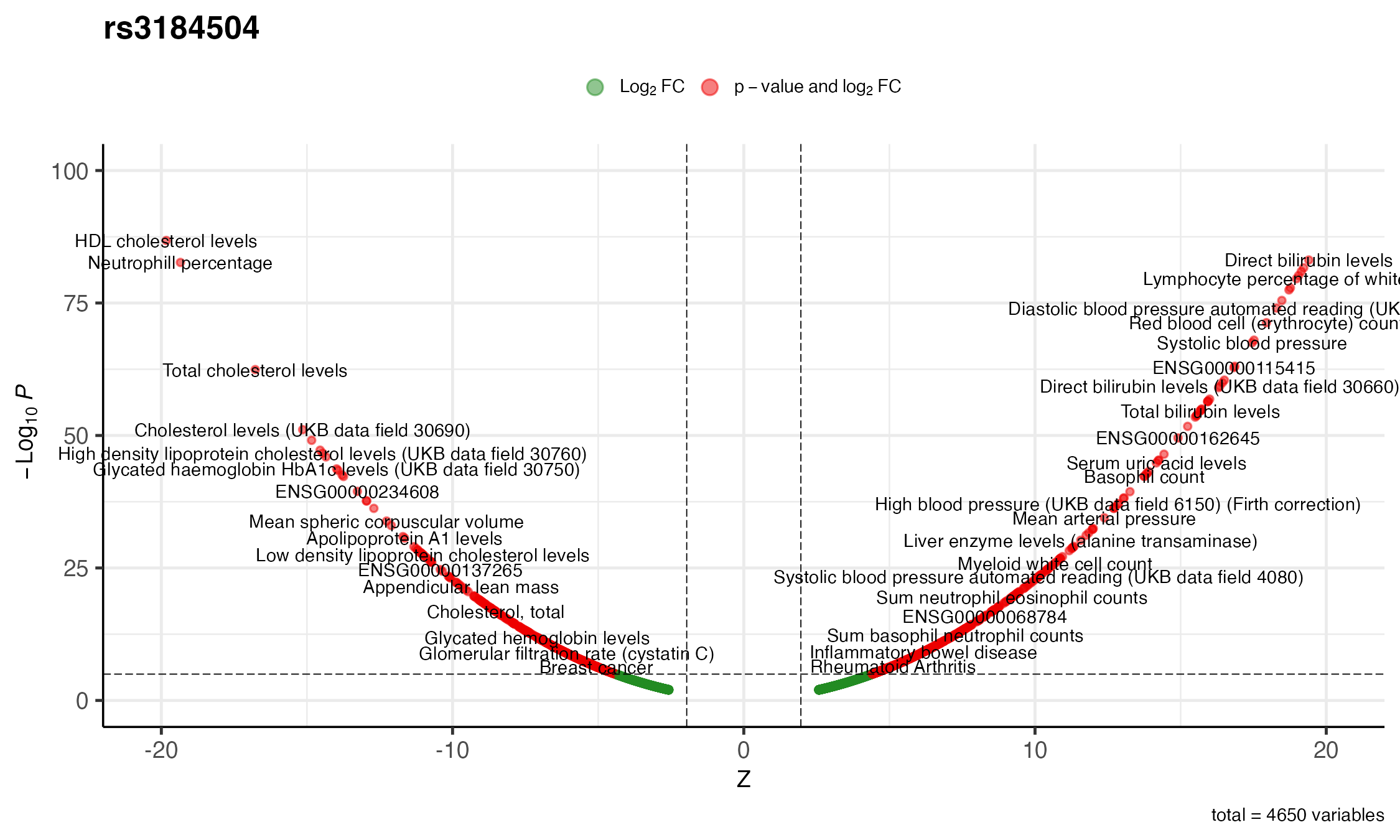


Figure 57. PheWAS results for rs3184504, instrumenting Hashimoto’s thyroiditis.


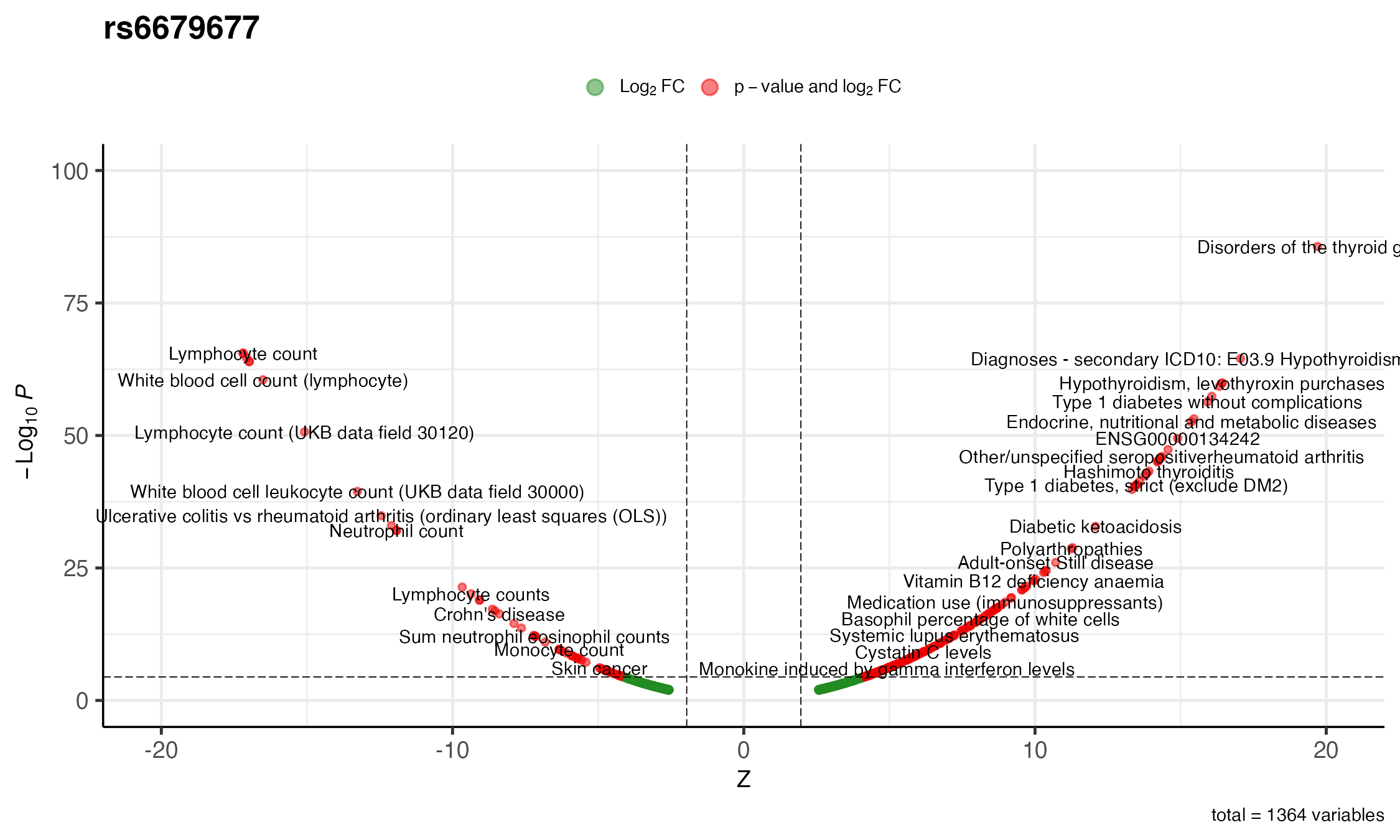


Figure 58. PheWAS results for rs6679677, instrumenting Hashimoto’s thyroiditis.

# Supplementary Methods

## Instrument strength

We estimated instrument strength using the equation:

$$F={(\frac{beta}{standard error})}^{2}$$

This form was used instead of versions requiring effect allele frequency information since this was not available for all genetic variants in the selected exposure GWAS.

## Instrument correlations between conditions

We explored the extent to which instrument associations were correlated across conditions. We extracted the 515 unique genetic instruments from the full GWAS summary statistics for each condition. The genotyping chip used and imputation varied between these GWAS, so SNPs available for extraction differed, and we excluded missing SNPs. We also excluded systemic sclerosis from these analyses since the full summary statistics are not made available. We harmonised all extracted associations to use the same effect allele as used in main MR analyses, ensuring that for each SNP effects across all conditions reflected the same effect allele. We then illustrated the correlations between SNP-condition betas (log odds scale) in scatter plots and calculated Pearson’s correlation coefficient. Where 2 or fewer SNPs were available we did not calculate the correlation coefficient. These correlations were estimated twice, first including instruments located in the HLA region and next after excluding these. We summarised the correlation coefficients in heatmaps as **Figures S2-S3**, and present scatter plots for all comparisons in **Figures S4-S6** (all **Additional file 2**).

## Weighted linear models to explore fetal genetic effects

To estimate effects of maternal genetic instruments on outcomes while accounting for fetal genetic effects, we used a weighted linear model (WLM). The WLM enables estimation of the mutually adjusted effects of maternal and fetal genotype on the outcomes, which are calculated using the summary-level association data from unadjusted GWAS (in which the outcomes were regressed on maternal or fetal genotype separately)[16-19]. Unlike a traditional approach, in which the outcome is regressed on maternal and fetal genotype jointly in a sample with both maternal and fetal genotype data available, the unadjusted GWAS used for the WLM require only maternal or fetal genotype data and can be fitted on separate (potentially overlapping) samples. This enables more efficient use of the available data.

To generate the WLM adjusted genetic estimates within the MR-PREG collaboration, we first generated unadjusted GWAS by regressing the outcomes separately on maternal and fetal genotype (and covariates including genetic principal components[15]. The MR-PREG unadjusted maternal GWAS used data from five cohort studies and two publicly available GWAS consortium datasets. The MR-PREG unadjusted fetal GWAS used data from three cohort studies (ALSPAC, BiB and MoBa). We then implemented the WLM using the DONUTS R package[18], to estimate i) maternal associations adjusted for offspring genotype and ii) fetal associations adjusted for maternal genotype. These models accounted for sample overlap between the maternal and fetal GWAS using an intercept term from bivariate LD score regression[20] of the maternal and fetal GWAS. The underlying equations are outlined in the MR-PREG collaboration profile supplementary material [15].

In our study no exposure instruments for systemic sclerosis (n=12 SNPs) were available in the fetal adjusted outcome GWAS, so we were unable to conduct the sensitivity analysis adjusting for fetal genotype for systemic sclerosis.

## Sample overlap

To estimate potential sample overlap we identified which exposure GWAS contained cohorts overlapping with MR PREG studies. For most exposure GWAS, there was no possible overlap, but GWAS for Hashimoto’s thyroiditis and type 1 diabetes both contained UK Biobank and FinnGen. We used information provided in the exposure GWAS on cases and controls from each contributing cohort [21,22], and the MR-PREG sample details (**Additional file 3: Tables S2A-B**), to estimate potential sample overlap. Since different cohorts contribute to each MR-PREG outcome, the proportion of overlap varied between outcomes and so was estimated separately. Where the exposure GWAS had provided the proportion of participants who were female, we assumed that this proportion of cases were also female and may overlap with our outcome GWAS sample. Where this proportion was not reported we assumed 0.5 were female. We estimated the maximal sample overlap between cases for binary outcomes, and between the whole sample for continuous outcomes. We reported sample overlap as both 1) exposure GWAS as a percentage of the outcome GWAS, and 2) outcome GWAS as a percentage of the exposure GWAS. Sample overlap should be reported with respect to the larger dataset [23], which varied here between exposure-outcome pairs, so we have additionally reported the smaller percentage of these two.

## Cohort descriptions

### Avon Longitudinal Study of Parents and Children (ALSPAC)

Pregnant women resident in Avon, UK with expected dates of delivery between 1^st^ April 1991 and 31^st^ December 1992 were invited to take part in the study[24,25]. 20,248 pregnancies have been identified as being eligible and the initial number of pregnancies enrolled was 14,541 (ALSPAC-G0), and gave birth to a total of 14,676 foetuses (ALSPAC-G1). These resulted in 14,062 live births and 13,988 children who were alive at 1 year of age. The total sample size for analyses using any data collected after the age of seven is therefore 15,447 pregnancies, resulting in 15,658 foetuses. Of these 14,901 children were alive at 1 year of age. A total of 14,203 unique mothers were initially enrolled in the study, and after additional recruitment a total of 14,833 unique women (ALSPAC-G0 mothers) were enrolled in ALSPAC as of September 2021. Here, we integrate ALSPAC data on these G0 pregnancies from: genetic data for mothers and children, four questionnaires collected during pregnancy, and linked obstetric records.

### Born in Bradford (BiB)

BiB prospectively recruited pregnant women in Bradford with expected dates of delivery between March 2007 and December 2010[26]. Recruitment for most women was at their oral glucose tolerance test, offered to all women booked for delivery at Bradford Royal Infirmary at around 26-28 weeks gestation. A total of 12,453 women (13,776 pregnancies) were enrolled during pregnancy, who gave birth to 13,858 live children. In this study, we used BiB genetic data collected from both mothers and children, and pregnancy outcome information retrieved through questionnaires and routinely linked primary and secondary care records.

### Norwegian Mother, Father and Child Cohort Study (MoBa)

MoBa prospectively recruited pregnant women across Norway between 1999 and 2008. The cohort has recruited approximately 95,200 mothers, 75,200 fathers and 114,500 children. Here, we used genetic data for mothers and children, alongside questionnaires collected during pregnancy and postpartum, and data from the Medical Birth Registry (MBRN) which is a national health registry which holds information on all births in Norway.

### FinnGen

FinnGen is a national network of Finnish biobanks linked to electronic registry data. It includes data on 500,348 individuals (282,064 females; 12^th^ data release). In this study, we used FinnGen’s publicly available genetic association data, since the cohort has both genetic data and pregnancy outcome information available as clinical endpoints (encoded by ICD9-10 codes; see <https://www.finngen.fi/en/access_results>).

### UK Biobank (UKB)

The UK Biobank is a cohort study which recruited adults aged 40-69 years registered with the UK National Health Service (NHS) and living within a 25-mile radius of one of the 22 study centres [27]. Participants were invited between 2006 and 2010, with a 5.5% response rate resulting in 500,000 adults being recruited (54.4% female). Here, we used genetic data, self-completed questionnaires, and linked electronic health records including Hospital Episode Statistics (HES) from 1997 for England, 1998 for Wales and 1981 for Scotland; HES also includes maternity-related admissions for England and Wales.

Details on genotyping arrays and imputation for ALSPAC, BiB, MoBa and UKB are provided in the MR-PREG collaboration profile supplementary material [15].

## Cohort acknowledgments

### Avon Longitudinal Study of Parents and Children (ALSPAC)

We are extremely grateful to all the families who took part in this study, the midwives for their help in recruiting them, and the whole ALSPAC team, which includes interviewers, computer and laboratory technicians, clerical workers, research scientists, volunteers, managers, receptionists and nurses.

### Born in Bradford (BiB)

BiB is only possible because of the enthusiasm and commitment of the Children and Parents in BiB. We are grateful to all the participants, teachers, school staff, health professionals and researchers who have made BiB happen.

### Norwegian Mother, Father and Child Cohort Study (MoBa)

This research has been conducted using MoBa data using application number 2552. MoBa is supported by the Norwegian Ministry of Health and Care services and the Ministry of Education and Research. We are grateful to all the participating families in Norway who take part in this on-going cohort study. We thank the Norwegian Institute of Public Health (NIPH) for generating high-quality genomic data. This research is part of the HARVEST collaboration, supported by the Research Council of Norway (#229624). We also thank the NORMENT Centre for providing genotype data, funded by the Research Council of Norway (#223273), South East Norway Health Authority and KG Jebsen Stiftelsen. We further thank the Center for Diabetes Research, the University of Bergen for providing genotype data and performing QC and imputation of the data funded by the ERC AdG project SELECTionPREDISPOSED, Stiftelsen Kristian Gerhard Jebsen, Trond Mohn Foundation, the Research Council of Norway, the Novo Nordisk Foundation, the University of Bergen, and the Western Norway Health Authorities (Helse Vest).

### FinnGen

The authors thank the FinnGen investigators for sharing their summary-level data.

### UK Biobank (UKB)

We would like to thank all the participants of UK Biobank for their vital contribution to the resource. This research has been conducted using the UK Biobank Resource under Application Number 23938.

## Cohort funding

### Avon Longitudinal Study of Parents and Children (ALSPAC)

The UK Medical Research Council and Wellcome (Grant ref: 217065/Z/19/Z) and the University of Bristol provide core support for ALSPAC. A comprehensive list of grants funding is available on the ALSPAC website

(<http://www.bristol.ac.uk/alspac/external/documents/grant-acknowledgements.pdf>). ALSPAC GWAS data was generated by Sample Logistics and Genotyping Facilities at Wellcome Sanger Institute and LabCorp (Laboratory Corporation of America) using support from 23andMe. This research was funded in part by the Wellcome Trust (Grant ref: 228276/Z/23/Z)]. For the purpose of Open Access, the author has

applied a CC BY public copyright licence to any Author Accepted Manuscript version arising from this submission.

### Born in Bradford (BiB)

BiB receives core funding from the Wellcome Trust (WT101597MA), a joint grant from the UK Medical and Economic and Social Science Research Councils (MR/N024397/1), British Heart Foundation (CS/16/4/32482), and the National Institute of Health Research under its Applied Research Collaboration for Yorkshire and Humber and Clinical Research Network research delivery support. Further support for genome-wide and multiple ‘omics measurements in BiB is from the UK Medical Research Council (G0600705), National Institute of Health Research (NF-SI-0611-10196), US National Institute of Health (R01DK10324), and the European Research Council under the European Union’s Seventh Framework Programme (FP7/2007–2013) / ERC grant agreement no 669545.

### Norwegian Mother, Father and Child Cohort Study (MoBa)

MoBa funding is under **Acknowledgements** as requested by MoBa publication guidelines.

### UK Biobank (UKB)

UK Biobank is funded primarily by the Wellcome Trust and the Medical Research Council (MRC). It is also funded by the Department of Health, British Heart Foundation, Cancer Research UK, Diabetes UK, National Institute for Health Research (NIHR), Scottish Government, Northwest Regional Development Agency, and Welsh Assembly Government.

## Cohort ethical approval

### Avon Longitudinal Study of Parents and Children (ALSPAC)

Ethical approval for the study was obtained from the ALSPAC Ethics and Law Committee (IRB00003312) and the Local Research Ethics Committees. Consent for biological samples has been collected in accordance with the Human Tissue Act (2004).

Ethical approval was obtained from the ALSPAC Ethics and Law Committee and the Local Research Ethics Committees. Consent for biological samples has been collected in accordance with the Human Tissue Act (2004). Informed consent for the use of data collected via questionnaires and clinics was obtained from participants following the recommendations of the ALSPAC Ethics and Law Committee at the time (details and reference numbers of all ethics approvals can be found at <http://www.bristol.ac.uk/medialibrary/sites/alspac/documents/governance/Research%20Ethics%20Committee%20approval%20references.pdf>).

### Born in Bradford (BiB)

Ethical approval for the study was granted by the Bradford National Health Service Research Ethics Committee (ref 06/Q1202/48), and all participants gave written informed consent. The ALL IN sub-study had ethical approval from the London School of Hygiene & Tropical Medicine ethics committee (ref: 5320) and the Bradford Research Ethics committee (ref: 08/H1302/21). Parents (usually the mother) gave informed, written consent to take part in the study.

### Norwegian Mother, Father and Child Cohort Study (MoBa)

The current study is based on version 12 of the quality-assured data files released for research in 2019. The establishment of MoBa and initial data collection was based on a license from the Norwegian Data Protection Agency and approval from The Regional Committees for Medical and Health Research Ethics. The MoBa cohort is currently regulated by the Norwegian Health Registry Act. The current study was approved by The Regional Committees for Medical and Health Research Ethics of South/East Norway (ref 2018/1256).

### UK Biobank (UKB)

The UK Biobank has approval from the North West Multi-centre Research Ethics Committee (MREC) as a Research Tissue Bank (RTB) approval. This RTB approval was granted initially in 2011 (11/NW/0382) and it is renewed on a 5-yearly cycle, with the latest one successfully renewed in 2021 (21/NW/0157).
